# Role of immunosuppressive JNK pathway in the tumor microenvironment among TNBC subtypes in IBCSG trial 22-00

**DOI:** 10.1016/j.isci.2025.112964

**Published:** 2025-06-20

**Authors:** Andrea Joaquin Garcia, Takashi Semba, Mattia Rediti, Daniel J. McGrail, Xuemei Xie, Xiaoping Wang, Dileep R. Rampa, David Venet, Laurence Buisseret, Samira Majjaj, Roswitha Kammler, Marco Colleoni, Sherene Loi, Giuseppe Viale, Meredith M. Regan, Françoise Rothé, Christos Sotiriou, Naoto T. Ueno

**Affiliations:** 1 Breast Cancer Translational Research Laboratory, Institut Jules Bordet, Université Libre de Bruxelles, Brussels, Belgium; 2 Section of Translational Breast Cancer Research, The University of Texas MD Anderson Cancer Center, Houston, TX, USA; 3 Department of Breast Medical Oncology, The University of Texas MD Anderson Cancer Center, Houston, TX, USA; 4 Division of Carcinogenesis, The Cancer Institute, Japanese Foundation for Cancer Research, Tokyo, Japan; 5 Lerner Research Institute, Cleveland, OH, USA; 6 Cancer Biology Program, University of Hawaiʻi Cancer Center, Honolulu, HI, USA; 7 Translational Research Coordination International Breast Cancer Study Group, Division of ETOP IBCSG Partners Foundation, Bern, Switzerland; 8 International Breast Cancer Study Group, Division of Medical Senology, IEO, European Institute of Oncology IRCCS, Milan, Italy; 9 International Breast Cancer Study Group, Peter MacCallum Cancer Centre, University of Melbourne, Melbourne, VIC, Australia; 10 IEO European Institute of Oncology IRCCS, Milan, Italy; 11 International Breast Cancer Study Group Statistical Center, Dana-Farber Cancer Institute, Harvard Medical School, Boston, MA, USA; 12 IFOM ETS, the AIRC Institute of Molecular Oncology, Milan, Italy

**Keywords:** Immunology, Cancer, Transcriptomics

## Abstract

Phosphorylation of the JNK (pJNK) protein promotes an immunosuppressive tumor microenvironment (TME), enhancing aggressiveness in inflammatory triple-negative breast cancer (TNBC). This study evaluated the role of JNK signaling using a gene signature. RNA sequencing was performed on 347 TNBC tumors from the phase 3 International Breast Cancer Study Group (IBCSG) 22-00 trial, which evaluated adjuvant low-dose cyclophosphamide and methotrexate (CM). Immune-related tumors were identified by TNBC subtype or tumor-infiltrating lymphocytes (TILs). Associations between JNK and outcomes were analyzed using Cox models. Low pJNK levels were associated with better disease-free survival (DFS) in immune-related tumors. These tumors also had lower Treg levels and higher CD8^+^/Treg ratios. Notably, immunomodulatory (IM) tumors with high pJNK showed improved DFS when treated with CM. High pJNK expression identifies immunosuppressive TMEs with poor prognosis in inflamed TNBC. However, these tumors may benefit from CM, supporting pJNK as a potential biomarker for immunotherapy strategies.

## Highlights

• Developed an RNA-based signature to estimate JNK phosphorylation in TNBC
• High pJNK levels correlate with immunosuppressive TME: more Tregs, lower CD8^+^/Treg ratios
• High pJNK immune tumors respond to low-dose CM, possibly reversing pJNK-driven suppression
• pJNK signature may guide immunotherapy strategies in TNBC

## Graphical abstract

**Figure:**
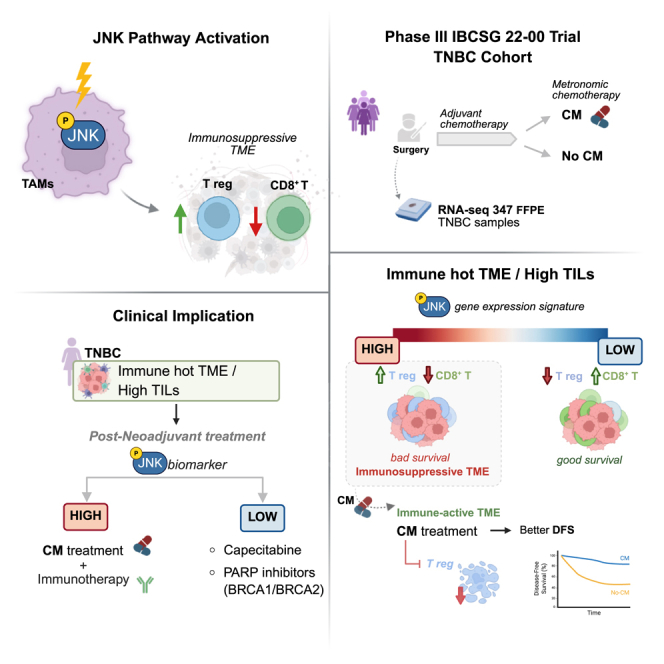

## Introduction

Triple-negative breast cancer (TNBC) represents 15%–20% of all breast cancer cases. It is defined by the lack of expression of estrogen receptor (ER), progesterone receptor, and human epithelial growth factor receptor 2 (HER2). Several molecular classifications have been described in the last decade to facilitate a more rational development of novel therapies.^1^^,^^2^^,^^3^^,^^4^^,^^5^ Bareche et al. refined the original Lehmann’s classification^1^ into five stable molecular subtypes, namely immunomodulatory (IM), basal-like (BL), luminal androgen receptor (LAR), mesenchymal (M), and mesenchymal-stem-like (MSL).^3^ These molecular subtypes have been characterized by distinct mutational profiles, genomic alterations, and biological processes.^3^ Moreover, different patterns of immune infiltration in the tumor microenvironment (TME) have also been observed.^6^

An immunosuppressive TME plays a crucial role in tumor progression. One key factor in its development is the presence of regulatory T cells (Tregs), which inhibit the cytotoxic functions and the proliferation of effector T cells, including CD8^+^ T cells.^7^^,^^8^^,^^9^^,^^10^ However, the specific molecular mechanisms that recruit Tregs through chemokines into the TME are largely unknown. We previously identified that c-Jun N-terminal kinase (JNK) pathway-related molecules, such as phosphorylated JNK and phosphorylated C-JUN, were enriched in the inflammation-related subtype of TNBC by unsupervised analysis of reverse phase protein array (RPPA).^11^ Moreover, our recent results showed that the phosphorylation of the JNK (pJNK) signaling pathway promotes the formation of an immunosuppressive TME in the TNBC.^12^ In particular, phosphorylated JNK (pJNK)-regulated chemokine CCL2 production from tumor-associated macrophages was shown to recruit immunosuppressive Tregs, increasing TNBC aggressiveness. Moreover, pJNK signaling is also involved in inflammation processes.^12^ Interestingly, pJNK inhibition converted an immunosuppressive TME to an immunoactive one and reduced tumor growth of multiple preclinical TNBC models, which may lead to new therapeutic strategies for TNBC.

Despite the efforts, chemotherapy was primarily the standard treatment choice for the majority of patients affected by TNBC.^13^ Immune checkpoint inhibitors (ICIs), PARP (Poly-ADP ribose polymerase) inhibitors, antibody-drug conjugates, and other drug combinations^14^^,^^15^^,^^16^^,^^17^^,^^18^ represent emerging therapeutic strategies that target new oncogenic vulnerabilities for this disease. In particular, the successful outcomes achieved with immunotherapy in early-stage TNBC has become the new standard of treatment, raising new questions about the ideal chemotherapy counterpart for ICIs.

We have previously demonstrated that TNBCs expressing an IM phenotype had better outcomes with 1-year adjuvant low-dose maintenance CM (cyclophosphamide and methotrexate) in the International Breast Cancer Study Group (IBCSG) trial 22-00^20^ (Figure S1). This trial, however, was negative for the whole population and for the TNBC cohort. Moreover, the presence of high levels of Tregs was significantly associated with a better outcome following CM. These results highlighted the positive impact of metronomic chemotherapy on this specific subgroup of patients and suggested a strong association between immune response and benefit from low-dose CM maintenance. However, further exploration is needed to identify the distinct immune-related pathways in TNBC and their association with responses to metronomic chemotherapy.

This study explores the prevalence and prognostic impact of the immunosuppressive pJNK pathway across different TNBC molecular subtypes in the context of the phase 3 IBCSG 22-00 trial. Furthermore, we evaluate the possible interactions of pJNK signaling among TNBC subtypes in relation to their response to maintenance therapy using low-dose CM.

## Results

### Survival impact of pJNK signature in TNBC molecular subtypes

The phosphorylation of JNK has been previously associated with the activation of immunosuppressive status in the TME of TNBC.^12^ To further investigate the significance of this biological process in TNBC, we developed a gene signature to estimate the phosphorylation levels of JNK using data from The Cancer Genome Atlas (TCGA) database, including matched RNA-seq and pJNK-targeted proteomic data. As a result, we identified a 16-gene signature (Tables S1) that exhibited a strong correlation with JNK phosphorylation levels, thus reflecting the status of JNK phosphorylation (Figure S2). The pJNK signature also showed a similar trend of correlation with phosphorylated JNK status in the independent TNBC phosphoproteomics data of the Clinical Proteomic Tumor Analysis Consortium (CPTAC)^20^ (Figure S2C). The small sample size of the CPTAC dataset (*n* = 12) is a significant limitation, highlighting the need for validation in a larger external dataset to assess more robustness of our findings.

To explore potential associations between the pJNK signature and clinical outcomes, we applied the pJNK signature to our TNBC cohort from the IBSCG 22-00 study.^33^ Patients were divided into low and high pJNK subgroups based on the median expression value of the pJNK signature across all tumor samples. We observed no significant difference in the estimated 5-year disease-free survival (DFS) rate between patients with low pJNK expression levels (81.4%) and those with high pJNK levels (75.5%) (Figure S3B) (hazard ratio [HR], 1.29; 95% confidential interval [CI], 0.8–2.09; *p* value = 0.15). Similar results were obtained for overall survival (OS), distant recurrence-free interval (DRFI), and breast cancer-free interval (BCFI) (Figures S3A–S3D).

We then classified TNBC samples into the five molecular subtypes based on Lehmann’s subtype.^3^ The tumor samples were distributed as follows: BL (*n* = 72), IM (*n* = 86), LAR (*n* = 47), M (*n* = 89), and MSL (*n* = 53) (Figure S4).

**Figure 1:**
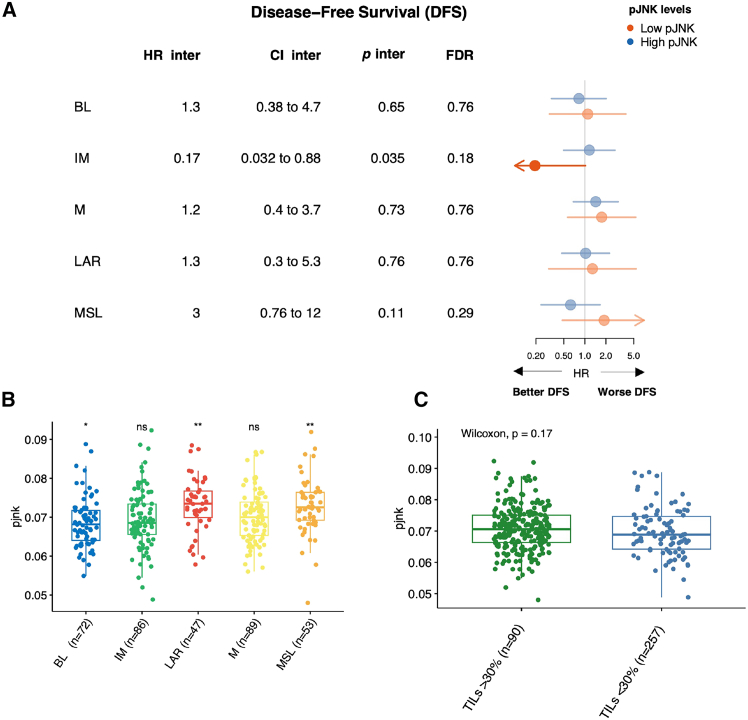
Survival analysis of pJNK levels across TNBC molecular subtypes and distribution of pJNK signature among molecular subtypes and TILs levels (A) Cox proportional-hazard analysis was performed to evaluate the associations of TNBC molecular subtypes with DFS, both overall and stratified by pJNK levels. In the right, hazard ratios (HRs) are shown for subgroups with low (blue dots) and high (yellow dots) pJNK levels. The HR inter represents the ratio of HRs between the low and high pJNK subgroups for each molecular subtype. *p* inter corresponds to a Wald test used to assess the statistical interaction between TNBC subtypes and pJNK levels. The low pJNK group includes samples with gene signature expression below the median, while the high pJNK group includes those with expression above the median. (B and C) Boxplot shows the distribution of JNK levels across the TNBC molecular subtypes (B) and TILs levels (C). Boxplot elements: median; box limits, upper and lower quartiles, and whiskers (5^th^ and 95^th^ percentile). *p* value was determined using pairwise Wilcoxon rank-sum test (one group vs. others), ∗*p* < 0.05 and ∗∗*p* < 0.005.

Notably, we found that among tumors with IM phenotype, those with low pJNK levels were associated with a lower risk for a DFS event than those with high pJNK levels (HR inter, 0.17; 95% CI, 0.032–.88; *p* inter = 0.035) (Figure 1A). This pattern was also observed for DRFI (Figure S5). However, the results were not significant for either DFS or DRFI after false discovery rate (FDR) correction. This relationship can be visualized in the Kaplan-Meier curves presented in Figures S6 and S7. Importantly, no statistically significant relations with DFS, overall or by pJNK expression, were observed for tumors classified into the other molecular subtypes (Figure 1A).

Interestingly, we found that pJNK signature levels were significantly higher in the LAR and MSL subtypes (Figure 1B), and similar trend was also observed in the I-SPY2 trial cohort^21^ (Figure S7). These findings are consistent with previous studies associating LAR and MSL subtypes with immunosuppressive pathways and a prevalence of stromal processes.^6^

To further investigate the association of pJNK and the IM phenotype with outcome, we explore the connection between pJNK signature and different levels of tumor-infiltrating lymphocytes (TILs) (high [>30%] vs. low) in relation to DFS. We found that tumors with high levels of TILs exhibited better DFS for low pJNK levels compared to those with high pJNK signature (HR inter, 0.27; 95% CI, 0.07–1; *p* inter = 0.05). Similar trends were noted for the other survival measures (Table S2). The observed relation is illustrated in the Kaplan-Meier curve provided in Figures S8 and S9.

Of note, we did not find differences at the expression levels of pJNK between high (>30%) and low TIL levels (Figure 1C). Taken together, these results suggest that JNK phosphorylation captured by the pJNK signature may influence the immune response in tumors with an inflamed phenotype.

### Gene expression differences characterizing inflamed tumors based on pJNK levels

To gain a deeper understanding of the underlying molecular and cellular mechanisms responsible for differences in survival among patients with inflamed tumors (IM and/or TILs >30%) based on pJNK levels, we examined the expression levels of multiple immune targets. We also interrogated several gene sets hallmarks.^22^

**Figure 2:**
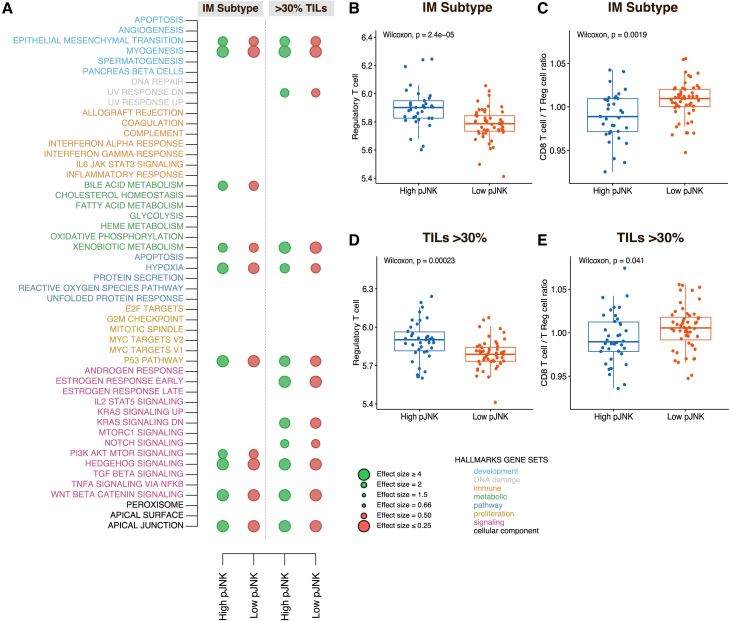
GSVA hallmark gene sets across pJNK levels and distribution of immune cells by pJNK levels (A) GSVA hallmarks gene sets in immune-related tumors (A). Effect size is obtained from linear regression models. *p* values are from the Wilcoxon rank-sum test (one group vs. others) and are adjusted according to FDR. Circles are shown if FDR<0.05. Positive associations are shown in green, while negative associations are shown in red. (B–E) Boxplot shows the distribution of Treg cell levels (B and D) and the ratio of CD8^+^ T cells to that of Treg cells (C and E) across low- and high pJNK levels in immune-related tumors. Boxplot elements: median; box limits, upper and lower quartiles, and whiskers (5^th^ and 95^th^ percentile). *p* value was determined using the Wilcoxon rank-sum test.

As depicted in Figure 2A, high pJNK levels were associated with an enrichment of genes related to epithelial-mesenchymal transition and myogenesis as well as hypoxia, p53 signaling, and various hallmarks related to signaling pathways including PI3K-AKT-mTOR.

Since the activation of the JNK pathway has been previously linked to the presence of Treg cells, while JNK inhibition has been shown to increase the ratio of CD8 T cells to Treg cells,^12^ we assessed the levels of Tregs and CD8^+^ T cells using CIBERSORT.^23^ Treg cells were significantly elevated in IM tumors with high pJNK levels (Figure 2B). Similarly, CD8^+^ T cells were also significantly higher in tumors with elevated pJNK levels (Figures S10A–S10D). However, the CD8^+^ T cell/Treg cell ratio was markedly higher in IM tumors with low pJNK levels (Figure 2C). Consistent trends were observed in tumors with high TIL levels (Figures 2D and 2E). In contrast, tumors with distinct phenotypes or low TIL levels also exhibited elevated Treg levels in association with high pJNK (Figures S10E and S10F). Notably, no differences were observed in the CD8^+^ T cell/Treg cell ratio in these cases, potentially underscoring the role of an inflammatory TME in shaping immune responses (Figures S10G and S10H).

We also evaluated the association of pJNK on the geographic localization of TILs. For this analysis, we classified our samples into three tumor immune microenvironment (TIME) categories reflecting TIL localization developed by Gruosso et al.^24^ namely fully inflamed (FI, *n* = 84), stroma restricted (SR, *n* = 103), and margin restricted (MR, *n* = 139) (Figure S4). According to the TIME classification, FI tumors predominantly correspond to the IM subtype. This subtype is associated with elevated expression of immune cell populations and immune-related targets, often referred to as “immune hot” tumors. In contrast, the stromal-restricted (SR) TIME subtype is primarily linked to the BL phenotype. Meanwhile, the MR TIME subtype encompasses the mesenchymal (M), LAR, and MSL phenotypes. These subtypes are typically considered “immune cold” due to their reduced immune cell presence and suppressed immune target expression.

**Figure 3:**
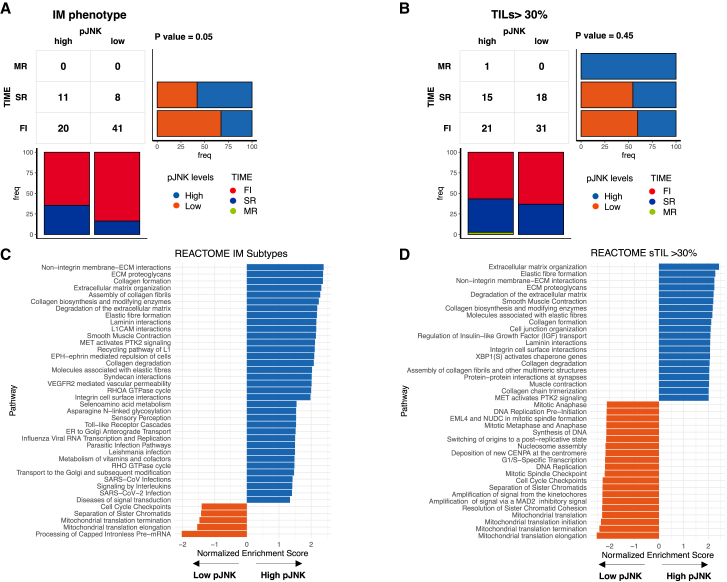
Associations between TIME subtypes and pJNK levels and enriched reactome pathways for pJNK subgroups (A and B) Associations between tumor immune microenvironment (TIME) subtypes and pJNK levels in tumors with an IM phenotype (A) and tumors with high TILs levels (B). *p* value was determined using Fisher’s exact test. (C and D) Top 20 Reactome pathways enriched for low and high pJNK subgroups in tumors with an IM phenotype (C) and tumors with high TILs (>30%) levels (D). Pathways are shown if FDR <0.05.

IM tumors with low levels of pJNK were associated with higher proportions of FI pattern. In contrast, the proportion of SR tumors was higher in the high pJNK group (Figure 3A). Similar patterns were found for high-TILs tumors. However, these were not significant (Figure 3B). These findings provide further evidence that inflamed tumors with low pJNK signature levels exhibit a more active immune response than those with high pJNK signature levels. When evaluating the TIME distribution across pJNK levels for tumors that do not have an IM subtype and tumors with low TILs (<30%), low pJNK levels were also associated with a higher proportion of SR tumors. However, for these tumors the predominant subtype was MR instead of FI as observed in immune-related tumors (Figure S11).

Finally, we performed differential gene expression (DGE) analysis between tumors with high and low pJNK levels. Gene set enrichment was performed using the Reactome gene sets.^25^ Overall, we observed that biological pathways related to extracellular matrix organization (e.g., collagen formation, proteoglycans, and syndecan interactions) were associated with high levels of pJNK in both IM tumors (Figure 3C) and tumors with high TILs (Figure 3D). On the other hand, functions related to cell cycling processes were enriched in immune-related tumors with low levels of pJNK (Figure 3C). These results suggest that high levels of pJNK are enriched for stroma-related processes, which are commonly associated with a more immunosuppressive TME.^26^ All results obtained from the enrichment analysis can be found in Data S1.

### pJNK levels and CM maintenance therapy

In the TNBC sub-cohort of the IBCSG 22-00 trial, we previously observed that patients with IM tumors received benefit from low-dose CM maintenance therapy. We hypothesized that this benefit could be attributed to the depletion of Treg cells in the TME through metronomic chemotherapy.^19^ In the following analysis, we aimed to investigate whether pJNK signature levels could further aid in identifying patients who may benefit from CM maintenance therapy, considering the association between the JNK pathway and Tregs in the TME (Figure 2C).

**Figure 4:**
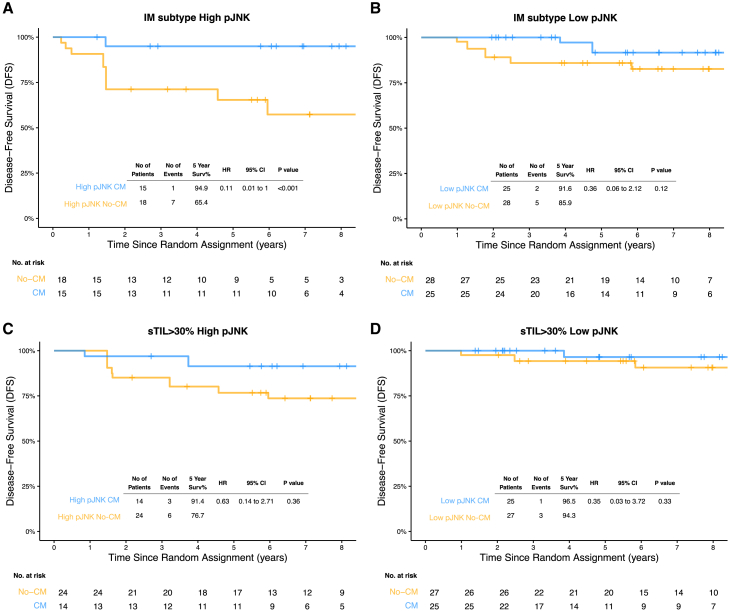
Kaplan-Meier estimates in IM phenotype and high TILs tumors stratified by JNK levels and CM treatment (A and B) Kaplan-Meier (KM) estimates of disease-free survival (DFS) for tumors with an immunomodulatory (IM) phenotype with high JNK levels (A) and with low JNK levels (B) in the context of CM treatment. (C and D) KM estimates for tumors with TILs >30% with high JNK levels (C) and with low JNK levels (D) according to CM treatment. *p* value represents the Cox proportional hazards model obtained with the likelihood ratio test. The CM group represents patients with metronomic treatment whereas no-CM group did not receive it.

Among patients with IM tumors with high pJNK levels, patients receiving CM maintenance therapy exhibited an estimated 5-year DFS rate of 94.9%, compared to 65.4% in the no-CM group (HR, 0.11; 95% CI, 0.01–1) (Figure 4A). Smaller differences were observed for IM tumor with low pJNK levels where the estimated 5-year DFS rate was 91.6% for the CM group compared to 85.9% of no-CM group (HR, 0.36; 95% CI, 0.06–2.12) (Figure 4B). Same effect was observed for OS, DRFI, and BCFI (Figures S12 and S13). These results suggest that IM tumors with high pJNK levels (immunosuppressive TME) receiving CM treatment have survival outcomes comparable to IM tumor with low pJNK (non-immunosuppressive TME). Furthermore, these differences were not observed for the other subtypes where Treg levels were lower for high pJNK levels (Table S3).

We observed similar clinical associations in tumors with high levels of TILs and high pJNK level, suggesting a better outcome with CM treatment (estimated 5-year DFS rate of 91.4% and 76.7% for CM maintenance and no-CM, respectively; HR, 0.63; 95% CI, 0.14–2.71) (Figures 4C and S14). For tumors with low pJNK levels, no differences were observed (5-year DFS rate was 96.5% for the CM group compared to 94.3 of no-CM group; HR, 0.35; 95% CI, 0.03–3.72) (Figures 4D and S15). Additionally, the differences in CM treatment outcomes between high and low pJNK levels were not seen in tumors exhibiting lower levels of TILs (Table S4). Although similar better outcomes with CM treatment were observed for both high pJNK levels in IM tumors and those with high TILs, the effect was significantly stronger in IM tumors.

These findings further support the notion that the benefit of CM in patients with pJNK-high immune-related tumors (IM and high TILs) may be linked to depletion of Treg cells.^19^

## Discussion

Understanding immunosuppressive pathways in the TME has gained significant relevance in recent years, especially with the advent of immunotherapy in TNBC.^18^ Additionally, growing interest is in identifying the optimal treatment companion for ICIs to maximize their effectiveness.

The objective of this study was to gain a better understanding of the immunological mechanisms underlying the pJNK pathway within the TME in different molecular subtypes of TNBC. Furthermore, we explored how pJNK interacts with immunosuppressive pathways in the context of low-dose CM maintenance therapy. A deeper comprehension of the IM effects associated with metronomic chemotherapy could be crucial in developing new therapeutic strategies for early-stage TNBC.

In this study, using a TNBC cohort from the randomized phase 3 IBCSG 22-00 trial,^27^ we demonstrated the heterogeneity of immune TME in immune-related TNBCs through the pJNK pathway. These findings support the hypothesis that considering TNBC heterogeneity in assessing and developing treatment strategies may be crucial.

Since RPPA data were unavailable in our current study, we developed an RNA-seq signature that can estimate phosphorylation levels of the pJNK pathway using TCGA dataset. Our results indicate that the pJNK gene signature is associated with the same biological processes observed with JNK phosphorylation levels,^12^ suggesting that the signature reliably represents this phenotype.

The immune-enriched IM subtype has previously been associated with high expression of immune targets and adaptive immune-related cell populations, indicating an “immune hot” TME. Conversely, the M subtype is considered “immune cold” with low expression of immune cells and downregulation of most immune targets. LAR and MSL tumors have also been linked to an immunosuppressive and pro-tumorigenic phenotype, characterized by high expression of stromal signatures.^6^ We found significant associations between pJNK expression levels and the LAR and MSL molecular subtypes, suggesting a potential connection between the pJNK pathway and stromal processes.

While no survival differences were observed between high and low pJNK levels in the overall RNA-sequencing population, our results revealed a relation of the pJNK pathway without outcome in inflamed tumors (IM or high TILs) that was not evident for non-inflamed tumors. Specifically, patients with tumors expressing an immune-enriched phenotype and low pJNK levels had a trend of better outcomes than those with high pJNK levels. Similarly, tumors with TILs higher than 30% and low pJNK levels exhibited the same outcome. These findings suggest that the pJNK signature can identify subgroups within inflamed tumors where immunosuppressive processes are predominant. While our analysis focused on molecular subtypes, an alternative approach using a continuous “IM score” across all TNBC subtypes could provide further insights into how pJNK expression correlates with immune infiltration. Future studies integrating such an analysis may help clarify the role of pJNK in shaping the immune landscape beyond categorical subtype classifications.

To gain further insights into the biological pathways associated with high or low pJNK activation in immune-related tumors, we conducted in-depth gene expression profiling. These analyses revealed that high pJNK levels were enriched with stromal processes such as ECM (extracellular matrix) synthesis and collagen formation, accordant with a line of evidence suggesting that activated JNK signaling in fibroblasts promotes fibrosis in a wide range of organs, including breast tissue.^28^^,^^29^^,^^30^^,^^31^ Cancer-associated fibroblasts (CAFs) have been recognized to play crucial roles in the immune evasion,^32^ promoting immunotherapy resistance and leading to poor outcomes in TNBC^33^ by expressing immune checkpoint molecules and modulating a variety of cytokines.^34^ Although limited, evidence suggests that JNK signaling is also upregulated in breast cancer stroma,^35^ and active JNK signaling regulates chemokine production in CAFs, which leads to suppressing the infiltration of cytotoxic T lymphocytes.^31^ Therefore, JNK signaling in CAFs warrants further research and should be a potential therapeutic target.

As previously demonstrated, the pJNK pathway influences the presence of Tregs and CD8^+^ T cells in TNBC, with high levels of phosphorylated JNK associated with increased Tregs infiltration. In contrast, inhibition of pJNK signaling increases CD8^+^ T cells while decreasing Tregs.^12^ Consistent with these results, we observed that high pJNK signature levels were associated with significantly higher Treg levels and lower CD8^+^ T cell levels, further validating the reliability of the pJNK gene signature in representing JNK phosphorylation.

In early-stage TNBC, neoadjuvant immunotherapy combined with chemotherapy is increasingly used. Following the positive results of the KEYNOTE-522 trial, ICIs are now approved and included in the standard of care for patients with stage 2 to 3 early TNBC.^18^ Based on our findings, we hypothesize that inhibiting JNK, thereby making the TME more immune active, could enhance the effect of ICIs and decrease the need for chemotherapy. Indeed, the JNK inhibitor BMS-98360 is currently being evaluated as monotherapy and in combination with chemotherapy and nivolumab in patients with advanced solid tumors, including TNBC (NCT05625412).

The IBSCG 22-00 trial randomized patients to receive one year of low-dose maintenance CM after adjuvant chemotherapy, based on previous findings suggesting a possible antiangiogenic effect of metronomic chemotherapy.^32^^,^^33^ This provides a potential active treatment option with a favorable toxicity profile.^27^ In the IBSCG 22-00 trial, inflamed TNBCs showed better outcomes when receiving metronomic CM maintenance.^19^ Previous analyses of this cohort identified a benefit of low-dose CM associated with regulatory T cells. These findings, together with evidence from other studies, suggest that the potential benefit of low-dose CM may be related to its immune-modulating effects, possibly through the depletion of regulatory T cells, thereby enhancing anti-tumor immunity.^7^^,^^36^ Such IM effects of metronomic chemotherapy make it a promising partner for ICIs, such as PD-1/PD-L1 and CTLA-4 inhibitors. By enhancing the tumor’s immunogenicity and reducing immune suppression, metronomic CM can potentiate the efficacy of these immunotherapies.

Considering the known association between pJNK and Tregs, we evaluated the association of CM treatment across different molecular subtypes stratified by pJNK signature levels. We found that IM tumors with high pJNK levels had better outcomes when treated with low-dose CM than other subtypes. These results further support the potential immune-modulating effect of low-dose CM chemotherapy, as previously described in several studies.^36^^,^^37^^,^^38^^,^^39^ Furthermore, the JNK signature may identify the population that they will benefit from metronomic CM.

We acknowledge that our study has some limitations including the small sample size use for some analysis and its retrospective nature. However, the analysis was intended to be exploratory, and the findings warrant future validation in larger, prospective studies. In the future, with more immunotherapy trials available, a more comprehensive validation of our findings should be performed within the context of studies evaluating ICIs, enabling the study of the association between JNK pathway activation and immunotherapy resistance.

In conclusion, we have developed a gene expression signature capable of estimating the phosphorylation level of the JNK protein. Our results highlight the heterogeneity within inflamed TNBCs regarding immune-activating and suppressive balance, as captured by the pJNK gene signature. High levels of pJNK in immune-related tumors are associated with worse clinical outcomes, suggesting the potential of the pJNK signature to identify immunosuppressive tumors. Furthermore, we observed a benefit from low-dose CM in immune-related tumors with high pJNK levels. These findings suggest a potential role for the pJNK signature as a biomarker for immunotherapy in TNBC and warrant further validation.

### Limitations of the study

Despite the promising insights gained from our analyses, several limitations should be acknowledged. The retrospective nature of the study and the limited sample size for specific subgroup analyses may reduce the generalizability of our findings. Additionally, although the RNA-seq-based pJNK signature was designed to reflect the biological activity of the pathway and showed concordance with known immunological processes, its correlation with RPPA-derived phosphorylation levels was modest. While the signature was externally validated, the number of samples in the validation cohort was limited, and the lack of additional publicly available datasets restricted further validation efforts. Furthermore, the study cohort was derived from an adjuvant clinical trial in which all patients received chemotherapy, which may limit the applicability of our results in current treatment settings involving ICIs. As such, prospective validation in immunotherapy-specific trials is essential to confirm the predictive value of the pJNK signature and its potential role as a biomarker for tailoring treatment strategies in TNBC.

## Resource availability

### Lead contact

Further information and requests for resources and reagents should be directed to and will be fulfilled by the lead contact, Christos Sotiriou (christos.sotiriou@hubruxelles.be).

### Materials availability

This study utilized clinical trial data as the primary research material. Details on how to access these data are provided in the data and code availability section.

### Data and code availability

• The IBCSG 22-00 trial is registered at www.clinicaltrials.gov as NCT00022516.
• After publication, access to deidentified participant data may be requested by researchers by submitting a proposal (to stat_center@ibcsg.org), which will be reviewed for scientific merit and feasibility in accordance with the Guidelines for Collaborative research (https://www.ibcsg.org/images/Member/Publi/Documents/Guidelines_for_Collaborative_Research_for_ETOP_IBCSG_Partners_Foundation_Dec_2022.pdf) and data sharing policy (https://www.ibcsg.org/images/Member/Publi/Documents/Data_Sharing_Policy_for_IBCSG_Trials_Dec_2022.pdf) for IBCSG trials.
• Code to perform this classification is available at https://github.com/BCTL-Bordet.
• Any additional information required to reanalyze the data reported in this paper is available from the lead contact upon request.

## Acknowledgments

A.J.G. is supported by the Télévie and the Fonds National de la Recherche Scientifique (F.R.S.-FNRS) and by the Fondation Rose et Jean Hoguet. M.R. was supported by the Télévie and the Fonds National de la Recherche Scientifique (F.R.S.-FNRS) , by the 10.13039/501100008598Fondation Rose et Jean Hoguet and by an AIRC fellowship for Italy. L.B. is supported by the Belgian “10.13039/501100005026Fondation Contre le Cancer”. D.V. is supported by a grant of the Région Wallonne under the WALInnov program.

We thank the International Breast Cancer Study Group, a division of ETOP IBCSG Partners Foundation, for sharing data and biospecimens from the IBCSG 22-00 trial. IBCSG is indebted to the patients who participated in the IBCSG 22-00 trial and their families, the IBCSG 22-00 investigators at all clinical centers and their teams, pathologists and biobanks who provided the FFPE tumor tissue for IBCSG 22-00, the IBCSG Translational Research Working Group, and the IBCSG Central Pathology Office. We thank the Fonds National de la Recherche Scientifique (F.R.S.-FNRS), the 10.13039/100001006Breast Cancer Research Foundation (BCRF), the Association Jules Bordet, and National Institutes of Health (NIH) grant (1R01CA278056-01A1) awarded to Naoto T. Ueno for supporting the study.

## Author contributions

Conceptualization, A.J.G., T.S., M.R., D.J.M., X.X., X.W., D.R.R., D.V., L.B., S.M., R.K., M.C., S.L., G.V., M.M.R., F.R., C.S., and N.T.U.; methodology, A.J.G., T.S., M.R., D.J.M., X.X., X.W., D.R.R., D.V., L.B., S.M., R.K., M.C., S.L., G.V., M.M.R., F.R., C.S., and N.T.U.; software, A.J.G., D.V., M.R., and D.J.M.; validation, A.J.G., T.S., M.R., D.J.M., X.X., X.W., D.R.R., D.V., L.B., S.M., R.K., M.C., S.L., G.V., M.M.R., F.R., C.S., and N.T.U.; formal analysis, A.J.G., T.S., M.R., and D.J.M.; investigation, A.J.G., T.S., M.R., D.J.M., X.X., X.W., D.R.R., D.V., L.B., S.M., R.K., M.C., S.L., G.V., M.M.R., F.R., C.S., and N.T.U.; resources, M.C., G.V., M.M.R., S.M., C.S., and N.T.U.; data curation, M.M.R., D.V., A.J.G., and M.R.; writing – original draft, A.J.G., T.S., M.R., and D.J.M.; writing – review & editing, A.J.G., T.S., M.R., D.J.M., X.X., X.W., D.R.R., D.V., L.B., S.M., R.K., M.C., S.L., G.V., M.M.R., F.R., C.S., and N.T.U.; visualization, A.J.G. and T.S.; supervision, F.R., D.V., C.S., N.T.U., M.R., X.X., and X.W.; project administration, R.K., F.R., C.S., and N.T.U.; funding acquisition, C.S. and N.T.U.

## Declaration of interests

N.T.U. has consulting roles with the following companies: AstraZeneca plc, Bayer AG, Pfizer Inc., Gilead Sciences, Inc., Chugai Pharmaceutical Co., CytoDyn Inc., Daiichi Sankyo, Inc., DynaMed, LLC, Eisai Co., Ltd., KeChow Pharma, Inc., Lavender Health Ltd., OBI Pharma Inc., OncoCyte Co., Ourotech, Inc., DBA Pear Bio, Kirilys Therapeutics, Inc., Peptilogics, Inc., Phoenix Molecular Designs, Preferred Medicine, Inc., Puma Biotechnology, Inc., Sumitomo Dainippon Pharma, Inc., Sysmex Co. Ltd., Takeda Pharmaceuticals, Ltd., Unitech Medical, Inc., CARNA Biosciences, Inc., ChemDiv, Inc., DualityBio, LARVOL, Oncolys BioPharma Inc., Rakuten Medical, Inc., Merck Co., AnHeart Therapeutics Inc., Carisma Therapeutics, Inc., Lilly, Inc. and Therimunex. He has speaker or preceptorship roles with the following companies: Bristol-Myers Squibb, CareNet, Inc., Chugai Pharmaceutical Co., Genomic Health, Kyowa Hakko Kirin Co., Ltd., Sumitomo Dainippon Pharma, Inc., and Medscape. He has research agreements with the following companies: AnHeart Therapeutics Inc., Eisai Co., Ltd., Gilead Sciences, Inc., Phoenix Molecular Designs, Daiichi Sankyo, Inc., Puma Biotechnology, Inc., Merck Co., Oncolys BioPharma Inc., OBI Pharma Inc., ChemDiv, Inc., Tolero Pharmaceuticals, Inc., and VITRAC Therapeutics, LLC.

C.S. is in the advisory board (receipt of honoraria or consultations fees) of Astellas, Cepheid, Vertex, Seattle genetics, Puma, Amgen, and Exact Sciences. He has participated in company sponsored speaker’s bureau from Eisai, Prime Oncology, Teva, Foundation Medicine, and Exact Sciences. He has received travel grants from Roche, Genentech, and Pfizer.

M.C. is Co-Chair Scientific Committee IBCSG and has received a research grant from Roche.

S.L. receives research funding to her institution from Novartis, Bristol-Myers Squibb, Merck, Puma Biotechnology, Eli Lilly, Nektar Therapeutics, AstraZeneca, Roche-Genentech, and Seattle Genetics. She has acted as consultant (not compensated) to Seattle Genetics, Novartis, Bristol-Myers Squibb, Merck, AstraZeneca, Eli Lilly, Pfizer, and Gilead Therapeutics and Roche-Genentech. She has acted as consultant (paid to her institution) to Aduro Biotech, Novartis, GlaxoSmithKline, Roche-Genentech, Astra Zeneca, Silverback Therapeutics, G1 Therapeutics, PUMA Biotechnology, Pfizer, Gilead Therapeutics, Seattle Genetics, Daiichi Sankyo, Merck, Amunix, Tallac Therapeutics, and Eli Lilly and Bristol Meyers Squibb.

L.B. is in the advisory board of iTEOS Therapeutics and Domain Therapeutics. She is investigator-initiated trial (funds paid to institution).

## STAR★Methods

### Key resources table

**Table:** 

REAGENT or RESOURCE	SOURCE	IDENTIFIER
**Biological samples**		

FFPE tumor surgical samples from human	IBCSG 22-00 trial	www.clinicaltrials.gov (NCT00022516)

**Deposited data**		

RNA-seq data	IBCSG 22-00 trial	Access to data available upon request to stat_center@ibcsg.org
TCGA breast tumor samples	The Cancer Genome Atlas (TCGA), NCI/NIH	https://portal.gdc.cancer.gov/

**Software and algorithms**		

R software (version 4.1.0)	the R Core Team and the R Foundation for Statistical Computing	https://www.r-project.org/
Trimmomatic v0.39	Bolger et al.^44^	http://www.usadellab.org/cms/?page=trimmomatic
STAR v2.7.3a (aligned to ENSEMBL v98)	Dobin et al.^40^	https://github.com/alexdobin/STAR
ENSEMBL v98 (genome annotation)	ENSEMBL	https://www.ensembl.org/info/data/ftp/index.html
Salmon v1.3.0	Patro et al.^41^	https://salmon.readthedocs.io
DESeq2	R software	https://bioconductor.org/packages/release/bioc/html/DESeq2.html
GSVA	R software	https://bioconductor.org/packages/release/bioc/html/GSVA.html
MSigDB (Molecular Signatures Database)	R software	https://www.gsea-msigdb.org/gsea/msigdb
fgsea	R software	https://bioconductor.org/packages/release/bioc/html/fgsea.html
code	Github	https://github.com/BCTL-Bordet

### Experimental model and study participant details

#### IBSCG 22-00 clinical trial and patient selection

The adjuvant clinical trial IBCSG 22-00 results have previously been published.^27^ In IBCSG 22-00, patients with ER/PR-negative tumors (determined locally) who had standard adjuvant chemotherapy were randomized to either observation or maintenance CM (cyclophosphamide 50 mg/day orally and methotrexate 2.5 mg/twice a day orally) for one year. The cohort selected for this study is a subset of 647 patients with TNBC. TNBC was defined as ER/PR<1% immunohistochemistry score and absence of HER2 overexpression or amplification determined by the central pathologic evaluation.^40^ In addition, stromal tumor-infiltrating lymphocyte (TIL) levels were also evaluated in this cohort. For the selection of the 498 patient TNBC cohort for RNA sequencing, a case-cohort-like sampling following a ratio of 1 (cases) to 3 (non-cases) was used^41^ (Figure S1).

Survival endpoints of the IBCSG 22-00 trial included the breast cancer-free interval (BCFI), defined as the time from randomization to the recurrence of invasive breast cancer (local, regional, or distant) or invasive contralateral breast cancer; disease-free survival (DFS), defined as the time from randomization to the first appearance of invasive recurrence of breast cancer (local, regional, or distant), invasive contralateral breast cancer, second (non-breast) invasive cancer or death without prior cancer event; distant recurrence-free interval (DRFI), defined as the time from randomization to the recurrence of breast cancer at a distant site; and overall survival (OS), defined as the time from randomization to death from any cause.

The IBCSG 22-00 trial is registered at www.clinicaltrials.gov as NCT00022516. The IBCSG 22-00 trial received approval from the ethics committee and relevant health authorities at each participating site and was conducted in accordance with the Declaration of Helsinki. Furthermore, all patients involved in the trial provided written informed consent, which also encompassed future biomarker research. The IBCSG Translational Research Working Group approved the current analysis.

### Method details

#### Sampling design

RNA sequencing was performed on a selection of 498 patients from the TNBC cohort, using a case-cohort-like sampling following a ratio of approximately 1 (cases) to 3 (non-cases) (24). All patients who presented a recurrence event defined by breast cancer-free interval (BCFI) were included in the sub-cohort. The randomization of the samples selected the non-cases according to 4 stratification factors: Tumor size (T1 [0-2cm], T2 [>2-5cm], or T3 [>5cm]), Nodal status (N0, N+ 1–3, N+ ≥4), Age (<40, 40-<50, 50-<60, 60+) and Treatment (CM maintenance or no-CM).

#### Samples acquisition

RNA was extracted from tumor samples obtained during surgery from archival formalin-fixed paraffin-embedded (FFPE) tissues. The flow diagram (Figure S1) shows the patients’ flow from the TNBC TILs cohort (23) to the TNBC sub-cohort used for these analyses.

#### RNA sequencing and expression quantification

Using the AllPrep FFPE RNA kit, 500 ng of RNA was extracted from the FFPE samples. Samples with cellularity below 15% were excluded. The RNA material, once sequenced using the Ribo Zero sequencing kit (Illumina), was subsequently employed for gene expression analysis.

High-quality reads were selected using Trimmomatic v0.39^42^ and subsequently aligned to the human reference GRCh38/hg38 genome using STAR.^43^ Salmon^44^ was used to estimate transcript abundance based on STAR alignments (ENSEMBL v98).

Samples derived from FFPE tissue often exhibit lower read counts, a circumstance attributed to the intrinsic limitations of the tissue type and the potential for RNA to degrade over time. A threshold was set at less than 200,000 reads mapped to the transcriptome; samples falling below this threshold were subsequently excluded from analysis. This filtering resulted in the exclusion of 151 samples, yielding a final cohort comprising 347 patients (Figure S1). Furthermore, only genes that were expressed in at least 70% of the samples were selected for study. A deep analysis based on the quality of the samples was previously conducted at.^40^

The gene expression data were then normalized using the variance stabilizing transformation (VST) method from the R package DESeq2.^45^

The distribution of clinicopathologic characteristics in Table S5 shows that the hazard ratios derived from the case-cohort sample closely align with those from the 647-patient TNBC TILs cohort. Furthermore, these patient characteristics were well-balanced across treatment arms in the RNA-seq weighted cohort, as detailed in Table S6.

#### JNK signature

TCGA data were downloaded using the TCGA data portal (https://portal.gdc.cancer.gov/) from the Pan-Cancer Atlas release (April 2018). Samples from patients with TNBC with both RNAseq gene expression data and phospho-JNK targeted proteomics were randomly divided into a training dataset (*N* = 95) and testing dataset (*N* = 46). The RNAseq dataset was log-transformed and further filtered for genes present in 3 datasets (i.e., TCGA, METABRIC and TNBC cohort of IBCSG 22-00), resulting in a total of 8946 genes for further analysis. For training, we performed stochastic subsampling of the training dataset, selecting one-third of the samples to determine the Pearson correlation coefficient between phospho-JNK and gene expression levels. After repeating this process 1,000 times, we selected genes with a correlation coefficient with an absolute value greater than 0.25 at least 75% of the time as a set of candidate markers. Elastic net regression was performed using the function lassoglm() with α = 0.5 and 7-fold cross-validation to determine the final transcriptional signature to reflect JNK phosphorylation. The reserved testing dataset validated the final phospho-JNK gene expression signature using Pearson correlation. The signature was calculated as the mean product of the signature coefficients and log-transformed z-normalized expression values for each sample. All analyses were performed in MATLAB R2019a.

#### TNBC molecular classification

Lehmann’s molecular subtypes were determined by using the list of genes positively and negatively associated with each subtype, as published in the original paper.^1^ Signatures for each subtype were determined by calculating the difference of the mean values for the genes positively and negatively associated after centering and scaling each gene. Subsequently, for every sample, the TNBC molecular subtype with the highest score was assigned. The main difference from the published method is the absence of the unstable subtype, as each sample was associated with a specific subtype.

Samples with basal-like 2 (BL2) phenotypes were reassigned using the second-highest score by Bareche et al.^3^ This reassignment led to the identification of five more stable TNBC subtypes, namely basal-like (BL), immunomodulatory (IM), mesenchymal (M), luminal androgen receptor (LAR), and mesenchymal stem-like (MSL).

#### Tumor immune microenvironment molecular subtypes

The TIME (Tumor Immune Microenvironment) subtypes characterize the spatial distribution of CD8^+^ TIL through three distinct patterns: full inflamed (FI), stroma restricted (SR), and margin restricted (MR)/immune desert (ID).^24^ FI tumors present high infiltrating levels of CD8 T cells, whereas MR tumors are characterized by a low presence of CD8 T cells primarily located in the tumor’s margins. SR tumors exhibit some areas with CD8 T cell populations with stromal cells predominant in the TME.

These TIME subtypes were derived from gene expression data as described in Gruosso et al.^24^ Samples were classified into 4 categories: FI, SR and MR, while samples not satisfying the classification criteria were left unclassified.

#### Gene signatures

GSVA was performed using the GSVA R package (version) on the hallmarks gene set from MSigDB obtained with the msigdb R package (version). The VST normalized data of the interest group was used as input to the GSVA package. CIBERSORT Tregs and CD8 T-cells were obtained from http://cibersortx.stanford.edu, and each signature was calculated as the median expression of the genes.

#### Differential expression analysis and reactome pathways

DESeq2^45^ was used to identify gene sets highly and lowly expressed in the low- and high-JNK immune-related tumors. DESeq2 model was adjust for the clinicopathological characteristics of age (≤40 years vs. > 40 years), tumor size (≤2cm vs. > 2cm), nodal status (N0 vs. N+), grade (I/II/NA vs. III) and adjuvant treatment (Anthracycline and No Anthracycline). Differential expression was performed on the gene raw counts data obtained as described above. After generating a ranked list of genes based on the stat column of the DESeq2 results, gene set enrichment analysis was performed using Reactome database^25^ with the fgsea R package (version 1.18.0). The significant enrichment pathways were selected by FDR <0.05.

#### Immune-related tumors

Immune-related tumors were defined according to immunomodulatory (IM) TNBC molecular subtype, or tumor-infiltrating lymphocytes (TILs) levels higher than 30%.^46^

### Quantification and statistical analysis

To address the potential bias arising from over-sampling recurrence BCFI events in the case-cohort-like sampling design, weighted analysis methods were employed in all analyses. Specifically, the generalized Horvitz-Thompson weighted method (inverse probability weighting) was utilized to adjust for this sampling imbalance.^41^ Weighted values were calculated by taking the inverse of the proportion of patients sampled within each sampling stratum from the TNBC TILs cohort, separately for recurrences and non-recurrences.

pJNK across the different TNBC subtypes and TILs levels were evaluated through a pairwise Wilcoxon rank-sum test (one group vs. others). Survival curves for OS, DFS, DRFI, and BCFI were generated using Kaplan-Meier weighted estimations across the various interest groups. *p* values were obtained using the log-rank test.

Multivariable survival analyses were performed with Cox proportional hazard regression model, and weighted interaction tests, adjusting for the clinicopathological characteristics of tumor size (≤2cm vs.>2cm), nodal status (N0 vs. N+), grade (I/II/NA vs. III) and adjuvant treatment (Anthracycline and No Anthracycline). *p* values in the Cox model were derived using the log likelihood between a model with the covariates and a model with the covariates plus the feature of interest. P interactions (p inter) were obtained with the Wald test. All survival analyses comparing the high and low JNK TNBC subtypes were conducted by comparing one group against the rest.

Logistic regression models were employed to evaluate the association of hallmarks of high and low JNK immune-related tumors. The analysis aimed to estimate the effect size, which was represented by odds ratios. *p* values were obtained using the Wilcoxon rank-sum test, comparing one group against the others. Fisher’s exact test evaluated the distribution of TIME subtypes across low and high-pJNK levels in immune-related tumors.

False discovery rates (FDR) were calculated using the Benjamin & Hochberg method whenever applicable to account for multiple testing. This correction was performed for the P interactions, hallmarks logistic regression model and GSEA. *p* values and FDRs below 0.05 were considered statistically significant. All tests were two-sided. All statistical analyses were performed using R (version 4.1.0).^47^

### Additional resources

The IBCSG 22-00 trial is registered at www.clinicaltrials.gov as NCT00022516.

## Supplemental information

Document S1. Figures S1–S15 and Table S1–S4

Data S1. Enrichment analysis results, related to Figure 3

Data S2. IBCSG 22-00 clinical trial information
