# Supplementary material for: Role of immunosuppressive JNK pathway in the tumor microenvironment among TNBC subtypes in IBCSG trial 22-00
Source: iScience. 2025 Jun 20;28(8):112964. doi: 10.1016/j.isci.2025.112964 (PMC12355117; doi:10.1016/j.isci.2025.112964)
Supplement: Document S1. Figures S1–S15 and Table S1–S4 [file mmc1.pdf]

iScience, Volume *28*

Supplemental information

Role of immunosuppressive JNK pathway in the tumor microenvironment among TNBC subtypes in IBCSG trial 22-00

Andrea Joaquin Garcia, Takashi Semba, Mattia Rediti, Daniel J. McGrail, Xuemei Xie, Xiaoping Wang, Dileep R. Rampa, David Venet, Laurence Buisseret, Samira Majjaj, Roswitha Kammler, Marco Colleoni, Sherene Loi, Giuseppe Viale, Meredith M. Regan, Françoise Rothé, Christos Sotiriou, and Naoto T. Ueno

FIGURE S1. CONSORT flow diagram, Related to STAR Methods:
Flow diagram shows the obtention procedure of the final TNBC RNA-seq cohort of 347 patients, 165 assigned to CM-maintenance and 182 to no further chemotherapy (no-CM).

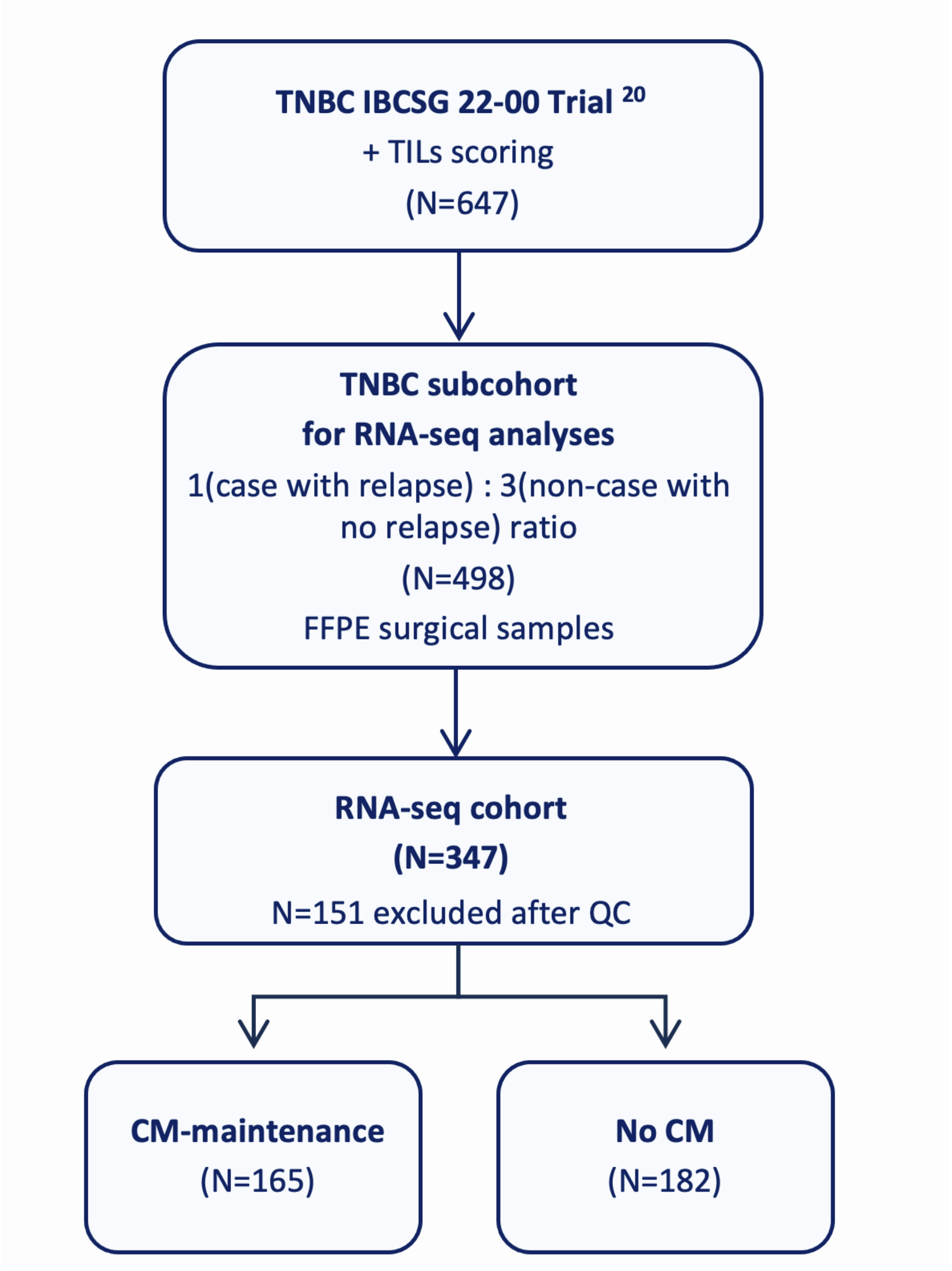

FIGURE S2. JNK signature, Related to STAR Methods**:**
Scatterplots showing Pearson's correlation coefficient r of expression levels for phospho-JNK and the score of pJNK gene signature in the training dataset **(A)** and the testing dataset **(B)** from the TCGA TNBC cohort and (C) CPTAC TNBC cohort.

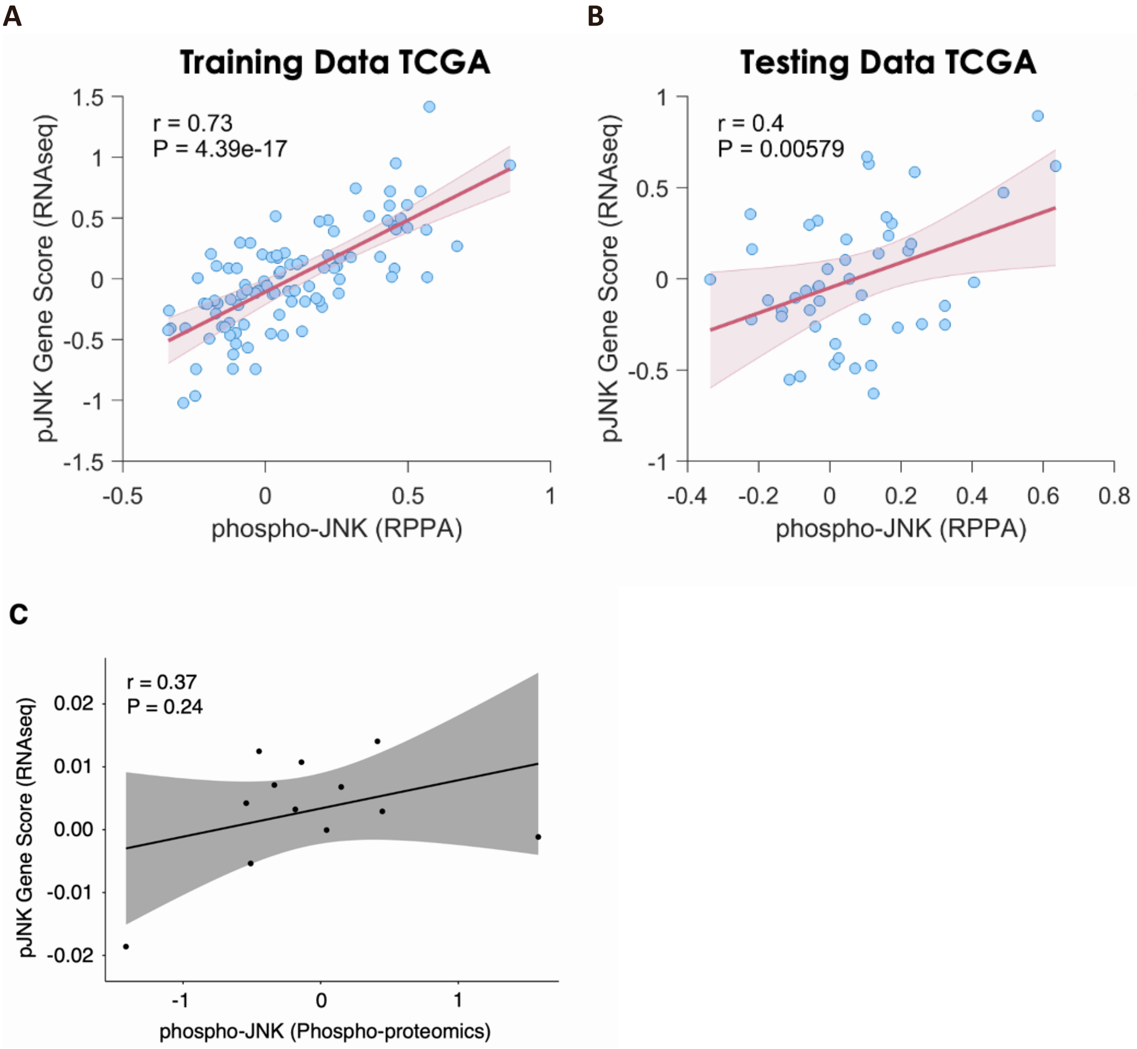

FIGURE S3. Kaplan-Meier pJNK levels, Related to Figure 1**:**

Kaplan-Meier estimates of overall survival (OS) **(A)**, Distant Recurrence-Free Interval (DRFI) **(B)** and Breast Cancer-Free Interval (BCFI) **(C)** according to low and high pJNK levels in all the patients. P value obtained from Cox proportional hazards model likelihood ratio test.

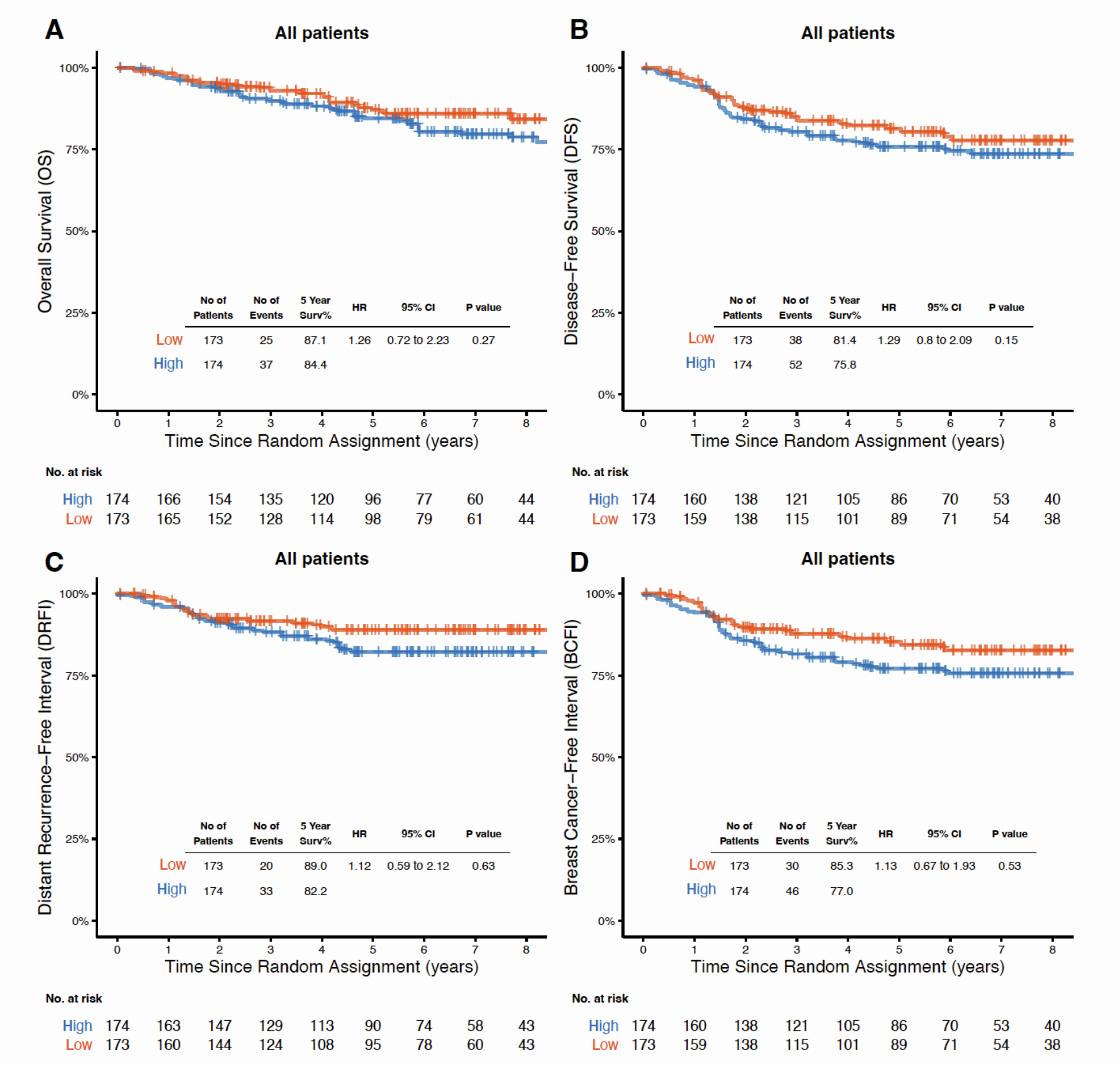

FIGURE S4. Alluvial diagram, Related to Figure 1 and Figure 3**:**
Alluvial diagram showing the flow of the tumors classified by TNBC molecular subtypes from Bareche classification (left node) to the TIME classification (right node). The color code is defined by the Bareche classification (left node).

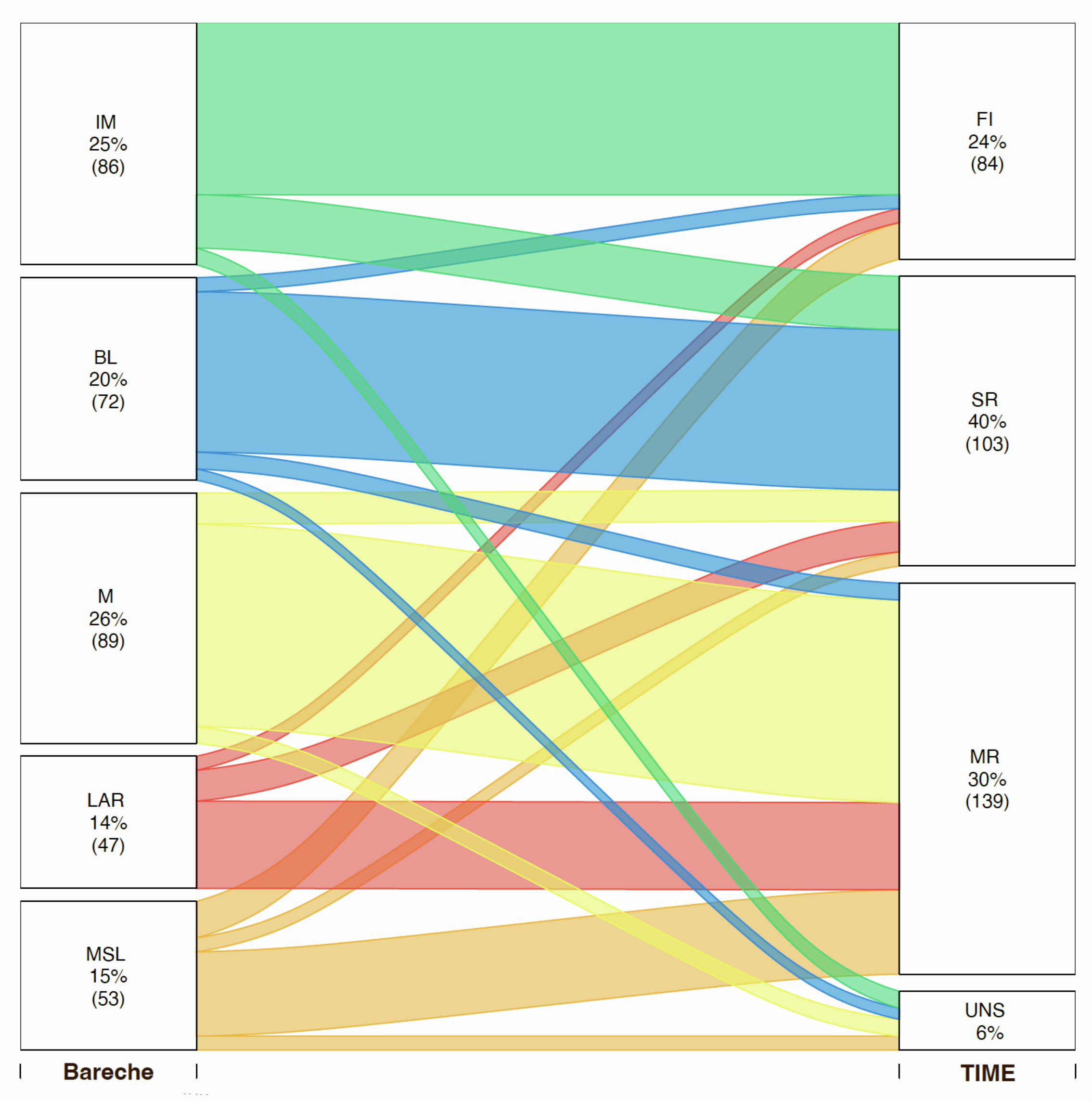

FIGURE S5. Cox model pJNK levels – TNBC subtypes, Related to Figure 1:
Results of the Cox proportional-hazard model for effect of pJNK levels on overall survival (OS) **(A)**, Distant Recurrence-Free Interval (DRFI) **(B)** and Breast Cancer-Free Interval (BCFI) **(C),** respectively, according to TNBC molecular subtypes. The HR inter is defined as the ratio of HR between subgroups with low (blue dots) and high (yellow dots) levels of pJNK. The p inter represents a Wald test that evaluates the effect of treatment by variable interaction. Low pJNK group represents samples with expression of the gene signature lower than the median, whereas the high pJNK group includes samples with expression higher than the median.

A

OS

	HR inter	CI inter	*p* inter	FDR
BL	1.4	0.35 to 5.7	0.63	0.99
IM	0.18	0.032 to 1	0.051	0.24
M	0.99	0.3 to 3.3	0.99	0.99
LAR	1.2	0.27 to 5.1	0.83	0.99
MSL	4	0.78 to 20	0.098	0.24

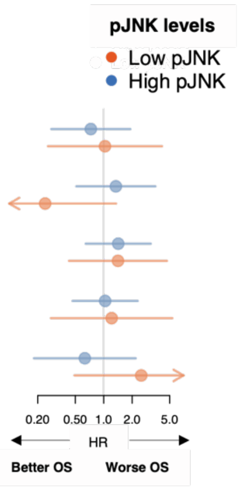

B

DRFI

	HR inter	CI inter	*p* inter	FDR
BL	1.8	0.39 to 8	0.47	0.66
IM	0.14	0.022 to 0.86	0.034	0.17
M	0.8	0.19 to 3.3	0.76	0.76
LAR	1.6	0.35 to 7.6	0.53	0.66
MSL	4	0.69 to 23	0.12	0.3

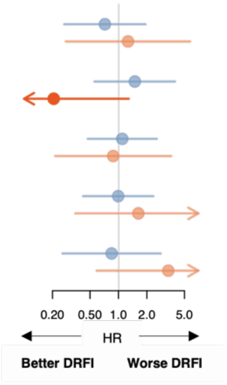

C

BCFI

	HR inter	CI inter	*p* inter	FDR
BL	2.1	0.57 to 8	0.26	0.54
IM	0.35	0.085 to 1.5	0.15	0.54
M	1	0.33 to 3.1	0.99	0.99
LAR	1.1	0.27 to 4.8	0.87	0.99
MSL	2.2	0.47 to 10	0.32	0.54

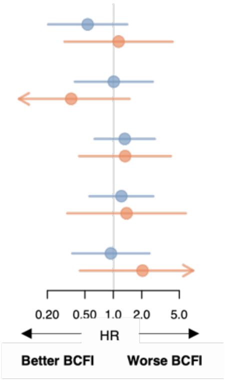

FIGURE S6. Kaplan-Meier IM subtype – pJNK levels, Related to Figure 1**:**

Kaplan-Meier estimates of Overall Survival (OS) **(A)**, Disease Free Survival (DFS) (**B**), Distant Recurrence-Free Interval (DRFI) **(C)** and Breast Cancer-Free Interval (BCFI) **(D)** according to low and high pJNK levels in tumors with an immunomodulatory (IM) phenotype.

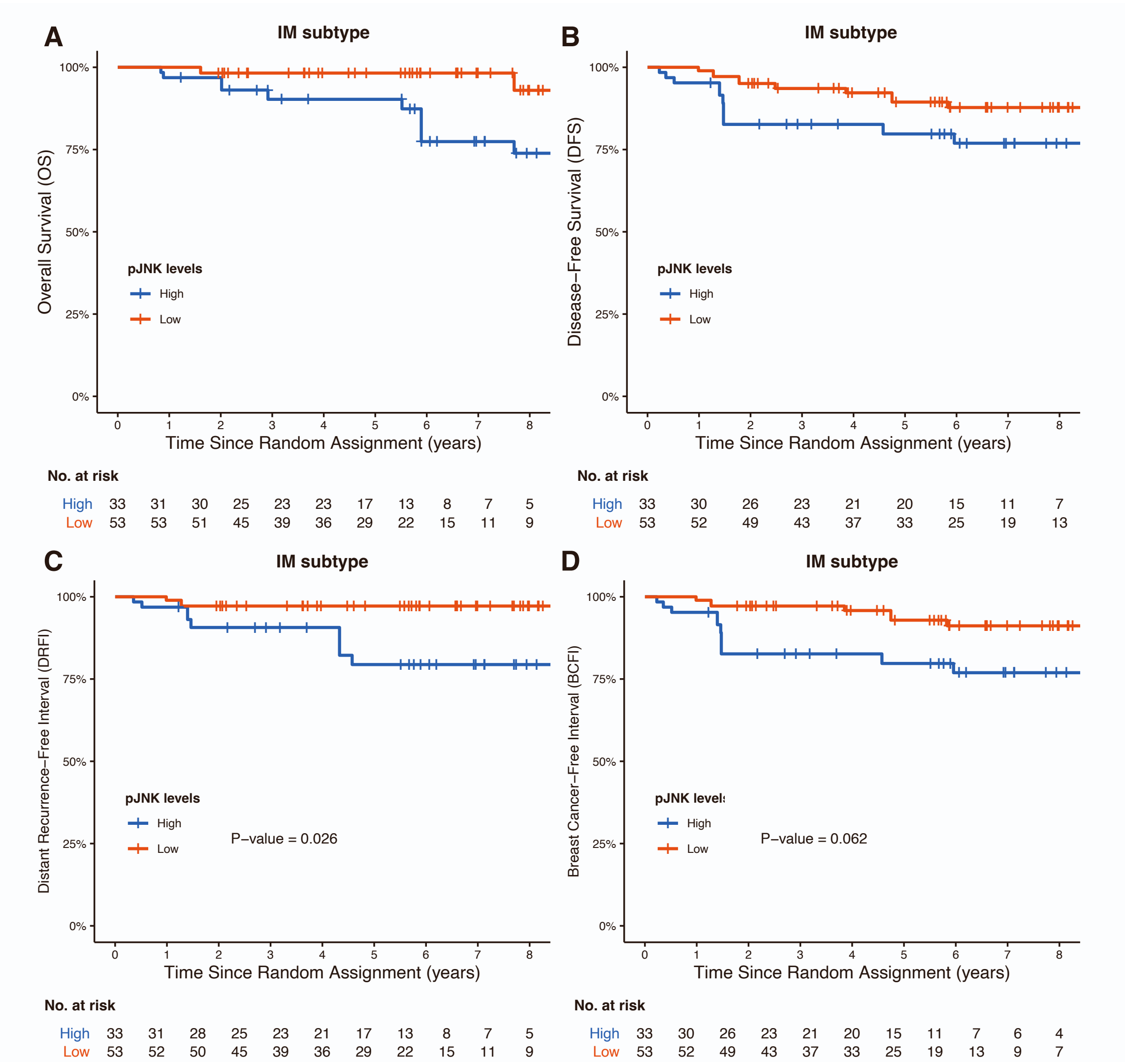

FIGURE S7. Kaplan-Meier other subtypes– pJNK levels, Related to Figure 1**:**
Kaplan-Meier estimates of Overall Survival (OS) **(A)**, Disease Free Survival (DFS) (**B**), Distant Recurrence-Free Interval (DRFI) **(C)** and Breast Cancer-Free Interval (BCFI) **(D)** according to low and high pJNK levels in tumors that do not present an immunomodulatory (IM) phenotype. **(E)**, the distribution of JNK levels across the TNBC molecular subtypes. *P<0.05.

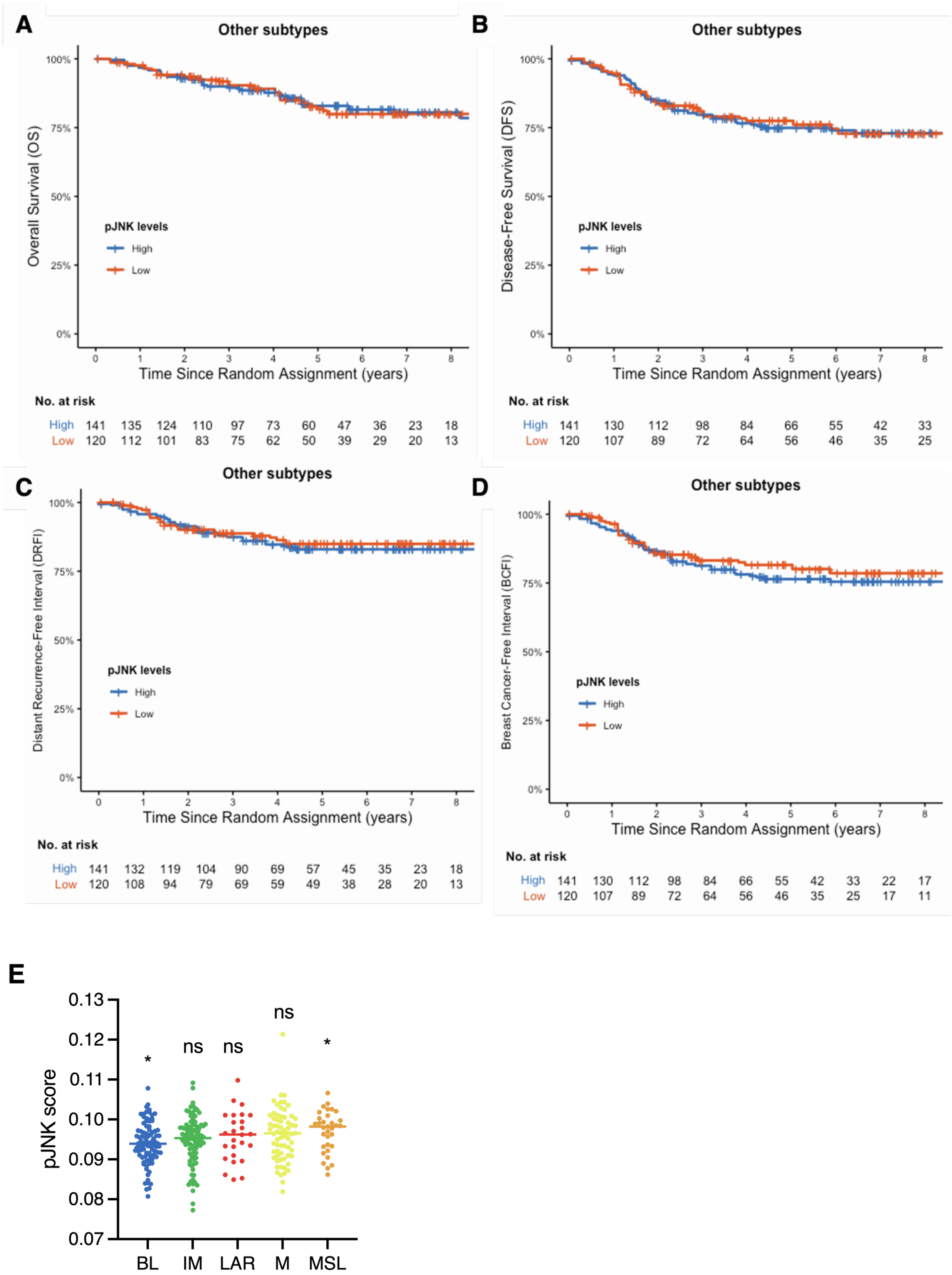

FIGURE S8. Kaplan-Meier TILs > 30% – pJNK levels, Related to Figure 1**:**

Kaplan-Meier estimates of Overall Survival (OS) **(A)**, Disease Free Survival (DFS) (**B**), Distant Recurrence-Free Interval (DRFI) **(C)** and Breast Cancer-Free Interval (BCFI) **(D)** according to low and high pJNK levels in tumors with TILs > 30%.

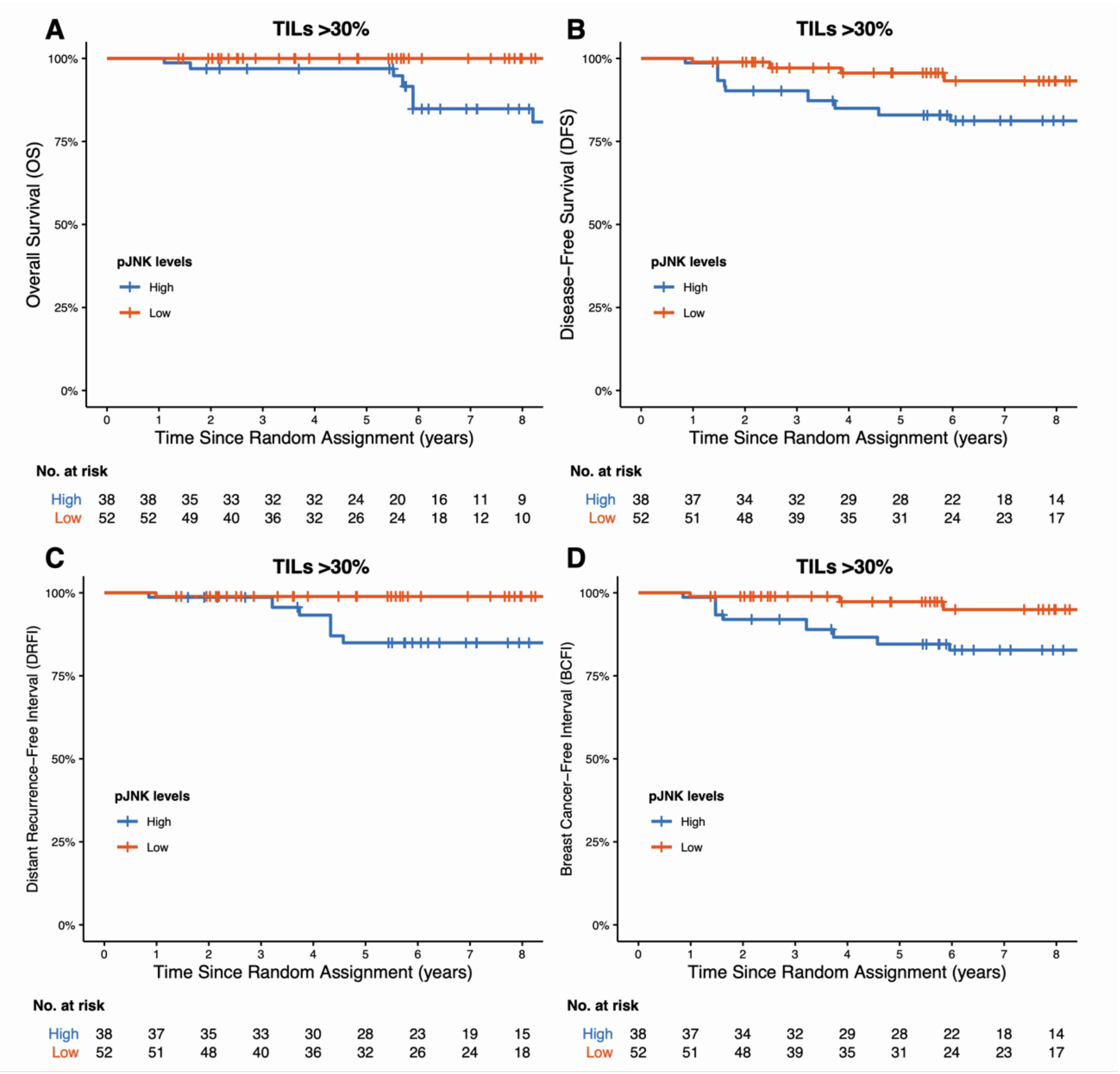

FIGURE S9. Kaplan-Meier TILs < 30% – pJNK levels, Related to Figure 1**:**

Kaplan-Meier estimates of Overall Survival (OS) **(A)**, Disease Free Survival (DFS) (**B**), Distant Recurrence-Free Interval (DRFI) **(C)** and Breast Cancer-Free Interval (BCFI) **(D)** according to low and high pJNK levels in tumors with TILs < 30%.

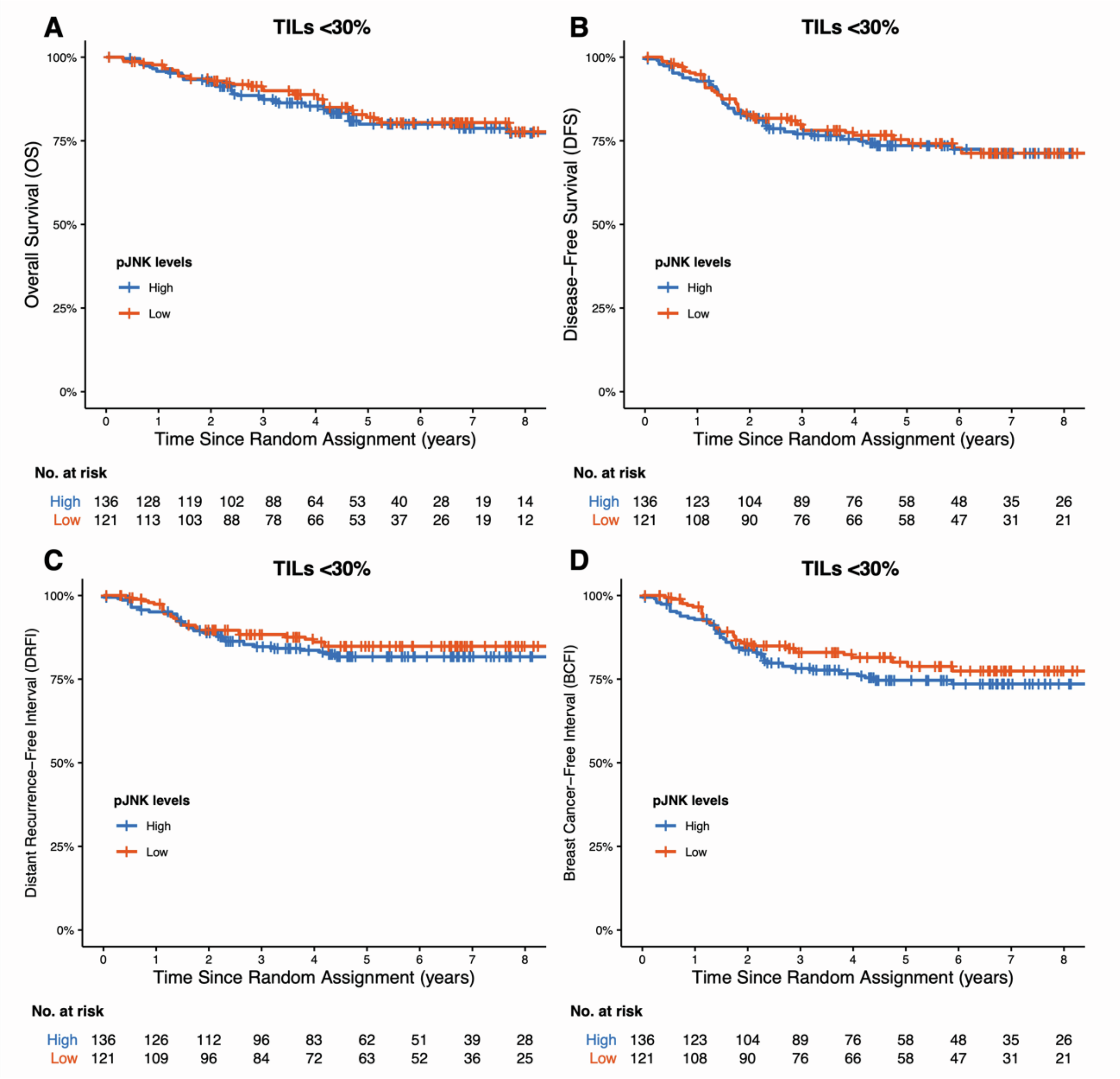

FIGURE S10. Boxplot immune cells – TNBC subtypes, Related to Figure 2:

Boxplot shows the distribution of T-reg cell levels (**B, D**) and the ratio of $CD8^{+}$ T cells to that of T-regs (**C, E**) across low- and high pJNK levels in tumors that do not present an IM phenotype and tumors with low TILs. Boxplot elements: median; box limits, upper and lower quartiles, and whiskers (5 and 95th percentile). P value was determined using the Wilcoxon rank-sum test.

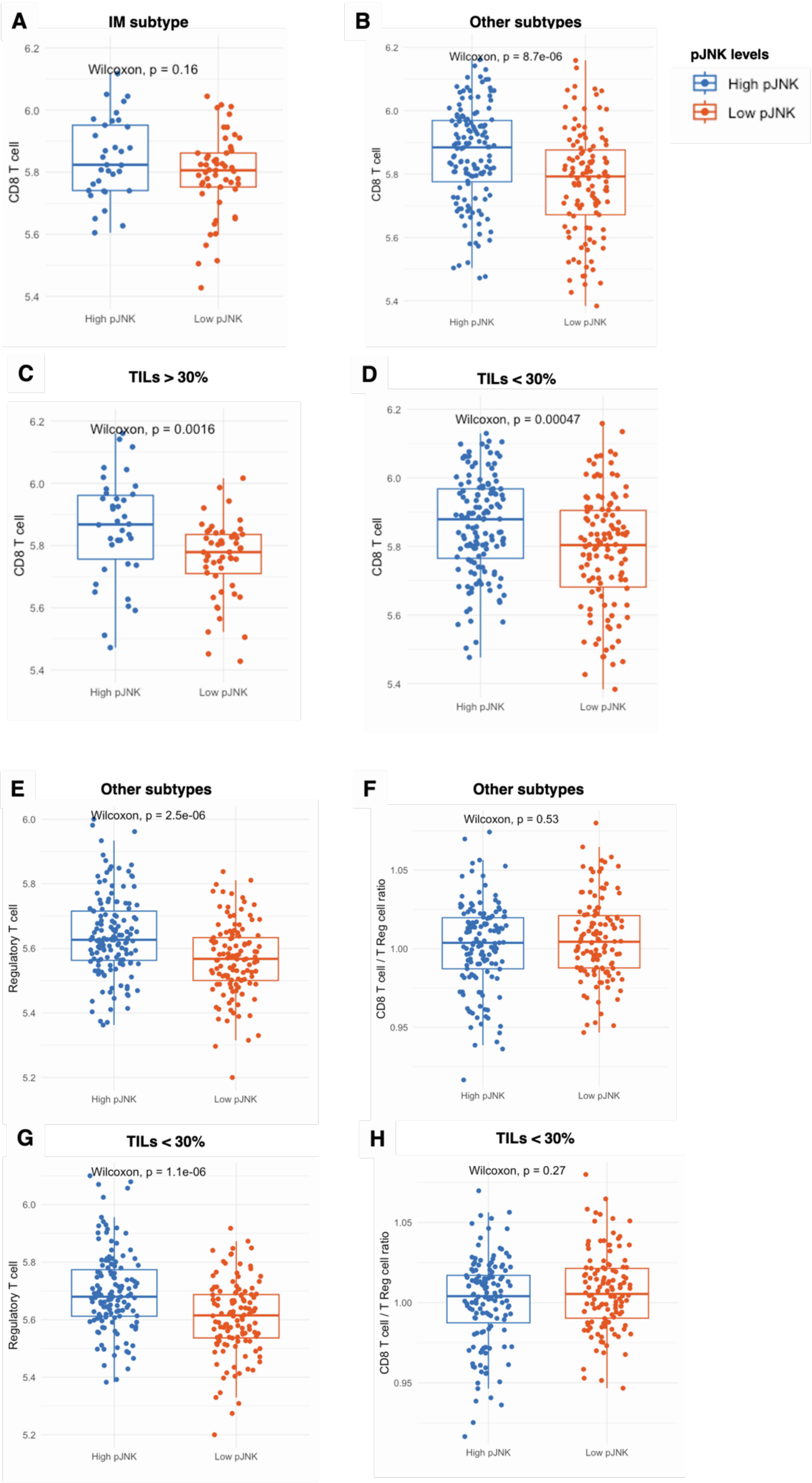

FIGURE S11. TIME subtypes – immune cold tumors, Related to Figure 3**:**
Associations between tumor immune microenvironment (TIME) subtypes and pJNK levels in tumors that do not present IM phenotype (**A**) and tumors with low TILs levels (**B**). P value was determined using Fisher's exact test.

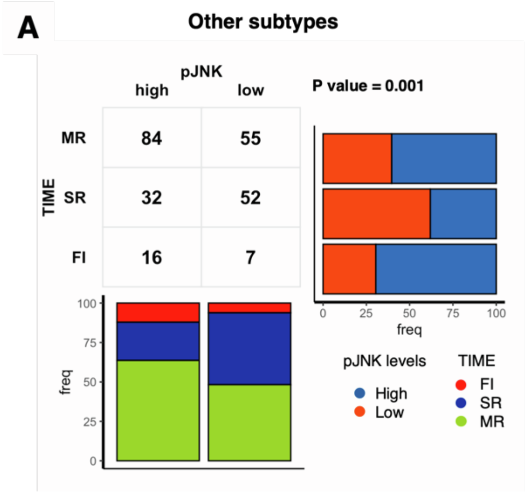

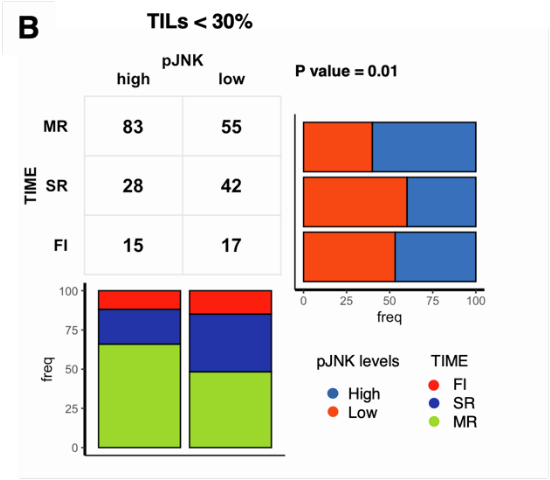

FIGURE S12. Kaplan – Meier IM subtype High pJNK, Related to Figure 4**:**
Kaplan-Meier estimates of overall survival (OS) **(A)**, Distant Recurrence-Free Interval (DRFI) **(B)** and Breast Cancer-Free Interval (BCFI) **(C)** according to CM maintenance for tumors with an immunomodulatory (IM) phenotype and high pJNK levels. CM group represents patients with metronomic treatment whereas No CM group did not receive it. P value represents the Cox proportional hazards mode obtained with the likelihood ratio test.

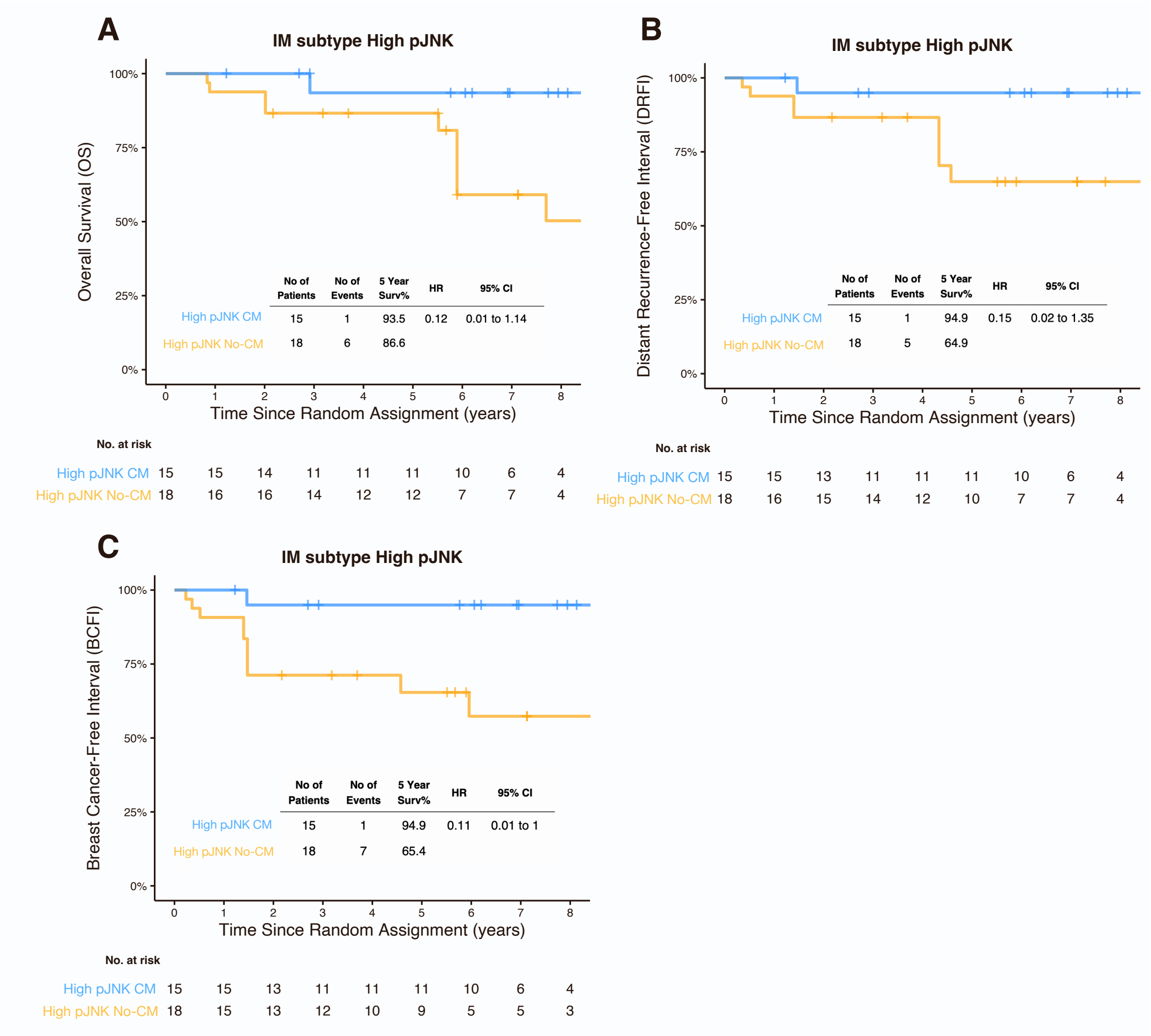

FIGURE S13. Kaplan – Meier IM subtype Low pJNK, Related to Figure 4**:**
Kaplan-Meier estimates of overall survival (OS) **(A)**, Distant Recurrence-Free Interval (DRFI) **(B)** and Breast Cancer-Free Interval (BCFI) **(C)** according to CM maintenance for tumors with an immunomodulatory (IM) phenotype and low pJNK levels. CM group represents patients with metronomic treatment whereas No CM group did not receive it. P value represents the Cox proportional hazards mode obtained with the likelihood ratio test.

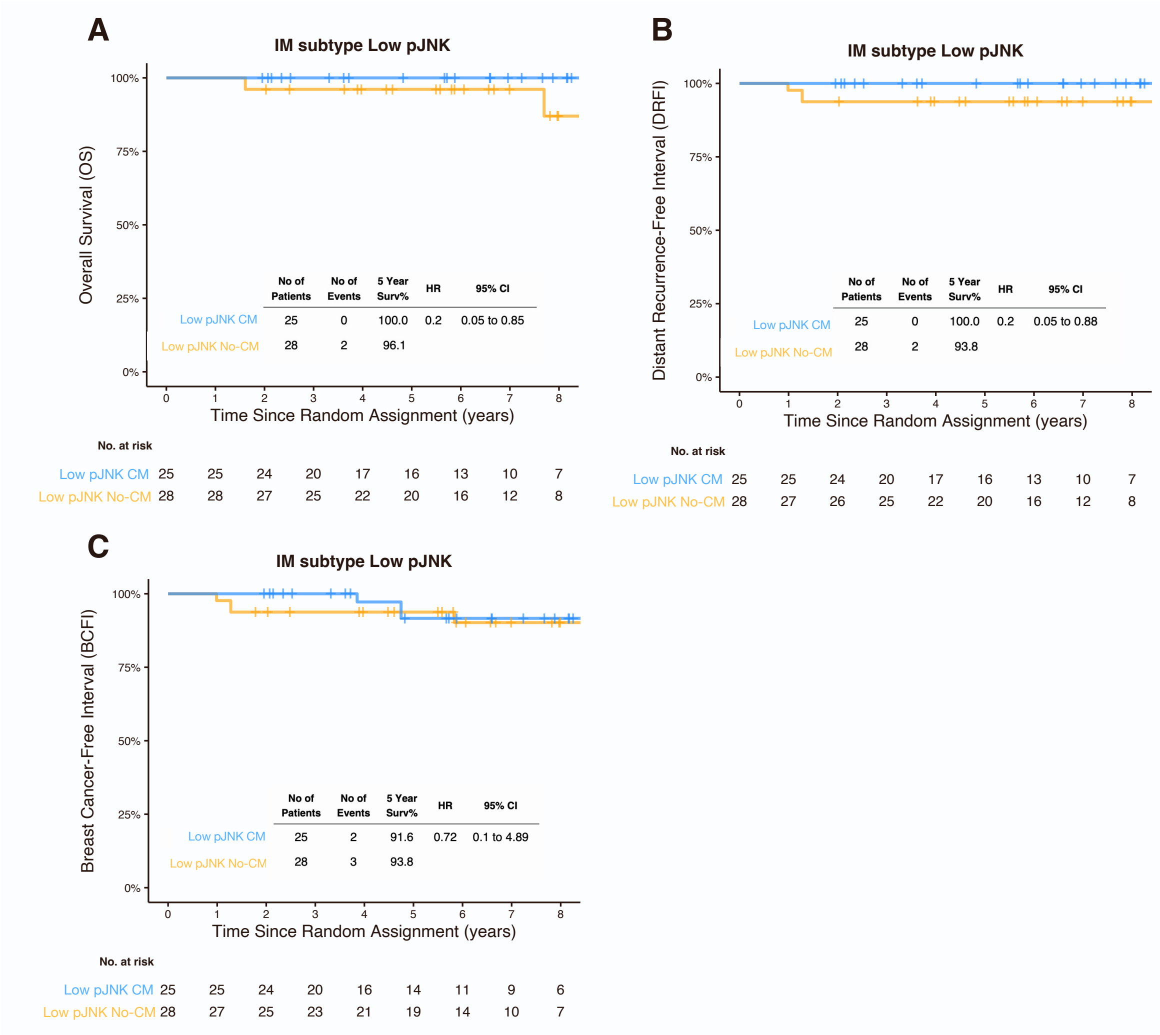

FIGURE S14. Kaplan – Meier sTILs > 30% High pJNK, Related to Figure 4**:**
Kaplan-Meier estimates of overall survival (OS) **(A)**, Distant Recurrence-Free Interval (DRFI) **(B)** and Breast Cancer-Free Interval (BCFI) **(C)** according to CM maintenance for tumors with TILs >30% and high pJNK levels. The CM group represents patients with metronomic treatment whereas the No CM group did not receive it. P value represents the Cox proportional hazards mode obtained with the likelihood ratio test.

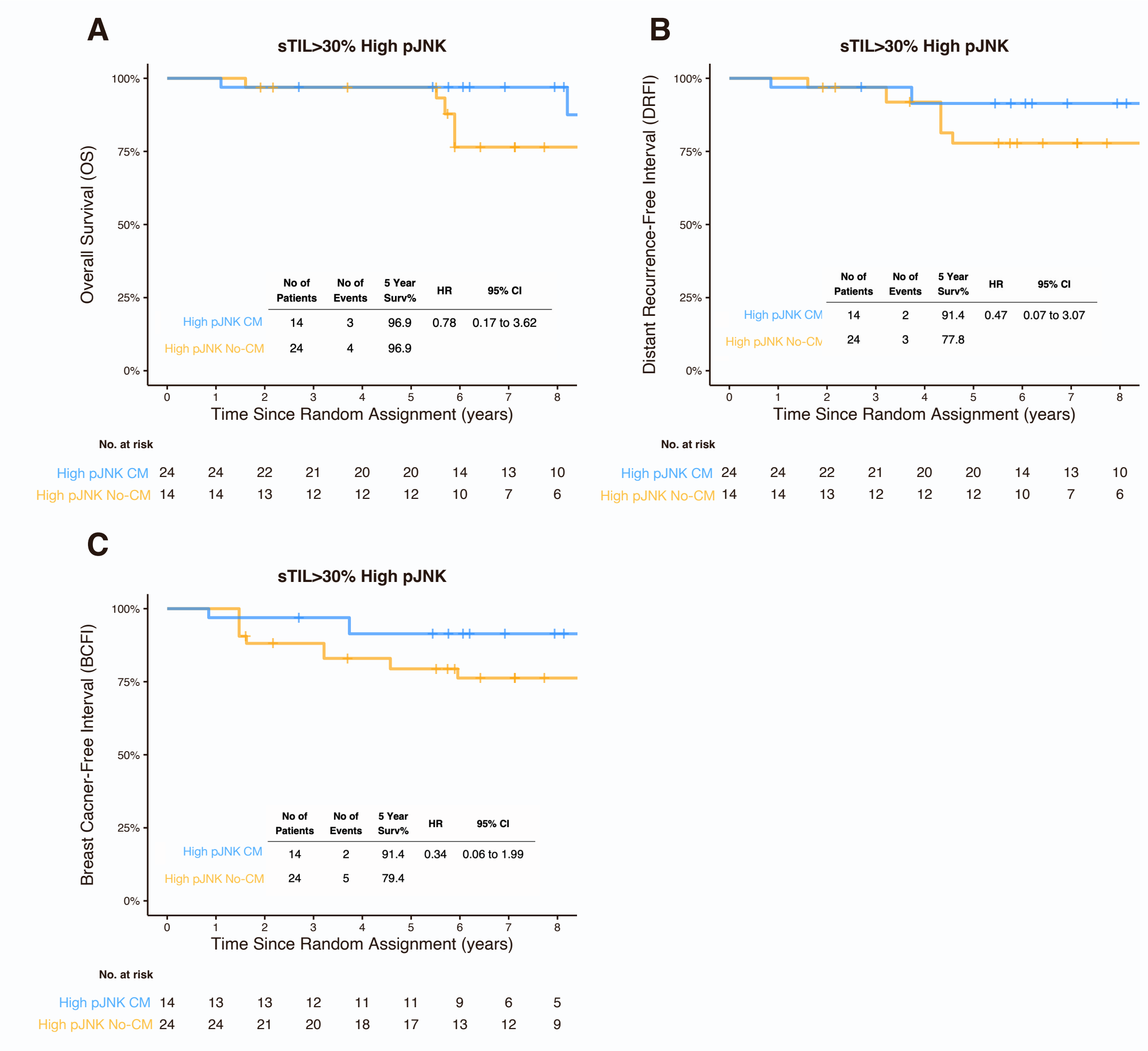

FIGURE S15. Kaplan – Meier sTILs > 30% Low pJNK, Related to Figure 4**:**
Kaplan-Meier estimates of overall survival (OS) **(A)**, Distant Recurrence-Free Interval (DRFI) **(B)** and Breast Cancer-Free Interval (BCFI) **(C)** according to CM maintenance for tumors with TILs >30% and low pJNK levels. The CM group represents patients with metronomic treatment whereas the No CM group did not receive it. P value represents the Cox proportional hazards mode obtained with the likelihood ratio test.

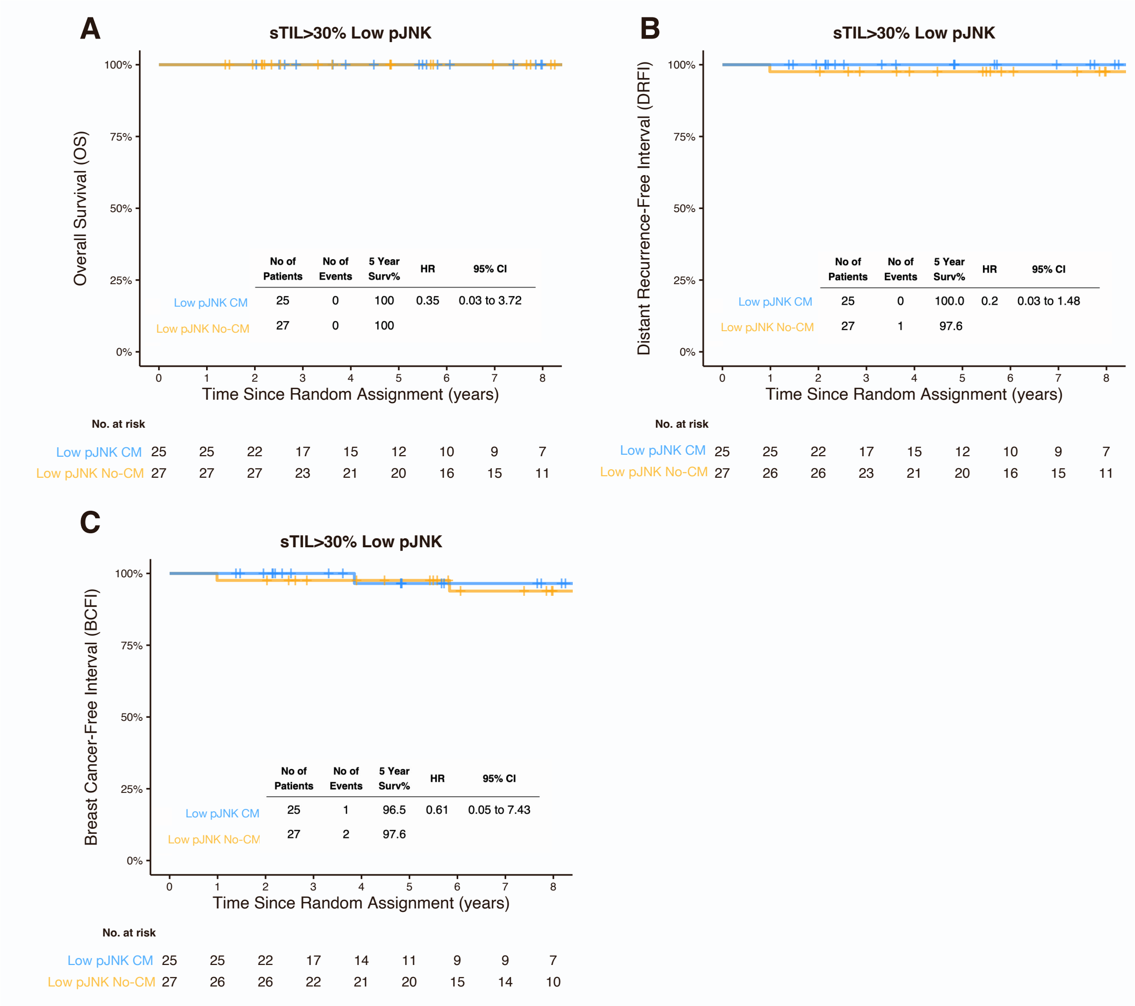

TABLE S1. pJNK signature with coefficients, Related to STAR Methods:
List of genes with the associated coefficient to calculate pJNK levels.

Gene	Coefficient
**FURIN**	0.040719
**CSRP1**	0.0379
**EPHB6**	0.036475
**GIPR**	0.030075
**ACADVL**	0.02653
**CIRBP**	0.022201
**GDPD3**	0.020725
**DEXI**	0.02026
**FER1L4**	0.01994
**MYL6**	0.011294
**OLA1**	0.010666
**TAGLN**	0.010458
**CD2BP2**	-0.00364
**OS9**	-0.01607
**PSMD14**	-0.04127
**FGFR1OP**	-0.04391

TABLE S2. Cox model TILs, Related to Figure 1:

Cox proportional-hazard analysis for associations of TILs levels with DFS overall and according to pJNK levels. The HR inter is defined as the ratio of HR between subgroups with low and high levels of pJNK. P inter represents a Wald test used to assess the interaction between the subtype and pJNK levels.

Endpoint	HR inter	CI 95%	P inter	FDR
OS	0.39	0.11, 1.4	0.14	0.14
DFS	0.27	0.07, 1.00	0.049	0.049
DRFI	0.11	0.011, 1.1	0.061	0.06
BCFI	0.28	0.06,1.3	0.1	0.1

TABLE S3. Cox model pJNK other subtypes, Related to Figure 4:
Cox proportional-hazards models for the comparisons for overall survival (OS), Disease-Free Survival (DFS), Distant Recurrence-Free Interval (DRFI) and Breast Cancer-Free Interval (BCFI) according to CM maintenance and no-CM in tumors that do not present and immunomodulatory subtype (other subtypes).

Endpoint/Cohort	Treatment	N pts	N Events	Multivariate HR (95%CI)
OS				
Other subtype High pJNK	CM maintenance	63	19	1.35 (0.87,2.09)
	No CM	77	10	
Other subtype Low pJNK	CM maintenance	62	10	0.82 (0.53,1.27)
	No CM	59	14	
DFS				
Other subtype High pJNK	CM maintenance	63	23	1.09 (0.78,1.53)
	No CM	77	20	
Other subtype Low pJNK	CM maintenance	62	17	1.11 (0.76,1.62)
	No CM	59	15	
DRFI				
Other subtype High pJNK	CM maintenance	63	16	1.08 (0.69,1.69)
	No CM	77	10	
Other subtype Low pJNK	CM maintenance	62	9	0.96 (0.59,1.58)
	No CM	59	10	
BCFI				
Other subtype High pJNK	CM maintenance	63	20	1.05 (0.72,1.51)
	No CM	77	17	
Other subtype Low pJNK	CM maintenance	62	14	1.13 (0.74,1.72)
	No CM	59	12	

TABLE S4. Cox model pJNK TILs<30%, Related to Figure 4:
Cox proportional-hazards models for the comparisons for overall survival (OS), Disease-Free Survival (DFS), Distant Recurrence-Free Interval (DRFI) and Breast Cancer-Free Interval (BCFI) according to CM maintenance and no-CM in tumors with low levels of TILs (<30%).

Endpoint/Cohort	Treatment	N pts	N Events	Multivariate HR (95%CI)
OS				
TILs <30% High pJNK	CM maintenance	71	12	1.15 (0.49,2.67)
	No CM	64	17	
TILs <30% Low pJNK	CM maintenance	60	11	0.79 (0.3,2.06)
	No CM	62	9	
DFS				
TILs <30% High pJNK	CM maintenance	71	21	0.86 (0.43,1.7)
	No CM	64	21	
TILs <30% Low pJNK	CM maintenance	60	17	1.01 (0.49,2.08)
	No CM	62	18	
DRFI				
TILs <30% High pJNK	CM maintenance	71	12	0.88 (0.37,2.06)
	No CM	64	15	
TILs <30% Low pJNK	CM maintenance	60	16	0.52 (0.22,1.22)
	No CM	62	10	
BCFI				
TILs <30% High pJNK	CM maintenance	71	19	0.86 (0.42,1.76)
	No CM	64	19	
TILs <30% Low pJNK	CM maintenance	60	13	1.15 (0.51,2.57)
	No CM	62	15	

TABLE S5. Clinic-pathologic characteristics for the different TNBC cohorts, Related to STAR Methods:

Among the clinical variables listed, age, nodal status, and tumor stage were included as covariates in the DESeq2 model to adjust for potential confounding effects in differential expression analysis.

	TNBC TILs Cohort (N=647)	RNA (N=347)	RNA (N=347) Weighted	P value (RNA)	P value (RNA weighted)
**Age (years)**				0.22 (1)	0.22 (1)
Mean (SD)	51.233 (10.291)	52.000 (9.574)	51.7 (10.09)		
Range	23.000- 79.000	30.000- 79.000	30.000- 79.000		
**Tumor size**				0.64 (2)	0.64 (2)
<=2cm	291 (45%)	145 (42%)	277 (45%)		
2-5cm	329 (51%)	187 (54%)	317 (52%)		
>5cm	27 (4%)	15 (4%)	19 (3%)		
**Node status**				0.78 (2)	0.77 (2)
N0	370 (57%)	191 (55%)	367 (60%)		
N+ 1-3	171 (27%)	90 (26%)	157 (26%)		
N+ >=4	103 (16%)	66 (19%)	89 (14%)		
**Grade**				1.00 (3)	1.00 (3)
1/2/NA	121 (19%)	69 (20%)	120 (19%)		
3	526 (81%)	278 (80%)	493 (81%)		
**TILs**				0.49 (1)	0.49 (1)
Median (SD)	17.0 (21.047)	15.0 (20.423)	14.5 (20.77)		
Range	0.000- 85.000	0.000- 85.000	0.000- 85.000		
**Treatment**				0.16 (2)	0.86 (2)
Anthracycline [+/- Taxanes +/-CMF]	527 (81%)	295 (85%)	515 (84%)		
No Anthracycline [Taxanes or CMF]	120 (19%)	52 (15%)	98 (16%)		
**Metronomic**				0.90 (3)	0.88 (3)
No CM	307 (47%)	182 (52%)	279 (45%)		
CM Maintenance	340 (53%)	165 (48%)	334 (55%)		

TABLE S6. Clinic-pathologic characteristics for the different treatment arm, Related to STAR Methods:

Among the clinical variables listed, age, nodal status, and tumor stage were included as covariates in the DESeq2 model to adjust for potential confounding effects in differential expression analysis.

	No CM (N=182)	CM (N=165)	p value
**Age (years)**			0.031 (1)
Mean (SD)	53.027 (9.436)	50.867 (9.625)	
Range	30.000- 79.000	31.000- 74.000	
**Tumor size**			0.413 (2)
<=2cm	82 (45.1%)	63 (38.2%)	
2-5cm	93 (51.1%)	94 (57.0%)	
>5cm	7 (3.8%)	8 (4.8%)	
**Node status**			0.463 (2)
N0	104 (57.1%)	87 (52.7%)	
N+ 1-3	42 (23.1%)	48 (29.1%)	
N+ >=4	36 (19.8%)	30 (18.2%)	
**Grade**			0.421 (3)
1/2/NA	33 (18.1%)	36 (21.8%)	
3	149 (81.9%)	129 (78.2%)	
**TILs**			0.476 (1)
Median (SD)	18.0 (20.379)	15.0 (20.509)	
Range	0.000- 85.000	1.000- 80.000	
**Treatment**			0.229 (3)
Anthracycline [+/- Taxanes +/-CMF]	159 (87.4%)	136 (82.4%)	
No Anthracycline [Taxanes and CMF]	23 (12.6%)	29 (17.6%)	
