# Supplementary material for: Role of immunosuppressive JNK pathway in the tumor microenvironment among TNBC subtypes in IBCSG trial 22-00
Source: iScience. 2025 Jun 20;28(8):112964. doi: 10.1016/j.isci.2025.112964 (PMC12355117; doi:10.1016/j.isci.2025.112964)

**CONSORT:**

CONSORT diagram shows the obtention procedure of the final TNBC RNA-seq cohort of 347 patients, 165 assigned to CM-maintenance and 182 to no further chemotherapy (no-CM).


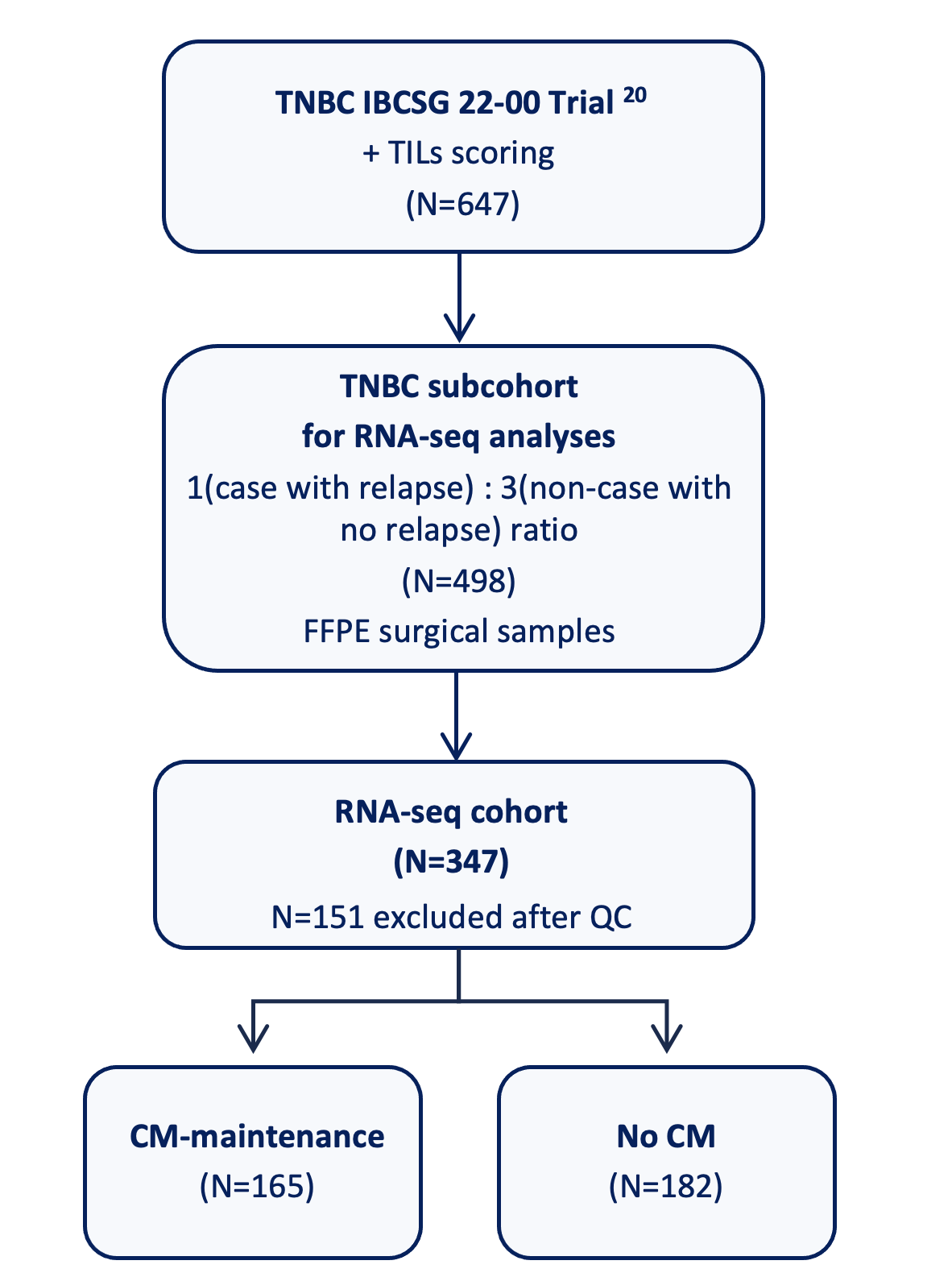

Supplement: Data S2. IBCSG 22-00 clinical trial information [file mmc3.zip › Consort_diagram.docx]
