# Supplementary material for: Role of immunosuppressive JNK pathway in the tumor microenvironment among TNBC subtypes in IBCSG trial 22-00
Source: iScience. 2025 Jun 20;28(8):112964. doi: 10.1016/j.isci.2025.112964 (PMC12355117; doi:10.1016/j.isci.2025.112964)
Supplement: Data S2. IBCSG 22-00 clinical trial information [file mmc3.zip › Statisticalplan_TNBCcohort_IBCSG.docx]

**To: Christos Sotiriou, MD, PhD**

**Cc:** Giuseppe Curigliano MD PhD, Giuseppe Viale MD**,** Meredith M. Regan, ScD**,** Rosita Kammler

**Re: Sampling Design (TR44)**

Project Title: In depth molecular characterization of triple-negative breast cancer including rare histotypes using gene-expression profiling, copy number aberrations analysis and targeted sequencing for adjuvant metronomic chemotherapy benefit assessment.

**From:** Kathryn P Gray, PhD

**Last update:** 02/28/2017; 17 February 2020 by Meredith

**Objective:**

To select samples based on **the centrally assessed triple negative (TN) cohort** of Trial 22-00 ITT population with available TILs results (thereafter refers to **TN+sTILs cohort**)

**Assumptions:**

The project plans to perform deep sequences for N~517 samples using case cohort sampling plan: 1-(case):3-(non-case) ratio, according to Dr Sotiriou’s preference.

**Cases/non-case:** based on the study endpoint of breast cancer free interval (BCFI), which was defined as time from randomization to the breast cancer recurrence (case), or censored (non-case) at date of last follow-up.

Note: in trial 22 TN+TILs cohort, the #s of BC recurrence events (BCFI) n=129 (129/647~20%), if DRFI, then #s of events is 90 (90/647~13%)

**Sampling scheme [1 (case) : 3 (non-case)]**

- All breast cancer recurrence (cases) from the TN+sTILs cohort of trial 22-00 are included (n=129)
- non-breast cancer recurrence (non-cases) are sampled according to the 4 stratification factors below, i.e.,
- Tumor size: 1: T1 (0-2cm), 2: T2 (>2-5cm), 3: T3 (>5cm)
- nodal status: 0: 'N0',1: 'N+ 1-3', 2: 'N+ >=4'
- Age: 1: <40, 2: 40-<50, 3: 50-<60, 4: 60+
- Treatment (tx_itt1): 1: CMM, 2=no-CM

The 4 stratification factors result in 63 strata with patient samples.

**strata level is defined by using 4 digits, each represents one stratification factor,

e.g. strata 1041 corresponds to T1 (1), nodal status: N0(0), age over 60+ and CMM arm(1)

The sampling design is to allocate non-cases (pt ids) into the 63 strata.

The following table gives the distribution of sample #s per each stratum according to the TN+TIL cohort and TR44 cohort, respectively, i.e. the planned/selected TR44 samples (includes both cases and non-cases) has **Ns=517**, in which non-cases (=388) and cases (=129) are balanced via the 63 strata.

|  | **Trial22: TN+TIL cohort (*N=644)** | | **TR44 samples (Ns=517)** | |
| --- | --- | --- | --- | --- |
| **Strata**  **(N=63)** | **Non-case (BCFI)**  **N=515** | **Case (BCFI)**  **N=129** | **Non-case (BCFI)**  **N=388** | **Case (BCFI)**  **N=129** |
| 1011 | 17 | 1 | 4 | 1 |
| 1012 | 8 | 0 | 2 | 0 |
| 1021 | 19 | 5 | 19 | 5 |
| 1022 | 21 | 6 | 21 | 6 |
| 1031 | 34 | 4 | 18 | 4 |
| 1032 | 29 | 5 | 22 | 5 |
| 1041 | 24 | 1 | 4 | 1 |
| 1042 | 18 | 4 | 18 | 4 |
| 1111 | 3 | 4 | 3 | 4 |
| 1112 | 2 | 1 | 2 | 1 |
| 1121 | 13 | 1 | 4 | 1 |
| 1122 | 8 | 0 | 2 | 0 |
| 1131 | 8 | 1 | 4 | 1 |
| 1132 | 10 | 3 | 10 | 3 |
| 1141 | 7 | 0 | 2 | 0 |
| 1142 | 6 | 1 | 4 | 1 |
| 1211 | 1 | 1 | 1 | 1 |
| 1212 | 3 | 1 | 3 | 1 |
| 1221 | 1 | 1 | 1 | 1 |
| 1222 | 4 | 3 | 4 | 3 |
| 1231 | 1 | 2 | 1 | 2 |
| 1232 | 3 | 2 | 3 | 2 |
| 1241 | 1 | 0 | 1 | 0 |
| 1242 | 0 | 1 | 0 | 1 |
| 2011 | 6 | 3 | 6 | 3 |
| 2012 | 10 | 1 | 4 | 1 |
| 2021 | 20 | 2 | 9 | 2 |
| 2022 | 21 | 5 | 21 | 5 |
| 2031 | 26 | 9 | 26 | 9 |
| 2032 | 17 | 4 | 17 | 4 |
| 2041 | 25 | 2 | 9 | 2 |
| 2042 | 11 | 4 | 11 | 4 |
| 2111 | 10 | 2 | 9 | 2 |
| 2112 | 1 | 2 | 1 | 2 |
| 2121 | 11 | 3 | 11 | 3 |
| 2122 | 8 | 2 | 8 | 2 |
| 2131 | 12 | 7 | 12 | 7 |
| 2132 | 14 | 4 | 14 | 4 |
| 2141 | 9 | 2 | 9 | 2 |
| 2142 | 7 | 2 | 7 | 2 |
| 2211 | 2 | 1 | 2 | 1 |
| 2212 | 4 | 1 | 4 | 1 |
| 2221 | 10 | 5 | 10 | 5 |
| 2222 | 6 | 1 | 4 | 1 |
| 2231 | 5 | 1 | 4 | 1 |
| 2232 | 6 | 5 | 6 | 5 |
| 2241 | 5 | 2 | 5 | 2 |
| 2242 | 9 | 3 | 9 | 3 |
| 3012 | 1 | 0 | 1 | 0 |
| 3021 | 1 | 0 | 1 | 0 |
| 3022 | 1 | 0 | 1 | 0 |
| 3032 | 0 | 1 | 0 | 1 |
| 3042 | 4 | 0 | 2 | 0 |
| 3122 | 0 | 2 | 0 | 2 |
| 3131 | 2 | 0 | 2 | 0 |
| 3132 | 1 | 0 | 1 | 0 |
| 3141 | 2 | 0 | 2 | 0 |
| 3212 | 1 | 1 | 1 | 1 |
| 3221 | 0 | 1 | 0 | 1 |
| 3222 | 1 | 2 | 1 | 2 |
| 3232 | 1 | 0 | 1 | 0 |
| 3241 | 2 | 1 | 2 | 1 |
| 3242 | 2 | 0 | 2 | 0 |

* three non-case patients had no nodal status, dropped from the sampling

**Sample/power justifications:**

Assumes the BCFI endpoint follows the exponential distribution, with 12 year accrual, two additional follow-up years (~median FU of 7 years), and with the planned sample size (N=517, BCFI events=129), the following table summarizes the hazard ratio (HR) to be detected with 80% power, using a log-rank test to compare the BCFI distribution between groups defined by mutational status (yes for g1; no for g2) with a type I error of 5%.

| **Sample size N=517, BCFI events=129** | | |
| --- | --- | --- |
| Mutation prevalence | HR (g1 worse/g2) | HR (g1 better/g2) |
| 0.1 | 1.98 | 0.25 |
| 0.15 | 1.82 | 0.39 |
| 0.2 | 1.74 | 0.47 |
| 0.3 | 1.66 | 0.54 |

**Analysis plan (details to come before analysis)**

A weighted analysis method (2009 Gray) with breast cancer free interval outcome (generalized Horvitz-Thompson methods) will be used to adjust Kaplan-Meier and hazard ratio estimates and Wald test statistics to obtain unbiased analyses and to give consistent estimates of effects in the full TN+TIL cohort. Weights will be computed as the inverse of the proportion of the patients sampled within each of the sampling stratum from the TR44 cohort, separately for recurrences and non-recurrences.

Ref: Gray R. Weighted analyses for cohort sampling designs. Lifetime Data Anal 15(1):24-40, 2009

For your analysis plan:

As noted above, there are 63 possible strata defined by 4 features. Sample weights are created separately for events and non-events and thus 126 classes.

For each class, the weight is:

*w_k_ = N_k_ / n_k_*

where N_k_ is number eligible patients in the class and n_k_ is the number successfully assessed in the class

(so sum{N_k_}=647; and sum{n_k_}=# successful; for k=1…126).

I’ve provided a dataset for all 647 patients; the notes above describe only 517 patients’ strata (and I believe IBCSG sent less than 517 patients’ RNA because some wasn’t available, but that doesn’t matter). At this point, for each of the 647 patients, identify which of the 126 classes they belong, as defined by the event/non-event status and the 4 features. Then for each unique class (k=1..126), calculate Nk, nk and wk. Note some of the 126 classes won’t be represented ad that’s OK.

In the analyses, the sampling weights are needed to get appropriate point estimates and standard errors. Most functions in SAS and R allow one to easily incorporate sampling weights; the weighted point estimates in these functions are usually quite good but the SEs are usually slightly too small (per research by Bob Gray). For many analyses, if just treating this as a case-control study to investigate association of variables with binary case-control status, then the inference from should be OK. So many analytical tools for binary outcome status should work fine. But once we want to go back to time-to-event outcome analysis or if we want to estimate prevalence of mutations in the population, then the sampling weights are key.

To illustrate the necessity for sampling weights (using SAS, in an example from the BIG 1-98 trial):

1. Note that the distant recurrence event proportion is *much* higher (26%) than the overall eligible population (11.5%) and the 5-yr DRFI is incorrect unless weighted:

|  | Distant Recurrence | | | | Total |  |  |  |
| --- | --- | --- | --- | --- | --- | --- | --- | --- |
|  | 0:no | | 1:yes | |  |  | 5-yr DRFI ± SE | Weighted 5-yr DRFI ± SE |
|  | N | % | N | % | N |  |  |  |
| Case-cohort set | 398 | 74.0 | 140 | *26.0* | 538 |  | 83.1 ± 0.016 | 93.1 ± 0.017 |
| All eligible | 6488 | 88.5 | 841 | *11.5* | 7329 |  | 93.0 ± 0.003 |  |

So the weighted estimate is quite close to the full cohort; but the SE isn’t quite right by using a weight statement in PROC LIFETEST. When possible, use robust SE estimates (not possible in SAS proc lifetest)

Note when we create a KM plot, we report the observed numbers of patients & events analyzed, observed numbers of patients at risk over time, together with the weighted test statistic and p-value weighted KM estimate & SE (and weighted HR & 95% CI) based upon robust variance estimate.

2. The relation of a covariate with outcome could be incorrect if it is associated with case-cohort status, consider for example nodal status:

“truth” eligible cohort 7329 pts (N+ vs N-), Wald test chi-square stat=266, p<0.0001

| Analysis of Maximum Likelihood Estimates | | | | | | | | | | |
| --- | --- | --- | --- | --- | --- | --- | --- | --- | --- | --- |
| Parameter |  | DF | Parameter Estimate | Standard Error | Chi-Square | Pr > ChiSq | Hazard Ratio | 95% Hazard Ratio Confidence Limits | | Label |
| PN3_LVL | 1 | 1 | 1.20557 | 0.07387 | 266.3443 | <.0001 | 3.339 | 2.889 | 3.859 | PN3_LVL 1 |

Unweighted sample 538, Wald test chi-square stat=0.74

| Analysis of Maximum Likelihood Estimates | | | | | | | | | | |
| --- | --- | --- | --- | --- | --- | --- | --- | --- | --- | --- |
| Parameter |  | DF | Parameter Estimate | Standard Error | Chi-Square | Pr > ChiSq | Hazard Ratio | 95% Hazard Ratio Confidence Limits | | Label |
| PN3_LVL | 1 | 1 | 0.14684 | 0.17050 | 0.7417 | 0.3891 | 1.158 | 0.829 | 1.618 | PN3_LVL 1 |

Weighted sample 538, Wald test chi-square stat=262

| Analysis of Maximum Likelihood Estimates | | | | | | | | | | |
| --- | --- | --- | --- | --- | --- | --- | --- | --- | --- | --- |
| Parameter |  | DF | Parameter Estimate | Standard Error | Chi-Square | Pr > ChiSq | Hazard Ratio | 95% Hazard Ratio Confidence Limits | | Label |
| PN3_LVL | 1 | 1 | 1.37617 | 0.08499 | 262.1605 | <.0001 | 3.960 | 3.352 | 4.677 | PN3_LVL 1 |

Here again the SE on the weighted parameter estimate isn’t quite right but, but it’s pretty close to what it should be according to Bob Gray’s methodology (se=0.087) rather than the SAS-implemented (also R-implemented in coxph, se=0.085). And you see for example that the weighted test should give approximately the right answer from SAS/R implementation of weights.

3. The frequency of N+ in the eligible population would be incorrect if not estimated using the weights:

“Truth” 2969/7329 (40.5%), 95% CI (0.394-0.416)

Unweighted sample 289/538 (53.7%)

Weighted sample 36.9%, 95% CI (0.367-0.381)

So again the weighted estimate is close to ‘truth’ but the CI is too small here.
