# Supplementary material for: Role of immunosuppressive JNK pathway in the tumor microenvironment among TNBC subtypes in IBCSG trial 22-00
Source: iScience. 2025 Jun 20;28(8):112964. doi: 10.1016/j.isci.2025.112964 (PMC12355117; doi:10.1016/j.isci.2025.112964)
Supplement: Data S2. IBCSG 22-00 clinical trial information [file mmc3.zip › IBCSG 22-00_Amend5.pdf]

INTERNATIONAL BREAST CANCER STUDY GROUP
Trial 22-00

IBCSG

Maintenance Chemotherapy in Hormone Non-Responsive Breast Cancer

AMENDMENT 5

Low-dose Cytotoxics as "Anti-angiogenesis Treatment" following Adjuvant Induction Chemotherapy for Patients with ER-negative and PgR-negative Breast Cancer

Approved Induction Chemotherapy

versus

Approved Induction Chemotherapy
followed by
"Anti-angiogenesis" Chemotherapy with
Continuous Oral CM Therapy for 12 Months
(CM Maintenance)

IBCSG Trial 22-00

Maintenance Chemotherapy in Hormone Non-Responsive Breast Cancer

IBCSG Centers

**Study Chair**	Dr. Marco Colleoni<br>European Institute of Oncology<br>Division of Medical Oncology<br>Via Ripamonti 435<br>20141 Milano, Italy	Tel: +390 2 574 89498<br>Fax: +390 2 574 89212<br>marco.colleoni@ieo.it
**Coordinating Center**	Dr. Rudolf Maibach<br>Anita Hiltbrunner	Tel: +41 31 389 93 91<br>Fax: +41 31 389 93 92<br>rudolf.maibach@ibcsg.org<br>anita.hiltbrunner@ibcsg.org
Safety Office<br>Medical Affairs	Dr. Barbara Ruepp<br>Dr. Manuela Rabaglio	safetyoffice@ibcsg.org<br>manuela.rabaglio@ibcsg.org
Quality of Life Office	PD Dr. Jürg Bernhard<br>IBCSG Coordinating Center<br>Effingerstrasse 40<br>CH-3008 Bern, Switzerland	juerg.bernhard@ibcsg.org
**Data Management Center**	Lynette Blacher, Director<br>Joie Celano, Lead Data Manager<br>Holly Shaw, Lead Trial Coordinator<br>Frontier Science & Technology Research Foundation (FSTRF)<br>4033 Maple Road<br>Amherst, NY 14226 USA	Tel: +1 716 898-7500<br>Fax: +1 716 836 6097<br>dmc22@fstrf.org
**Statistical Center**	Prof. Richard Gelber, Director<br>Shari Gelber, Trial Statistician<br>Dept. of Biostatistical Science<br>Dana-Farber Cancer Institute<br>44 Binney Street<br>Boston, MA 02115 USA	Tel: +1 617 632 3603<br>Fax: +1 617 632 2444<br>shari@jimmy.harvard.edu<br>gelber@jimmy.harvard.edu
**Scientific Committee Chairs**	Prof. Aron Goldhirsch<br>European Institute of Oncology<br>Division of Medical Oncology<br>Via Ripamonti 435<br>20141 Milano, Italy	Tel: +39 0 2 574 89439<br>Fax: +41 91 8056517<br>aron.goldhirsch@ibcsg.org
	Prof. Alan Coates<br>40 Cook Road<br>Centennial Park NSW 2021<br>Australia	Tel: +61 2 9331 3521<br>Fax: +61 2<br>email:<br>alan.coates@ibcsg.org
**Central Pathology Chair**	Prof. Giuseppe Viale<br>European Institute of Oncology<br>Division of Medical Oncology	Tel: +390 2 574 89420<br>Fax: +390 2 574 89537<br>giuseppe.viale@ieo.it

Protocol Amendment 5 Signature Page

IBCSG Trial 22-00
Maintenance Chemotherapy in Hormone Non-Responsive Breast Cancer

Approved by:
Director of the Statistical and Data Management Center
Prof. R. D. Gelber

(Signature on file) *12 August 2010*

Date

Protocol Signature Page

IBCSG Trial 22-00
Maintenance Chemotherapy in Hormone Non-Responsive Breast Cancer

Approved by:
CEO, International Breast Cancer Study Group
Prof. M. Castiglione

(*Signature on file*)

_______________________________ _______________

Date

Approved by:
Group Statistician, International Breast Cancer Study Group
Prof. R. D. Gelber

(*Signature on file*)

_______________________________ _______________

Date

Principal Investigator Protocol Signature Page

IBCSG Trial 22-00

Maintenance Chemotherapy in Hormone Non-Responsive Breast Cancer

I have read the protocol and agree that it contains all necessary details for conducting this study. I will conduct the study as outlined in the following protocol and in compliance with GCP. I will provide copies of the protocol and all drug information relating to pre-clinical and prior clinical experience furnished to me by IBCSG, **to all physicians responsible to me who participate in this study. I will discuss this material with them to assure that they are fully informed** regarding the drug and the conduct of the study. I agree to keep records on all patient information (Case Report Forms and patient's informed consent statement) and all other information collected during the study for a minimum period of 15 years.

Name of Principal Investigator:______________________________________

____ ____________

Signature Date

Registration Procedure (Section 4.2)

1. Verify eligibility.
2. Obtain written informed consent for both the clinical trial and the pathology material submission, signed and dated by the patient and investigator (see section 12.2 and Appendix I).
3. Complete Confirmation of Registration Form (A). The date the Informed Consent Form was signed by the patient and the date it was signed by the investigator are both required to complete randomization.
4. Complete the pre-randomization Quality of Life (QL) core and module forms. (See Section 3.1.6 for exceptions.)
5. Depending on your Group's choice, either:
   - Telephone or fax your Randomization Center to review the eligibility and randomization information. Your Randomization Center will access the IBCSG Registration/Randomization System

   or
   - Directly access the IBCSG Registration/Randomization System

   If you telephone or fax the information, the Randomization Center will provide the Participating Center with the following information, as well as with the name of the person performing the randomization. In the latter case, the Randomization System will provide this information via email.
   - Randomization number (Patient ID)
   - Treatment assignment
   - Date of randomization
6. When the randomization is complete, fill in the Confirmation of Registration Form (A) with the information above and fax or mail the completed A and PMC Forms to the IBCSG DMC.

   This completed A Form is considered the essential document for regulatory purposes.
7. Mail the pre-randomization Quality of Life (QL) Core and Module Forms to the IBCSG DMC.
8. File your copy of the completed Confirmation of Registration Form (A)

*Randomization Centers:

FSTRF Randomization Help Desk
Frontier Science & Technology Research Foundation (FSTRF)
4033 Maple RD, Amherst, NY 14226 USA
Phone: +1 716 834 0900 ext. 7301
Fax: +1 716 836 6097
Email: bc.helpdesk@fstrf.org

NHMRC Clinical Trials Centre, University of Sydney,
Locked Bag 77, Camperdown NSW 2050, Australia
Australia: 1-800-027-928
New Zealand & Hong Kong: 00-800-0279-2888
Fax: +61 2 552 3881

IBCSG TRIAL 22-00: Maintenance Chemotherapy in Hormone Non-Responsive Breast Cancer

Protocol Summary and Schema

Patient Population: Patients with histologically proven breast cancer and who are ER-negative and PgR-negative (< 10% positive cells by immunohistochemical assay), who have had either a total mastectomy with axillary clearance or a lesser procedure (quadrantectomy or lumpectomy) with axillary lymph node dissection or sentinel node procedure, and are classified as $T_{1a,b,c}$, T_2, T_3, or pT_4 with minimal dermal invasion, N_X, pN_0, $pSentN_0$, pN_1, or pN_2, and M_0. Patients with disease defined as SNB positive are eligible only if they have undergone an axillary dissection, OR are SNB micrometastatic and are randomized to IBCSG Trial 23-01.

Timing: Patients must begin induction chemotherapy within 8 weeks of definitive breast cancer surgery. Patients must be randomized within the following "Randomization Window" (see figure below): after definitive surgery but within 56 days after the first day of the last cycle of induction chemotherapy.

Figure 1

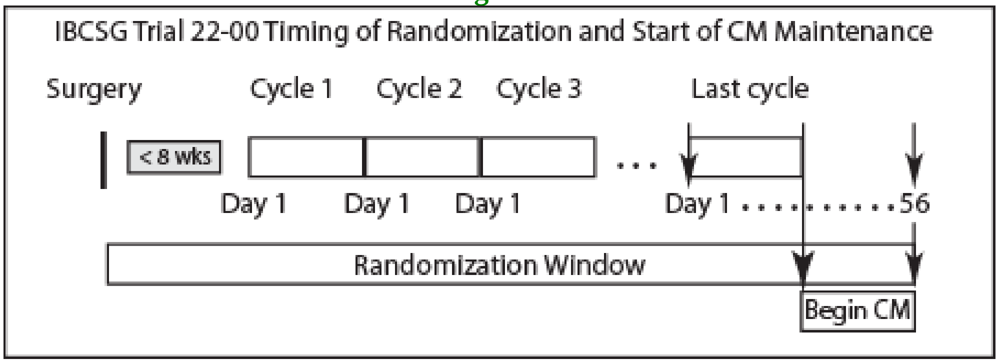

Stratification Factors:
-Institution
-Menopausal status (pre versus post)
-Induction chemotherapy (AC/ECx4 versus other regimens)

Randomization: Approved **induction chemotherapy vs.** approved **induction chemotherapy followed by CM Maintenance**

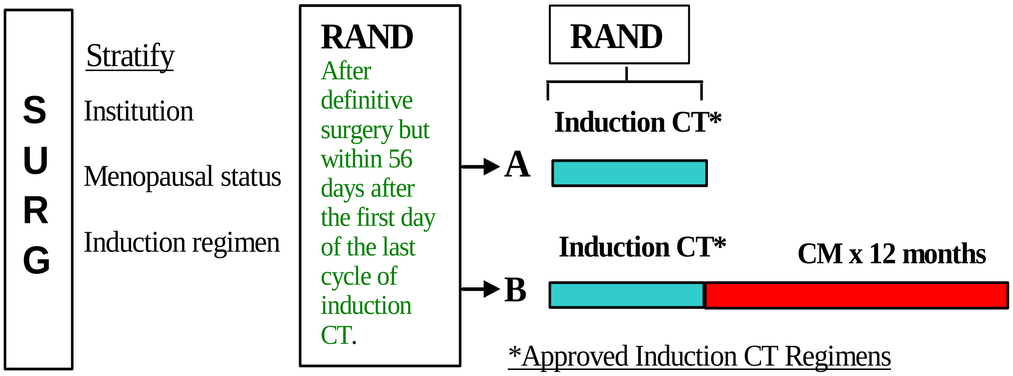

Treatment Schedules:
Radiotherapy
For all patients receiving adjuvant chemotherapy after breast conservation, radiation therapy to the conserved breast is mandatory, to be carried out according to the prospectively defined guidelines of each participating institution; either after all chemotherapy or integrated into CMF as agreed per institution. Radiotherapy to the chest wall following mastectomy is optional according to the prospectively defined guidelines of each participating institution (if given, it may include also nodal fields). **Radiotherapy must not delay the beginning of the maintenance chemotherapy.**

Trastuzumab therapy
Trastuzumab (Herceptin®) may be given to patients with HER2-positive primary breast cancer.

CM Maintenance Chemotherapy
C=cyclophosphamide 50 mg/day orally continuously for one year (365 days)
M=methotrexate 2.5 mg/twice a day orally days 1 and 2 of every week for one year (52 weeks)

CM maintenance must begin after the last day of the last cycle of induction chemotherapy (eg, day 22 of AC or day 29 of CMF), but within 56 days after the first day of the last cycle (See Figure 1); and continue for 365 days.

The total time spent receiving CM maintenance should not exceed 365 days (1 year) regardless of treatment delays and modifications.

Table 1: Approved* Induction Regimens

	**AC/EC x 4**	
**AC/EC**	A = doxorubicin 60 mg/m^2 **OR** E = epirubicin 90 mg/m^2 iv day 1<br>C = cyclophosphamide 600 mg/m^2 iv day 1<br>(Doxorubicin or epirubicin should be administered intravenously using all precautions to avoid extravasation.)	Repeat every 21 days
	**CMF x 6**	
**Classical CMF**	C= cyclophosphamide 100 mg/m^2 orally days 1-14<br>M= methotrexate 40 mg/m^2 iv days 1 and 8<br>F= 5-fluorouracil 600 mg/m^2 iv days 1 and 8<br>(Methotrexate and 5-fluorouracil should be administered by i.v. push injection with methotrexate preceding 5-fluorouracil.)	Repeat every 28 days
	**AC/EC x 4 followed by CMF x 3**	
	Use above dosages of AC/EC and classical CMF	
	**$CEF_{1,8}$ x 6**	
**$CEF_{1,8}$**	C= cyclophosphamide 75 mg/m^2 orally days 1-14<br>E= epirubicin 60 mg/m^2 iv days 1 and 8<br>F= 5-fluorouracil 500 mg/m^2 iv days 1 and 8<br>(with antibiotic support)	Repeat every 28 days
	**$CAF_{1,8}$ x 6**	
**$CAF_{1,8}$**	C= cyclophosphamide 100mg/m^2 orally days 1-14<br>A= doxorubicin 30 mg/m^2 iv days 1 and 8<br>F= 5-fluorouracil 500 mg/m^2 iv days 1 and 8	Repeat every 28 days
	**FEC_{100}**	
**FEC_{100}**	C= cyclophosphamide 500mg/m^2 iv day 1<br>E= epirubicin 100 mg/m^2 iv day 1<br>F= 5-fluorouracil 500 mg/m^2 iv day 1	Repeat every 21 days

**AC/EC x 4 followed by T x 4**		
**AC/EC:**	A = doxorubicin 60 mg/m^2 **OR** E = epirubicin 90 mg/m^2 iv day 1<br>C = cyclophosphamide 600 mg/m^2 iv day 1<br>(Doxorubicin or epirubicin should be administered intravenously using all precautions to avoid extravasation.)	Repeat every 21 days
**Taxol/ Taxotere**	T= paclitaxel 175 mg/m^2 iv **OR** docetaxel 100 mg/m^2 iv day 1	Repeat every 21 days
**iv FACx 6**		
**iv FAC**	C= cyclophosphamide 600 mg/m^2 iv day 1<br>A= doxorubicin 60 mg/m^2 iv day 1<br>F= 5-fluorouracil 600 mg/m^2 iv day 1	Repeat every 21 days
**Ax4 followed by iv CMFx4**		
**Ax4**	A= doxorubicin 75 mg/m^2	Repeat every 21 days
**iv CMF**	C= cyclophosphamide 600 mg/m^2 iv day 1 and 8<br>M= methotrexate 40 mg/m^2 iv day 1 and 8<br>F= 5-fluorouracil 600 mg/m^2 iv day 1 and 8	Repeat every 28 days
**Dose Dense ACT x 4**		
AC	A = doxorubicin 60 mg/m^2 iv day 1 OR epirubicin 90 mg/m2 iv day 1<br>C = cyclophosphamide 600 mg/m2 iv day 1	Repeat every 14 days
T	T = paclitaxel 175 mg/m^2 with filgrastim (GSF)	Repeat every 14 days
**FEC$_{100}$ x 3 followed by T x 3**		
FEC$_{100}$	C= cyclophosphamide 500mg/m^2 iv day 1<br>E= epirubicin 100 mg/m^2 iv day 1<br>F= 5-fluorouracil 500 mg/m^2 iv day 1	Repeat every 21 days
Taxotere	T= docetaxel 100 mg/m^2 iv day 1	Repeat every 21 days
**TAC x 6**		
TAC	T= docetaxel 75 mg/m^2 iv day 1<br>A= doxorubicin 50 mg/m^2 iv day 1<br>C= cyclophosphamide 500mg/m^2 iv day 1	Repeat every 21 days
**A x 3 followed by T x 3 followed by classical CMF x 3**		
A	A = doxorubicin 75 mg/m^2	Repeat every 21 days
T	T= docetaxel 100 mg/m2 iv day 1	Repeat every 21 days
Classical CMF	C= cyclophosphamide 100 mg/m^2 orally days 1-14<br>M= methotrexate 40 mg/m^2 iv days 1 and 8<br>F= 5-fluorouracil 600 mg/m^2 iv days 1 and 8<br>(Methotrexate and 5-fluorouracil should be administered by i.v. push injection with methotrexate preceding 5-fluorouracil.)	Repeat every 28 days

*Induction regimens must be approved by the IBCSG Scientific Committee

TABLE OF CONTENTS

Section		Page
1	Introduction	12
2	Trial objectives	16
3	Patient selection	16
	3.1 Criteria for patient eligibility	16
	3.2 Criteria for patient ineligibility	18
4	Stratification and randomization	18
	4.1 When to randomize	18
	4.2 Registration procedures	18
	4.3 Randomization centers	19
	4.4 Information required at registration	20
	4.5 Randomized groups	20
	4.6 Stratification	20
5	Treatment details	21
	5.1 Trial drug treatments	21
	5.2 Side effects of study drugs	22
6	End points and definitions of treatment failure	24
	6.1 Trial end points	24
	6.2 Diagnosis of treatment failure	25
7	Study parameters	28
	7.1 Table of study parameters	28
	7.2 Adverse event reporting	31
	7.3 Serious Adverse Event (SAE) reporting	31
8	Data collection and submission	33
	8.1 Case report forms schedule	33
	8.2 Pathology materials submission	34
	8.3 Data management	34
9	Statistical considerations	35
	9.1 Study design, objectives and stratification	35
	9.2 Data analyses	35
	9.3 Sample size considerations	35
	9.4 Accrual estimates, time frame for analyses, and interim monitoring	36
	9.5 Data Safety Monitoring Committee	36

Section **Page**

10 Quality of Life 37
10.1 Introduction 37
10.2 Hypotheses 37
10.3 Additional analyses 37
10.4 Patient selection 38
10.5 Study design 38
10.6 Quality-of-life measures 38
10.7 Timing requirements, data collection and local data management 41
10.8 Central data management 42
10.9 Statistical considerations 42

11 Additional protocol-specific parameters and sub-studies 42
11.1 Hormone receptors 42
11.2 Pathology and pathology material banking 43
11.3 c-erbB-2 44
11.4 Angiogenic activity 44
11.5 Family history 44

12 Regulatory approval procedures and patient informed consent 44
12.1 Ethical Review Board/Ethics Committee 44
12.2 Regulatory approval procedures 45
12.3 Informed consent 45

13 Administrative considerations 45
13.1 Insurance 45

14 References 45

Appendices

I. Patient Information and Informed Consent (revised for amendment 5)
II. NCI Common Toxicity Criteria, Version 2
[available from the internet at: http://ctep.cancer.gov/reporting/CTC-3.html]
III. Guidelines for Immunocytochemical Evaluation of Hormone Receptor Status and Quality Assurance
IV. Pathology Guidelines

1 Introduction

1.1 Efficacy of adjuvant chemotherapy in breast cancer with low or no hormone receptors. Adjuvant treatments with chemotherapy, endocrine therapy, and combinations of both have been shown to prolong disease-free survival and to reduce mortality in patients with node-positive and node-negative breast cancer (1). As shown in a meta-analysis of adjuvant therapy trials, chemotherapy reduced the odds of recurrence by 28 percent at 10 years follow-up. In premenopausal patients, prolonged combination chemotherapy was more effective than single agent or brief perioperative chemotherapy.

1.2 Duration of adjuvant CMF in the subpopulation of patients with tumors containing low or no hormone receptors: CMFx6. The IBCSG conducted a clinical trial in premenopausal women with node-positive breast cancer (IBCSG Trial VI) with the aim of studying both the duration of adjuvant chemotherapy [3 versus 6 initial cycles of cyclophosphamide, methotrexate and fluorouracil (CMF)] and the reintroduction of single courses of delayed chemotherapy, targeting the hypothesized phases of fast growth (2). At a median follow-up of 72 months, the comparison between 3 and 6 cycles of initial CMF favored the 6 cycles, especially for patients aged 39 or younger. Reintroduction chemotherapy substantially improved results (hazards ratio 0.86) especially for women aged 40 years or older (1167 patients, hazards ratio 0.82). In particular, a decreased relapse rate was observed at about 2.5 years for the group with 6 initial CMF cycles and reintroduction chemotherapy. It was concluded that 3 courses of adjuvant CMF was not sufficient compared with longer duration of chemotherapy especially in younger women and in patients with estrogen receptor (ER)-negative primaries. Reintroduction chemotherapy showed additional benefits but remains investigational.

1.3 Other chemotherapy regimens used in the adjuvant setting for ER-negative disease: AC or EC x 4 ; AC or EC x 4 followed by CMF x 3; CEF$_{1,8}$ x 6; CAF$_{1,8}$ x 6; FEC$_{100}$ x 6; AC/EC x 4 followed by Taxol/Taxotere x 4 (normal or dose-dense); iv FAC x 6; A x 4 followed by iv CMF x 4; FEC100 x 3 followed by Taxotere x 3; TAC x 6; A x 3 followed by T x 3 followed by classical CMF x 3. The adjuvant treatment consisting of doxorubicin-cyclophosphamide (AC) or epirubicin-cyclophosphamide (EC) for 4 cycles (3) followed by 3 cycles of “oral” CMF, is currently being tested by the International Breast Cancer Study Group (IBCSG) in the context of two different adjuvant trials for pre and postmenopausal patients (Trials 13, 14). Overall, 2266 pre and postmenopausal patients have received AC/EC→CMF as an adjuvant treatment for node-positive breast cancer with and without a period of 16 weeks between the two chemotherapies. Of these, 1214 have received AC/EC→CMF without the gap. No toxic deaths were reported during treatment. Fifty-eight life threatening toxicities have been encountered, mostly neutropenia and leukopenia. In regard to delayed toxicity, it is worthwhile to emphasize that at a median follow-up of approximately 2 years, there have been 13 reported cases of second primary malignancies among the 2266 patients which included colon (2), cervix, lung, endometrial (2), ovarian (3), melanoma (3), and AML (1). There was only one case of leukemia. These data clearly support the feasibility of an adjuvant treatment consisting of 4 cycles of AC followed by 3 cycles of oral CMF, given according to the original schedule. In regard to the activity of this treatment, IBCSG data are premature with a median follow-up time of approximately 2 years. Nevertheless, the first portion of this treatment (4 cycles of AC) represents a well recognized adjuvant treatment for node-positive breast cancer patients, and its activity has been demonstrated to be comparable to 6 cycles of “oral” CMF (3). Therefore, it is reasonable to hypothesize that the addition of 3 cycles of “oral” CMF, given sequentially after the 4 courses of AC/EC, would result in an antitumor activity at least as good as 6 cycles of CMF. The Southwest Oncology Group (SWOG) recently reported results of a phase III study comparing classical CMF with CAF in high-risk node negative patients (4). A small advantage in terms of DFS and OS was observed for the anthracycline containing regimen compared with CMF, although side effects were more frequently observed with the CAF regimen. The

CEF regimen was investigated in premenopausal node-positive patients from the National Cancer Institute – Canada (NCIC) and CEF was superior to CMF in terms of both DFS and OS (5).

The Intergroup Trial 0102 led by the CALGB had a 3 x 2 design to compare 1) three doses of doxorubicin (60, 75 or 90 mg/m2 by random allocation) plus cyclophosphamide (600 mg/m2) given iv on day 1 every three weeks for four courses (AC x 4), and 2) the use of paclitaxel (175 mg/m2 in a 3 hour infusion every three weeks) for four courses following AC with no additional chemotherapy after AC (A). The trial included 3170 patients and its results were reported despite a very short median follow-up of 22 months at the 1998 ASCO Conference. The 18 months DFS was 90% for AC followed by taxol compared with 86% for AC (p=. 0077). Overall survival was 97% for AC plus paclitaxel compared with 95% for AC (p=0.039). Although the absolute differences in early follow up were small, they represented a substantial proportional reduction in the risk of relapse and death. The early appearance of improved outcome seen with taxol was exclusively observed for patients with tumors classified as ER-negative. The recently reported French intergroup study indicated possibly superior results with increased epirubicin dose administered in the FEC (fluorouracil, epirubicin, cyclophosphamide) regimen (B). The 5-year disease-free survival (DFS) was 54.8% with FEC 50 and 66.3% with FEC 100 (P =.03). The authors concluded that after 5 years of follow-up, the increased epirubicin dose led to a significant benefit in terms of DFS, with a high survival rate among patients with poor-prognosis breast cancer.

Since the start of the IBCSG 22-00 Trial, several other regimens have demonstrated significant activity in well-conducted randomized clinical trials and have been approved as induction regimens for this trial: AC/EC x 4 followed by Taxol/Taxotere x 4, (normal or dose-dense); IV FAC x 6; A x 4 followed by IV CMF x 4; AC x 4 followed by P x 4; FEC x 3 followed by Taxotere x 3. An update on currently recommended regimens can be found in the consensus publication from the St. Gallen 2005 conference (C).

The results of the Intergroup phase III Trial BIG 2-98 were recently presented. The primary aim of the study was to compare disease-free survival of an adjuvant treatment with docetaxel given either sequentially (A-T) or in combination with doxorubicin (AT) and followed by CMF to doxorubicin alone (A) or in combination with cyclophosphamide (AC) and followed by CMF in operable breast cancer patients with positive axillary lymph nodes. Sequential A-T-CMF produced superior event-free survival over both combination AT-CMF (p=0.047) and A-CMF (p=0.035). In the sequential A-T-CMF arm febrile neutropenia was observed in 7.5% of the patients with 1% of treatment related death (D).

Inserted References 5A-5D
A. Henderson IC, Berry D, Demetri G, et al. Improved disease-free (DFS) and overall survival (OS) from the addition of sequential paclitaxel (T) but not from the escalation of doxorubicin (A) dose level in the adjuvant chemotherapy of patients (pts) with node-positive primary breast cancer (BC). Proc Am Soc Clin Oncol 1998; 17: 101.

B. The French Adjuvant Study Group. Benefit of a high-dose epirubicin regimen in adjuvant chemotherapy for node-positive breast cancer patients with poor prognostic factors: 5-year follow-up results of French Adjuvant Study Group 05 randomized trial. J Clin Oncol 2001;19(3):602-11.

C: Goldhirsch A, Glick JH, Gelber RD, Coates AS, Thuerlimann B, Senn HJ. Meeting Highlights: International Expert Consensus on the Primary Therapy of Early Breast Cancer 2005. Ann Oncol 16:1569-1583, 2005.

D: Crown JP, Francis P, Di Leo A, Buyse M, Balil A, Anderson M, Nordenskjöld B, Jakesz R, Gutierrez J, Piccart M. Docetaxel given concurrently or sequentially to anthracycline-based adjuvant therapy for patients with node-positive breast cancer, in comparison with non-docetaxel adjuvant therapy: First results of the BIG 2-98 trial at 5 years median follow-up. J Clin Oncol (Meeting Abstracts) 2006 24: LBA519.

1.4 Efficacy of "low-dose CMF" chemotherapy. There is evidence of activity for chemotherapy delivered at low doses for a prolonged time. The EORTC conducted a trial in patients with operable breast cancer, which compared 24 months of low-dose CMF d1,8 with no adjuvant treatment. Four hundred fifty-two patients with axillary node-positive breast cancer who received postoperative irradiation were prospectively randomized. Chemotherapy was given for a period of 2 years and consisted of monthly courses of cyclophosphamide 50 mg/m2 orally on days 1 to 14, methotrexate 15 mg/m2 intravenously on days 1 and 8, and fluorouracil 350 mg/m2 intravenously on days 1 and 8 (CMF). At a median follow-up time of 10 years, the overall survival duration was significantly prolonged in the chemotherapy arm (hazards ratio, 0.75; 95% confidence interval, 0.56 to 0.99; P = .04). Ten-year overall survival rates (+/- SE) were 59% (+/- 3.6%) for the chemotherapy arm and 50% (+/- 3.7%) for the control arm. Time to local relapse was significantly prolonged in the chemotherapy arm (hazards ratio, 0.63; 95% confidence interval, 0.42 to 0.94; P = .02). Patients with one to three positive axillary nodes and patients with estrogen receptor-negative tumors especially benefited from chemotherapy. Therefore, prolonged low-dose adjuvant CMF can significantly prolong overall survival in patients with node-positive breast cancer especially if ER-negative (6).

1.5 Lack of efficacy of continuous CMFVP (SWOG) if given with tamoxifen as compared with tamoxifen <u>in ER+ patients</u>. The SWOG group randomized 1892 postmenopausal patient with ER-positive, node positive breast cancer to 1) tamoxifen 20 mg/day, 2) CMFVP (cyclophosphamide 60 mg/m2/day, fluorouracil 400 mg/m2 weekly for 1 year, vincristine 0.625 mg/m2 weekly for the first 10 weeks, methotrexate 15 mg/m2 weekly for 1 year 3) or the combination of both (7). After a median follow-up duration of 6.5 years no difference in terms of DFS or OS was observed. It must be emphasized that the trial was conducted in a potentially endocrine treatment-responsive population.

1.6 Role of angiogenesis in the metastatic process. Angiogenesis, the process leading to the formation of new blood vessels, plays a central role in tumor progression of solid neoplasia. The switch from the avascular to the vascular phase is generally accompanied by rapid primary tumor growth and local invasiveness (8-13). Furthermore, angiogenesis is also necessary both at the beginning and at the end of the development of distant metastasis and is implicated in the phenomenon of dormant micrometastases (14-16). Tumor cells may induce angiogenesis via release of numerous growth factors, prostaglandins, etc.; and by their attraction of inflammatory cells which in turn release multiple angiogenic stimuli. Inhibitory modulation of many of the individual steps of capillary growth, which occurs following an angiogenic stimulus, can block the angiogenic response. This leads to the expectation that an effective inhibitor of a single key step in this cascade would be able to completely suppress angiogenesis. A number of anti-angiogenic agents have been recently discovered, and some are under early clinical evaluation (17-19). In animal models, treatment with angiogenesis inhibitors has proven anti-tumor effects *in vivo*, and can both reduce metastases and lead to regression of the primary growth by necrosis following capillary retraction (18). In cancer patients anti-angiogenic peptides may be altered in the serum or urine. In particular, the angiogenic protein VEGF (vascular endothelial growth factor) was found abnormally elevated in the serum in more than 70% of breast cancer patients (16).

1.7 Anti-angiogenic activity of cytotoxics. Several common anticancer agents have been shown to have anti-angiogenic activity. Low doses of methotrexate inhibited endothelial cell proliferation *in vitro*, and inhibited neovascularization by endothelial cell growth factor in the rabbit cornea assay (20). Anti-angiogenic activity of cyclophosphamide, methotrexate, anthracyclines, vinca alkaloids, paclitaxel, cisplatin, fluorouracil and interferon has been demonstrated in the chicken allantoic membrane assay (20). In addition, growth of the chick embryo was markedly inhibited by cyclophosphamide, methotrexate, fluorouracil and melphalan. It is therefore possible that conventional cytotoxic agents may exert a tumor suppressive effect through an anti-angiogenic mechanism, in doses that would be tolerable on a long-term basis. The hypothetical clinical benefit from such effect is best tested in patients with

endocrine-unresponsive disease. No interaction is assumed to take place between the antiangiogenic and the endocrine effect of cytotoxics in this setting.

Many investigators have reported that therapy with low-dose methotrexate improves the clinical course of rheumatoid arthritis (RA) patients (21-27). Although several studies both *in vivo* and *in vitro* have demonstrated that methotrexate has the potential to suppress a variety of ongoing immune reactions in rheumatoid inflammation, the underlying mechanism is not clearly understood. Recent reports suggested that methotrexate has an anti-angiogenic effect and may suppress rheumatoid inflammation through the reduction of synovial small blood vessels responsible for mononuclear cell infiltration and proliferation of synovial tissue (26). The drug is generally administered at a weekly dose of 7.25 to 12.5 mg/m2 either alone or in combination with sulphasalazine. Treatment is well tolerated with mild or moderate nausea, and an increase in transaminases or leukopenia (27).

We evaluated the clinical efficacy and impact on serum VEGF levels of low dose oral cyclophosphamide and methotrexate (CM) in patients with metastatic breast cancer (28). Methotrexate was administered 2.5 mg twice a day on days 1, 2 every week and cyclophosphamide 50 mg per day, continuously. Among 57 evaluable patients, we observed 2 complete remissions and 8 partial remissions for an overall response rate of 18% (external peer review confirmed). The majority of the patients were previously exposed to combination chemotherapy containing these compounds administered at "standard" doses. Mean serum log VEGF level decreased by 33% after 2 months (p=0.0007). Moreover, 9 patients had a stabilization of disease for 6 months or more yielding an overall clinical benefit in 33% of the patients.

1.8 Toxicity of CM maintenance. Among 57 evaluable patients with advanced disease, all pretreated with chemotherapy, side effects were generally mild or moderate and included leukopenia (31 cases, 54%), anemia (7 cases, 12%) and altered transaminases (20 cases, 34%; 6 of 20 patients had concomitant liver metastasis). Mild nausea and stomatitis were observed respectively in 13 (23%) and 3 (5%) cases. Grade 3 leukopenia and anemia were observed in 1 patient and grade 3 reversible increase in transaminases was observed in 9 patients (6 with concomitant liver metastasis). Only 1 patient had grade 4 leukopenia.

1.9 Effects of long-term adjuvant chemotherapy. Only a few studies have evaluated the long-term effects of adjuvant chemotherapy for breast cancer. In an Istituto Nazionale Tumori trial, a total of 2,465 patients received CMF-based (6-8 cycles) adjuvant chemotherapy (29). Compared with the general female population, the relative risk for developing leukemia following this CMF-based adjuvant chemotherapy was 1.29 at 15 years of follow-up. In fact, only three patients developed acute non-lymphocytic leukemia (cumulative risk 0.23 (0.15)%; relative risk 2.3). In a case-control study, the risk of leukemia was evaluated in a cohort of 82,700 women from five regions of the United States diagnosed with breast cancer between 1973 and 1985 (30). Acute non-lymphocytic leukemia rates were significantly elevated after regional radiotherapy alone (relative risk, 2.4), alkylating agents alone (relative risk, 10.0), and combined radiation and drug therapy (relative risk, 17.4). Dose-dependent risks were observed after radiotherapy and treatment with melphalan and cyclophosphamide. Melphalan was 10 times more leukemogenic than cyclophosphamide (relative risk, 31.4 vs. 3.1). Some increased risk was associated with total cyclophosphamide doses of less than 20,000 mg. For doses up to 30,000 mg, the relative risk was 4.7 and for doses ≥ 30,000 (median dose 37,850 mg), it was 9.4. For 972 patients evaluated from IBCSG Trials I-III, only one case of leukemia was observed. The patients, who received 12 cycles of CMF (cumulative total cyclophosphamide dose 25,000-33,600 mg), had a median follow-up of 18 years (31). In NSABP Trial B-25, six cases of AML (all M4 or M5 according to FAB classification, 3 out of 4 patients who had cytogenetics had chromosome 11q23 abnormalities) were identified out of the 2548 participants diagnosed 10 to 18 months from start of adjuvant therapy (32). Use of anthracyclines associated with cyclophosphamide and fluorouracil (with or without G-CSF and Bactrim) significantly increased the incidence of acute myelogenous leukemia. In a Canadian trial of

CEF versus CMF, four cases of AML in 351 patients were observed after a median follow-up of only 50 months (33). All 4 cases received CEF together with Septrim®. A Scandinavian trial of high-dose chemotherapy versus dose-escalated tailored FEC therapy resulted in 7 AML/MDS among the 525 patients in the study (34).

On this maintenance chemotherapy trial, a patient receiving 6 cycles of CMF and then continuing on to CM maintenance will receive a total dose of cyclophosphamide ranging between 30,820 and 35,000 mg. Four cycles of AC plus CM maintenance will result in a total dose of cyclophosphamide ranging between 23,000 and 24,000 mg, whereas 4 cycles of AC followed by 3 cycles of CMF and CM maintenance will yield a total dose of 29,300 - 32,400 mg. Low risk of leukemia is anticipated, based upon the dose levels of cyclophosphamide proposed in this maintenance chemotherapy trial.

1.10 Efficacy of adjuvant trastuzumab Adjuvant trastuzumab has been tested in 4 large randomized trials, including more than 13,000 patients with either overexpression or amplification of HER2/*neu*. These trials have reported results based on protocol-defined early stopping. The HERA trial showed a highly significant and substantial improvement in DFS (RR 0.54). A pooled analysis of the NSABP and NCCTG trials showed a similar improvement in disease-free survival (HR 0.48) and significantly improved overall survival (HR 0.67) (34A).

34A. Piccart-Gebhart M: First results of the HERA trial; Romond E et al for the joint analysis of NSABP-B-31 and NCCTG-N9831; Perez E et. Al., for the NCCTG-N9831 collaboration; all presented at ASCO Meeting, Orlando, FL, May 16, 2005.

2 Trial objectives

2.1 To evaluate the efficacy of a low-dose chemotherapy regimen, hypothesized to have anti-angiogenic activity, administered following a standard chemotherapy program, in patients whose tumors are not endocrine therapy-responsive.

2.2 Treatment comparisons will be based upon the following endpoints:
2.2.1 Disease-free survival
2.2.2 Overall survival and systemic disease-free survival
2.2.3 Toxicity
2.2.4 Quality of life

3 Patient selection

3.1 Criteria for patient eligibility

3.1.1 Premenopausal or postmenopausal patients with histologically proven primary breast cancer. See Section 4.6.2 for definitions of pre and postmenopausal status.

3.1.2 The primary tumor must be classified as $T_{1a,b,c}$, T_2, T_3, or pT_4 with minimal dermal invasion, N_X, pN_0, $pSentN_0$, pN_1, or pN_2, and M_0. Patients with disease defined as SNB positive are eligible only if they have undergone an axillary dissection, OR are SNB micrometastatic and are randomized to IBCSG Trial 23-01. The tumor must be confined to the breast, without detected metastases elsewhere.

3.1.3 Estrogen and progesterone receptor status must be known by immunohistochemistry, before randomization, and both must be negative, which is defined as < 10% of the tumor cells positive by

immunohistochemical evaluation. All laboratories are required to register with a quality assurance scheme (See Appendix III). Detailed guidelines for assessments of ER and PgR are given in the Appendix III.

3.1.4 HER2 status must be known and determined by either immunohistochemistry or FISH before randomization.

3.1.5 The induction chemotherapy must be approved (see Table 1) and must begin (or have begun) within 8 weeks of definitive surgery (see Figure 1).

3.1.6 Patients must have had either:

- a total mastectomy. Radiotherapy is optional after mastectomy. OR
- a breast-conserving procedure (lumpectomy, quadrantectomy or partial mastectomy with negative margins) with radiotherapy planned. The local pathologist must give the margins of resection in the pathology report. Radiation therapy to the conserved breast is required.

3.1.7 Patient must have had pathological axillary staging performed on a specimen from axillary clearance or sentinel node biopsy. In order to be classified as pN_0, patients who receive axillary clearance must have a minimum of **6** lymph nodes available for pathological examination. The number of nodes examined should be reported.

3.1.8 Patients must have completed pre-randomization Quality of Life (QL) Forms. The only exceptions are cognitive or physical impairment that interferes with QL assessment or inability to read any of the languages available on IBCSG QL forms.

3.1.9 The patient must be in adequate health to begin or continue with induction chemotherapy or start maintenance chemotherapy:

- WBC greater than $3.0x10^9$/l
- Platelets greater than $100x10^9$/l
- Serum creatinine below 120 µmol/l, adequate liver values
- Serum bilirubin below 20 µmol/l
- No cystitis
- No evidence of any toxicity grade 2 or worse (CTC), in particular: nausea, vomiting, diarrhea, mucositis (stomatitis or any other mucousal inflammation), or epigastric pain.

3.1.10 Written informed consent dated and signed by both patient and investigator (including pathology material consent).

3.1.11 Must be geographically accessible for follow-up.

3.1.12 Patients must be informed of, and agree to, data and material transfer and handling, in accordance with national data protection guidelines and Swiss data protection law (as IBCSG is a foundation under Swiss law).

3.2 Criteria for patient ineligibility

3.2.1 Patients with T_4 carcinoma with ulceration of skin, infiltration of skin (except pathologically minimal dermal involvement), peau d'orange or inflammatory breast cancer, or with distant metastases. Any suspicious manifestation requires appropriate investigation to exclude metastasis.

3.2.2 Patients with bilateral malignancies (except *in situ* carcinoma), or with a suspicious mass in the opposite breast, unless that mass has been proven by biopsy to be benign.

3.2.3 Patients, who, before start of induction chemotherapy, had a skeletal pain of unknown cause, elevated alkaline phosphatase, or a bone scan showing hot spots for which metastases cannot be ruled out by X-ray, MRI and/or CT.

3.2.4 Patients in whom the margins of the resected specimen (whether mastectomy or local excision) contained invasive tumor. Patients who have local excision with a positive margin may become eligible if within **8** weeks from first surgery they undergo adequate resection or mastectomy with clear margins.

3.2.5 Patients with previous or concomitant malignancy EXCEPT adequately treated basal or squamous cell carcinoma of the skin or *in situ* carcinoma of the cervix, or contra- or ipsilateral *in situ* breast carcinoma.

3.2.6 Patients who have received prior therapy for breast cancer other than primary irradiation, approved induction regimen, or trastuzumab. Prior endocrine therapy for breast cancer or prevention is not permitted.

3.2.7 Patients with non-malignant systemic diseases that would prevent them from undergoing any of the treatment options, or would prevent prolonged follow-up.

3.2.8 Patients with psychiatric or addictive disorders that would prevent them from giving informed consent to therapy and randomization.

3.2.9 Patients who either have been pregnant or who have lactated within 6 months of diagnosis.

3.2.10 Patients with a history of noncompliance to medical regimens and patients who are considered potentially unreliable.

4 Stratification and randomization

4.1 When to randomize

Patients must be randomized within the following "Randomization Window" (see Figure 1): after definitive surgery but within 56 days after the first day of the last cycle of induction chemotherapy.

4.2 Registration procedures

1. Verify eligibility.
2. Obtain written informed consent for both the clinical trial and the pathology material submission, signed and dated by the patient and investigator (see section 12.2 and Appendix I).
3. Complete Confirmation of Registration Form (A). The date the Informed Consent Form was signed by the patient and the date it was signed by the investigator are both required to complete randomization.
4. Complete the pre-randomization Quality of Life (QL) core and module forms. (See Section 3.1.6 for exceptions.)

5. Depending on your Group's choice, either:
   - Telephone or fax your Randomization Center to review the eligibility and randomization information. Your Randomization Center will access the IBCSG Registration/Randomization System

   or
   - Directly access the IBCSG Registration/Randomization System

   If you telephone or fax the information, the Randomization Center will provide the Participating Center with the following information, as well as with the name of the person performing the randomization. In the latter case, the Randomization System will provide this information via email.
   - Randomization number (Patient ID)
   - Treatment assignment
   - Date of randomization

6. When the randomization is complete, fill in the Confirmation of Registration Form (A) with the information above and fax or mail the completed A and PMC Forms to the IBCSG DMC.

   This completed A Form is considered the essential document for regulatory purposes.

7. Mail the pre-randomization Quality of Life (QL) Core and Module Forms to the IBCSG DMC.

8. File your copy of the completed Confirmation of Registration Form (A)

4.3 Randomization centers

4.3.1 NHMRC Clinical Trials Centre, University of Sydney,
Locked Bag 77, Camperdown NSW 2050, Australia
Australia: 1-800-027-928
New Zealand & Hong Kong: 00-800-0279-2888
Fax: +61 2 552 3881

4.3.2 FSTRF Randomization Help Desk
Frontier Science & Technology Research Foundation (FSTRF)
4033 Maple RD, Amherst, NY 14226 USA
Phone: +1 716 834 0900 ext. 7301
Fax: +1 716 836 6097
Email: bc.helpdesk@fstrf.org

4.4 Information required at registration

4.4.1 Completed Confirmation of Registration Form (A) for IBCSG Trial 22-00.

4.4.2 Patient's initials.

4.4.3 Patient's date of birth.

4.4.4 Institution center code number.

4.4.5 City/hospital name.

4.4.6 Pathology number of the primary tumor specimen.

4.4.7 Pathology institute where this specimen is available and Pathology Institute Code (if known).

4.4.8 Verification of eligibility. Form A confirms that all eligibility criteria have been met.

4.4.9 Verification that written informed consent (including pathology material consent) has been signed and dated by the patient and investigator.

4.4.10 Verification that protocol-required pathology material is available.

4.4.11 Completed pre-randomization Quality of Life core and module forms and the date forms were completed, or notification on Confirmation of Registration Form that patient has a cognitive or physical impairment that interferes with QL assessment or an inability to read any of the languages in which IBCSG QL forms are available.

4.4.12 Stratification factor: Menopausal status: see section 4.6.2.

4.4.13 Stratification factor: Induction chemotherapy regimen: see section 4.6.3.

4.4.14 Date induction chemotherapy began, or date scheduled to begin.

4.4.15 Date of definitive surgery.

4.4.16 Full name of person randomizing (when randomizing through a Randomization Center).

4.4.17 Fax number where completed Form A should be sent immediately after randomization (when randomizing through a Randomization Center).

4.4.18 Telephone number to be used immediately if there is a problem with randomization (when randomizing through a Randomization Center).

4.4.19 Full name of investigator responsible for confirming eligibility.

4.4.20 Name of investigator responsible for the patient.

4.5 Randomized groups

Randomization to receive one of the two treatment arms:

4.5.1 Induction chemotherapy alone

4.5.2 Induction chemotherapy immediately followed by CM maintenance for one year.

4.6 Stratification

4.6.1 Institution.

4.6.2 Menopausal status (pre [a or c] versus post [b or d])

a) Pre (<6 mo since last menstrual period (LMP) **AND** no prior bilateral ovariectomy **AND** not on estrogen replacement)
b) Post (Prior bilateral ovariectomy **OR** >12 mo since LMP with no prior hysterectomy)
c) Above categories not applicable **AND** age <50 (Pre)
d) Above categories not applicable **AND** age ≥ 50 (Post)

4.6.2.1 Premenopausal a or c.

4.6.2.2 Postmenopausal b or d.

4.6.3 Induction chemotherapy (AC/ECx4 versus all others)

4.6.3.1 AC/EC x 4

4.6.3.2 All others (see Table 1)

5 Treatment details

5.1 Trial drug treatments

5.1.1 Induction Chemotherapy

Patients must receive one of the approved induction chemotherapy regimens listed in Table 1. The start of induction chemotherapy must occur within **8** weeks of the definitive breast cancer surgical procedure.

5.1.2 Radiotherapy

Radiotherapy is optional after mastectomy. Radiotherapy is indicated after breast conserving surgery according to prospectively determined guidelines within each institution. It must be given to the breast and may be given to the draining node areas (axilla, internal mammary chain and supraclavicular irradiation). Radiotherapy should begin after anthracycline containing chemotherapy (i.e., AC/EC, CEF, FEC, CAF) has been completed. Radiation therapy may be given either after all chemotherapy or integrated into chemotherapy (if regimen is considered safe by the investigator). **Radiotherapy must not delay the beginning of the maintenance chemotherapy.**

5.1.3 Anti-angiogenic treatment: CM Maintenance.

CM maintenance must begin after the last day of the last cycle of induction chemotherapy (eg, day 22 of AC or day 29 of CMF), but within 56 days after the first day of the last cycle (see Figure 1); and continue for 365 days.

C=cyclophosphamide 50 mg/day orally continuously for 365 days
M=methotrexate 2.5 mg/twice a day orally days 1 and 2 of every week for one year (52 weeks)

The total time spent receiving CM maintenance should not exceed 365 days (1 year) regardless of treatment delays and modifications.

5.1.3.1 Required patient health status for beginning CM maintenance

To begin CM maintenance the patient must have:

- WBC greater than 3.0×10^9/l
- Platelets greater than 100×10^9/l
- Serum creatinine below 120 µmol/l, adequate liver values
- Serum bilirubin below 20 µmol/l
- No cystitis
- No evidence of any toxicity grade 2 or worse (CTC), in particular: nausea, vomiting, diarrhea, mucositis (stomatitis or any other mucousal inflammation), or epigastric pain.

5.1.4 **Trastuzumab**

Trastuzumab (Herceptin®) may be given to patients with HER2-positive beast cancer during or following induction, and/or during and following CM Maintenance.

5.2 Side effects of study drugs

Grading for all side effects will be according to the NCI Common Toxicity Criteria (CTC), version 2, available at http://ctep.cancer.gov/reporting/CTC-3.html

5.2.1 Dose Modifications: Induction Chemotherapy

5.2.1.1 **Hematological toxicity**: Standard International Breast Cancer Study Group modifications of the approved induction regimens will be used. Doses of cyclophosphamide, methotrexate, 5-fluorouracil, and doxorubicin or epirubicin will be administered according to the following guidelines, based on blood counts performed on the day of treatment administration (both values must be available):

Percentage of full dosage to be given based on platelets and WBC

Platelets	WBC >3.0 x 10^9/l	WBC 2.0-3.0 x 10^9/l	WBC <2.0 x 10^9/l
≥ 75 x 10^9/l	100%	75%	Delay one week then treat according to scale. If WBC still below 2.0, omit treatment for that week.
50 – 75 x 10^9/l	75%	Delay one week then treat according to scale. If platelets still 50 – 75 x 10^9/l and WBC between 2.0 and 3.0, omit treatment for that week	Delay one week, then treat according to scale. If WBC still below 2.0 or platelets 50 – 75 x 10^9/l, omit treatment for that week.
< 50	delay 1 week	delay 1 week	delay 1 week

Do not reduce dose for grade 3 hematologic toxicity noted during the course of treatment not associated with morbidity. For grade 3 toxicity associated with morbidity or grade 4 toxicity, dose may be reduced by 25%.

No primary prophylactic administration of G-CSF (from the first cycle) is permitted. In selected regimens (e.g. A x 3 followed by T x 3 followed by classical CMF x 3) if delay > 7 days for neutropenia or neutropenia (ANC < 1.0 x 109/L) and NCI grade 2 oral, or fever (>38°C) or G3-G4 infective episode during the previous cycle, next cycles can be delivered with G-CSF (150 µg/m^2 subcutaneously, day 4 to day 12 (inclusive) for doxorubicin or docetaxel containing regimens and day 16 to day 22 for CMF).

You may reduce subsequent doses by 25% for lowest (nadir) WBC< 1.0 x 109/l without toxicity or WBC 1.0 x 109/l - 1.5 x 109/l with concomitant fever ≥38oC requiring antibiotics.

5.2.1.2 **Peripheral neuropathy:** In case of grade 2 toxicity, taxol/taxotere should be administered at 75% of the scheduled dose. In the case of grade 3 or 4, treatment should be interrupted.

5.2.1.3 **Anaphylactoid type reactions, hypersensitivity reactions:** In the case of hypersensitivity reactions despite premedication, it is likely that the reaction will occur within a few minutes of the start of the second infusion of paclitaxel or docetaxel. Therefore, the infusion must be given drop by drop for the first 5 minutes, and a careful evaluation of the patient is required.

In addition, follow the guidelines listed below:

Mild symptoms:
For localized cutaneous reaction, such as pruritus, flushing, rash: decrease the rate of infusion until recovery from symptoms, stay at bedside, and then complete drug infusion at the initial planned rate. At subsequent cycles use the same standard premedication.

Moderate symptoms:
For any symptom not listed above (mild) or below (severe), such as generalized pruritus, flushing, rash, dyspnea, hypotension with systolic BP > 80 mm Hg.: stop taxane infusion; administer i.v. dexamethasone 10 mg (or equivalent) and i.v. diphehydramine 50 mg (or equivalent). Resume taxane infusion after recovery of symptoms. In case of no other symptoms, give i.v. dexamethasone 20 mg (or equivalent) and i.v. diphehydramine 50 mg (or equivalent) one hour before infusion, in addition to the standard premedication at subsequent cycles. IF A REACTION RECURS PATIENT SHOULD GO OFF CHEMOTHERAPY.

Severe symptoms:
For bronchospasm, generalized urticaria, hypotension with systolic BP less than or equal to 80 mm Hg., angioedema: stop taxane infusion; administer i.v. dexamethasone 10 mg (or equivalent) and i.v. diphehydramine 50 mg (or equivalent), add epinephrine as needed. If possible, reinfuse the patient within 72 hours using standard premedication: i.v. dexamethasone 20 mg (or equivalent) and i.v. diphehydramine 50 mg (or equivalent) one hour before infusion. At the subsequent cycles, dexamethasone (or equivalent) should be given at 20 mg orally the evening before chemotherapy, the morning of chemotherapy, and one hour before taxanes infusion. Additionally diphenhydramine (or equivalent) should be given at 50 mg i.v. 1 hour before taxane infusion. IF A SEVERE REACTION RECURS PATIENT SHOULD GO OFF CHEMOTHERAPY.

Anaphylaxis (NCI CTC grade 4 reaction):
NO FURTHER INDUCTION CHEMOTHERAPY.

5.2.2 Dose modifications: CM maintenance

To achieve a 50% reduction of CM maintenance, give cyclophosphamide every other day and methotrexate one tablet on day 1 and one on day 2. Dose modifications and treatment delays do not alter the total one year (365 days) duration of CM maintenance.

5.2.2.1 **Hematologic toxicity:** Doses of cyclophosphamide and methotrexate will be administered according to the following guidelines, based on blood counts performed on day 1 every calendar month:

Percentage of full dosage to be given based on platelets and WBC (both values must be available)

Platelets	WBC >3.0 x 10^9/l	2.0-3.0 x 10^9/l	< 2.0 x 10^9/l
100 x 10^9/l	100 %	50 %	0 %
75-99 x 10^9/l	50 %	50 %	0 %
< 75 x 10^9/l	0 %	0 %	0 %

Any grade 3 hematological toxicity noted during the course of treatment should be managed by a 50% dose reduction (as described in 5.2.2) after hematological recovery. Re-escalation of drug doses should only be attempted if close monitoring is possible.

5.2.2.2 **Renal dysfunction at the time of drug administration:** Methotrexate and cyclophosphamide should be administered only in the presence of normal renal function (serum creatinine < 120µmol/l). Serum creatinine should be monitored every month. Special caution should be employed with drugs such as salicylates or sulphonamides, which may delay methotrexate excretion. Early onset of stomatitis, diarrhea or marrow depression should be treated as a potential emergency, and may require correction of renal function and/or administration of folinic acid (leucovorin).

5.2.2.3 **Hematuria**: All patients should be instructed as to the importance of high fluid intake during cyclophosphamide therapy. If a grade 2, 3, or 4 hematuria occurs despite hydration, cyclophosphamide treatment should be stopped until recovery and restarted at 50% of the dose of cyclophosphamide.

5.2.2.4 **Gastrointestinal toxicity**: In the event of grade 3 or 4 anorexia, nausea, vomiting, diarrhea, stomatitis, dryness of the mouth or epigastric pain, all therapy should be postponed until symptoms subside. In the event of debilitating vomiting or diarrhea, a 50% reduction of CM is recommended for the next month, with subsequent re-escalation to full dosage if tolerated. If mucosal **ulceration** occurs, no cyclophosphamide or methotrexate should be given for the remainder of the month. Following an occurrence of mucosal ulceration, 50% of the methotrexate and cyclophosphamide dosage is recommended, with re-escalation if tolerated.

5.2.2.5 **Other toxicities**: If considered necessary, dosage may be reduced for other toxicities. Full details of the toxicity and the dosages administered must be recorded on Forms CMM and AE.

5.2.2.6 **Any grade 4 toxicity** other than hematological should be managed by a 50% reduction of dosage with a return to full dosage when full recovery has occurred.

6 End points and definitions of treatment failure

6.1 Trial end points

6.1.1 **Primary end point:** First confirmation of recurrence (local, regional or distant) or metastatic disease, second primary (non-breast) tumor, and/or death.

Disease-free survival (DFS) is defined as the time from randomization to local, regional or distant recurrence (including recurrence restricted to the breast after breast conserving treatment), appearance of a second primary tumor, or death from any cause, whichever occurs first. An *in situ* recurrence either in the ipsilateral or in the contralateral breast is not considered a recurrence (but must be reported on the Follow-Up Form).

6.1.2 **Secondary end points:** Overall survival (OS) is defined as the time from randomization to death from any cause.

Systemic relapse is defined as any recurrent or metastatic disease in sites other than the local mastectomy scar/chest wall/skin, the ipsilateral breast in case of breast conservation, or the contralateral breast.

Systemic disease-free survival (SDFS) is defined as the time from randomization to systemic relapse, appearance of second (non-breast) primary tumor, or death, whichever occurs first.

Quality of Life.

Sites of first recurrence.

Incidence of second (non-breast) malignancies.

Causes of deaths without relapse of breast cancer.

Toxicity will be graded using the NCI Common Toxicity Criteria (CTC) and reported on the CMI and AE Forms.

6.2 Diagnosis of treatment failure

The diagnosis of first treatment failure depends on evidence of recurrent disease which can be classified as either suspicious or acceptable. In either case, this should be specified and reported. Acceptable evidence of treatment failure according to site is defined below. Any events not included in this section are considered unacceptable as evidence of recurrent disease.. Treatment failures include: local, regional, contralateral breast, and distant failures, second (non-breast) primaries, and deaths without recurrence. The date of treatment failure is the time of first appearance of a suspicious lesion, later proven to be a definitive recurrence or metastasis. All events described below should be recorded on the Follow-up Form (E).

6.2.1 **Local failure**

Local failure is defined as a tumor recurrence in any soft tissue of the ipsilateral conserved breast or the chest wall, mastectomy scar, and/or skin.
Acceptable for recurrence in ipsilateral conserved breast:
positive cytology or histology.
Acceptable for recurrence in chest wall, mastectomy scar, and/or skin:
positive cytology or histology or evidence of new lesions (by CT or MRI) without any obvious benign etiology.
Suspicious: a visible or palpable lesion.

6.2.1.1 Treatment after local relapse for patients who received breast-conserving surgery.

Patients may continue to receive the protocol treatment after a relapse in the ipsilateral conserved breast. Continued treatment is only allowed when there is no evidence of loco-

regional disease outside the breast or of distant disease at the time of breast relapse. Details of the local treatment for the conserved breast relapse must be recorded on the Follow-up Form (E). Patients who develop a local relapse other than a relapse in the ipsilateral conserved breast should change therapy.

6.2.2 Regional failure

Regional failure is defined as a tumor recurrence in the soft tissue of the ipsilateral axillary lymph nodes, extranodal soft tissue of the ipsilateral axilla, and/or ipsilateral internal mammary. Regional failure does not include supraclavicular lymph nodes or tumor in the opposite breast.

Acceptable: positive cytology or histology or evidence of new lesions by CT or MRI without a benign etiology.
Suspicious: a visible or palpable lesion.

6.2.3 Contralateral breast failure

Acceptable: positive cytology or histology.
Suspicious: a visible or palpable lesion, suspicious mammogram, ultrasound, or MRI.

6.2.4 Distant failure

Tumors in all areas other than those defined above are considered distant metastases. The following criteria apply:

6.2.4.1 Bone marrow

Acceptable: positive cytology or histology.
Suspicious: unexplained depression of peripheral blood counts and/or a leucoerythroblastic blood picture.

6.2.4.2 Lung

Acceptable: positive cytology or histology or a positive CT or MRI without obvious benign etiology or evidence of progressive disease. (Progressive disease is confirmed by two X-rays with the second showing worsening disease.)
Suspicious: new radiological lesion(s).

6.2.4.3 Pleura

Acceptable: positive cytology or histology.
Suspicious: new pleural effusion.

6.2.4.4 Bone

Acceptable: positive cytology or histology or a positive X-ray, MRI, or CT, one bone scan with new multiple lesions and no obvious benign etiology.
Suspicious: skeletal symptoms or positive scan showing only one new lesion (until confirmed by other imaging study).

6.2.4.5 Liver

Acceptable: positive cytology or histology, or positive CT or MRI without an obvious benign etiology, or evidence of progressive disease by ultrasound. (Progressive disease in this case is confirmed by two ultrasounds with the second showing worsening disease.)
Suspicious: any two of the following: hepatomegaly on physical examination, equivocal ultrasound and abnormal liver function test.

6.2.4.6 Central nervous system

Acceptable: positive cytology or histology. Positive MRI or CT when the clinical picture is suspicious.
Suspicious: any other clinical findings suggestive of this diagnosis.

6.2.4.7 Distant lymph nodes, including ipsilateral supraclavicular lymph nodes
Acceptable: positive cytology or histology, or enlarged lymph nodes in CT or MRI, or progressive disease by physical exam without an obvious benign etiology.
Suspicious: evidence of enlarged lymph nodes by physical exam.

6.2.4.8 Other sites
Acceptable: positive cytology or histology or evidence of progressive disease if only indirect means of diagnosis were used (e.g., X-ray).
Suspicious: clinical and radiological evidence of a tumor.

6.2.5 Second (non-breast) primary

Any positive diagnosis of a second (non-breast) primary other than basal cell or squamous cell carcinoma of the skin, breast carcinoma *in situ* either ipsilateral or contralateral, or cervical carcinoma *in situ* is considered a treatment failure.

6.2.6 Death without recurrence

Any death related to causes other than breast cancer or second (non-breast) primary is considered a treatment failure.

6.2.7 Other noteworthy events

The following events should be recorded on the Follow-up Form (E). These events are NOT considered treatment failures, but must be recorded.

- ipsilateral and contralateral breast cancer *in situ*
- cervical carcinoma *in situ*
- basal or squamous cell carcinoma of the skin

7 Study parameters

7.1 Tables of study parameters

7.1.1 Observation Group

Day 0=date of first dose of induction CT (for follow-up)	Prior to random -ization	Prior to induction CT[1]	AC/EC x 4;	Other induction regimens		Years 1 -5	After year 5
Day of cycle		0	1	1	8		
Frequency						Every 6 months	Every 12 months
Informed consent	x						
Pathology Material Consent	x						
History		x					
Physical examination		x	x	x	x	x	x
Induction toxicities[3]			x	x	x		
Late adverse events[5]						x	x
**Laboratory tests**							
Hematology[6]	x	x	x	x	x	x	x
Blood chemistry[7]	x	x				x	x
Urinalysis[8]							
ECG [9]	√	√	√	√		√	√
LVEF[9a]	√	√	√	√		√	√
**Tumor Evaluations**							
Mammogram[10]		x	√	√	√	x (yearly)	x
Chest-X-ray[11] (PA and lateral views)		x	√	√	√	√	√
Bone scan [12]		√	√	√	√	√	√
Abdominal US, CT[13]		√	√	√	√	√	√
**CRFs**							
Quality of Life[14]	x		First day of last induction cycle			x (mos. 9, 12, 18, 24, ~~36, 48, 60~~)	~~x (month 72)~~
Forms PMC, B,C,F,P Path report, blocks, slides	x						
Form CMI	At completion of induction chemotherapy						
Form E	Every 6 months for the first 5 years and yearly thereafter, using date induction began as day 0						
Form R	At completion of radiotherapy						
Form TC	At trial treatment completion						

x = mandatory
√ = if medically indicated

7.1.2 CM Maintenance Group

Day 0=date of first dose of induction CT (for follow-up)	Prior to randomization	Prior to induction CT[1]	AC/ EC x 4	Other induction regimens		CM Maint[2] arm only		Years 1 -5	After year 5
						prior to starting CM Maint[2]	end of each of 12 months of CM		
Day of cycle		0	1	1	8				
Frequency						once	Every month	Every 6 months	Every 12 months
Informed consent	x								
Pathology Material Consent	x								
History		x							
Physical examination		x	x	x	x	x	x	x	x
Induction toxicities[3]			x	x	x				
CM Toxicities[4]							x		
Late adverse events[5]								x	x
**Laboratory tests**									
Hematology[6]	x	x	x	x	x	x	x	x	x
Blood chemistry[7]	x	x				x	x	x	x
Urinalysis[8]						x	x (every 3 months)		
ECG [9]	√	√	√	√		√	√	√	√
LVEF[9a]	√	√	√	√		√	√	√	√
**Tumor Evaluations**									
Mammogram[10]		x	√	√	√			x (yearly)	x
Chest-X-ray[11] (PA and lateral views)		x	√	√	√		√	√	√
Bone scan [12]		√	√	√	√		√	√	√
Abdominal US, CT[13]		√	√	√	√		√	√	√
**CRFs**									
Quality of Life[14]	x		First day of last induction cycle					x (mos. 9, 12, 18, 24, ~~36, 48, 60~~)	~~x (month 72)~~
Forms PMC, B,C,F,P Path report, blocks, slides	x								
Form CMI	At completion of induction chemotherapy								
Form CMM	Complete monthly and submit form every 3 months								
Form E	Every 6 months for the first 5 years and yearly thereafter, using date induction began as day 0								
Form R	At completion of radiotherapy								
Form TC	At trial treatment completion								

x = mandatory √ = if medically indicated

Legend to Table 7.1

1. The first day of induction chemotherapy is considered Day 0 for the purpose of follow-up. Information collected on the B, C, F, and P forms should reflect the patient's status before the start of induction chemotherapy, even if randomization is after the start of induction.

2. CM maintenance must begin after the last day of the last cycle of induction chemotherapy (eg, day 22 of AC or day 29 of CMF), but within 56 days after the first day of the last cycle (See Figure 1).

3. Severe (grade 3) or higher occurrence of the following toxicities must be recorded in the induction CT Form CMI at any time during induction chemotherapy.
   - Vomiting
   - Nausea
   - Diarrhea
   - Stomatitis
   - Alopecia (grade 2)
   - Cardiovascular toxicity
   - Renal/genitourinary toxicity
   - Neurologic toxicity
   - Other , unexpected

   For hematologic toxicities, record the lowest value of WBC, platelets, absolute neutrophils, and HgB during the entire induction period and at the completion of induction chemotherapy.

4. Any ≥ Grade 3 adverse events during CM Maintenance must be reported on Form CMM and Adverse Event Form AE.

5. Any ≥ Grade 3 late adverse events (toxicities/adverse events occurring after trial treatment is completed) should be recorded on Follow-up Form E and Adverse Event Form AE.

6. Hematology (WBC with neutrophils, platelets, hemoglobin) must be done within 4 weeks prior to chemotherapy and at any time if medically indicated. Hematology should be performed on days 1 (except month one, when CBC from prior to randomization can be used) and 8 (plus visit) of all CMF, CEF, CAF cycles. Hematology should be also performed on day 1 of each cycle of AC/EC, and at each administration of taxanes. Hematology must be done before the start of and every month during oral CM Maintenance.

7. Blood chemistry (AST, ALT, bilirubin, alkaline phosphatase, serum creatinine, serum calcium, BUN (optional)) must be done within 4 weeks prior to surgery, every 3 months during induction chemotherapy, before the start of and monthly during CM chemotherapy and at any other time if medically indicated.

8. Urinalysis should be done before the start of and every 3 months during CM Maintenance.

9. An ECG must be done at any time if medically indicated.

9a. An LVEF evaluation should be done whenever medically indicated.

Radiological assessments

10. A bilateral mammography must be done prior to surgery. A mammography of the conserved and contralateral breast is required at yearly intervals, beginning at one year post diagnosis, and at any other time it is medically indicated.

11. A chest X-ray must be performed prior to surgery and at any other time it is medically indicated. Both PA view and lateral view should be done.

12. A bone scan must be taken before the start of induction chemotherapy and during treatment **only** if alkaline phosphatase is ≥ 2 x normal or if medically indicated otherwise (i.e. bone pain). If the bone scan shows areas suspicious for tumor then these areas should be confirmed by X-ray, CT or MRI.

13. Abdominal ultrasound or abdominal CT is required before the start of induction chemotherapy and **only** if ALT/AST or alkaline phosphatase are ≥ 2 x normal, or if medically indicated otherwise.

Other procedures

14. Both the Quality-of-Life (QL) core form and the Trial 22-00 QL module should be completed before randomization. For those patients randomized before the first day of the last cycle of induction chemotherapy, an additional QL core form plus module are required on the first day of the last cycle of induction chemotherapy. Regardless of randomized treatment assignment or timing of randomization, QL core forms plus modules are required at months 9, 12, 18 and 24~~; and QL core forms are required at months 36, 48, 60 and 72 at the follow-up visits~~ (see Section 10). All patients, regardless of disease status, are to be assessed on the same schedule. If a patient does not complete a scheduled QL assessment, a Missing Quality of Life Assessment Form (MQL) must be submitted for that assessment timepoint.

7.2 Adverse Event Reporting

The main criterion for tolerability is the occurrence of toxicities and adverse events. The severity and causality will be classified according to the NCI Common Toxicity Criteria (CTC). The CTC should be labeled: CTC Version 2.0. The CTC is available for downloading on the internet at (http://ctep.cancer.gov/reporting/CTC-3.html).

The toxicity severity grade provides a qualitative assessment of the extent or intensity of an adverse event, as determined by the investigator or as reported by the subject. The severity grade does not reflect the clinical seriousness of the event, only the degree or extent of the affliction or occurrence (e.g. severe nausea, mild seizure), and does not reflect the relationship to study drug.

Effective with Amendment 4, Grade 1 and Grade 2 adverse events are no longer reported.

Severity grade for other adverse events, not covered in the toxicity grading scale:

~~1 = Grade 1~~	~~mild~~
~~2 = Grade 2~~	~~moderate~~
3 = Grade 3	severe
4 = Grade 4	life-threatening
5 = Grade 5	fatal

7.3 Serious Adverse Event (SAE) Reporting

7.3.1 Definition

A serious adverse event is defined in general as any undesirable medical occurrence/adverse drug experience that occurs during or within 4 weeks after stopping study treatment that, at any dose, results in any of the following:

- is fatal (any cause)
- is life-threatening

- requires or prolongs inpatient hospitalization
- results in persistent or significant disability/incapacity or
- is an unexpected grade 4 toxicity
- is a congenital anomaly or birth defect
- is a secondary cancer
- requires significant medical intervention

Other significant/important medical events which may jeopardize the patient, or may require significant medical intervention to prevent one of the other serious outcomes listed above, are also considered a serious adverse event.

7.3.2 Exceptions to the definition

Any death or serious adverse event that occurs more than 4 weeks after stopping study treatment but is considered to be at least possibly related to previous study treatment is also considered an SAE. All serious adverse events must also be reported for the period in which the study protocol interferes with the standard medical treatment given to a patient. Cases of second primaries are to be regarded as SAEs, regardless of whether they occur during or after study treatment.

Events not considered to be serious adverse events are hospitalizations occurring under the following circumstances:

- elective surgery (planned before entry into the clinical study);
- occur on an outpatient basis and do not result in admission;
- are part of the normal treatment or monitoring of the studied treatment;
- progression of disease.

7.3.3 Reporting SAEs

Any serious adverse event occurring in a patient after providing informed consent must be reported. Information about all serious adverse events will be collected and recorded on the IBCSG Serious Adverse Event Report Form (SAE Form).

To ensure patient safety, IBCSG must learn of each serious adverse event using the procedures described below:

- The investigator/MD responsible for the patient must fax a signed SAE Form in English within 24 hours to the IBCSG Coordinating Center in Bern.

- Follow-up information should be completed on the original SAE Form within 15 days of the initial report and must be mailed to the following address of the IBCSG Coordinating Center.

IBCSG Coordinating Center
Effingerstrasse 40
CH-3008 Bern, Switzerland

FAX: + 41 31 389 9392
TEL: + 41 31 389 9391

The original Serious Adverse Event Form and the fax confirmation sheet must be kept with the case report forms at the participating center.

IBCSG Coordinating Center will medically review all SAEs with respect to seriousness, causality and expectedness. The Safety Office will prepare and distribute notifications of those SAEs subject to expedited reporting (suspected, unexpected serious adverse reactions, SUSARs), to the appropriate persons and regulatory authorities.

The IBCSG Coordinating Center will record the SAE and prepare a summary report of all SAEs received at the end of each month. Principal Investigators may receive the summary report by mail or view it on the IBCSG web site (www.ibcsg.org).

8 Data collection and submission

8.1 Case report forms schedule

We will conduct the trial according to the ICH Good Clinical Practice (GCP). Keeping accurate and consistent records is essential to a cooperative study. The following forms are to be submitted at the indicated times by the participating institutions for each patient:

IC	Written Informed Consent Form	Obtain before randomization, signed and dated by the patient and investigator. Do not submit, but retain with the patient record.
PMC	Pathology Material Consent	Obtain before randomization, signed and dated by the patient and investigator. Do not submit, but retain with the patient record.
Form QL	Quality of Life	QL core and module forms are required before randomization. If randomized before the first day of the last cycle of induction therapy, additional QL core and module forms are due on day 1 of the last cycle. QL core and module forms are required at months 9, 12, 18 and 24, using the date induction chemotherapy began as day 0; ~~thereafter, QL core forms are due at months 36, 48, 60 and 72.~~<br>If a patient does not complete a scheduled QL assessment, a Missing Quality of Life Assessment Form (MQL) **must** be submitted for that assessment time point.<br>QL and E forms submission schedules are the same for months in which both QL and E forms are required.
Form A (2 pages)	Confirmation of Registration (No. 2000-A (r4))	Fill in before contacting your Randomization Center or entering the IBCSG Registration/Randomization system to randomize the patient. Mail or fax form immediately after randomization.
Form PMC (1 page)	Pathology Material Consent Form (No. 2000-PMC)	Submit within 1 month of randomization.
Form B (3 pages)	Clinical Form (No. 2000-B (r2))	Submit within 1 month of randomization, but should reflect the patient's status before the start of induction CT.
Form C (1 page)	Surgery (No. 2000-C (r1))	Submit within 1 month of randomization, but should reflect the patient's status before the start of induction CT.

Form F (1 page)	Hormone Receptor Form (No. 2000-F (r1))	Submit within 1 month of randomization, but should reflect the patient's status before the start of induction CT, and again if a hormone receptor analysis is done at first recurrence (see Appendix III).
Form P (2 pages)	Pathology Form (No. 2000-P (r1))	Submit within 3 months of randomization with a copy of the original path report and required pathology materials (see Appendix IV).
Form CMI (3 pages)	Induction Chemotherapy Summary Form (No. 2000-CMI (r4)	Submit after completion of adjuvant induction chemotherapy even if patient has recurred.
Form R (1 page)	Radiotherapy Form (No. 2000-R (r2))	Submit for any patient for whom radiotherapy was given or initially planned (even if it was not given).
Form CMM (1 page)	CM Maintenance Form (No. 2000-CMM (r2))	Complete monthly and submit every 3 months for 12 months for patients on CM Maintenance only.
Form AE (4 pages)	Adverse Events Form (No. 2000-AE (r3))	Submit with Form CMM to report $\geq$ Grade 3 adverse events during CM Maintenance and/or with Form E to report $\geq$ Grade 3 long-term adverse events.
Form E (4 pages)	Follow-Up Form (No. 2000-E (r3)	Complete every 6 months for the first 5 years, using the date induction began as day 0, and yearly thereafter. E and QL forms submission schedules are the same for months in which both E and QL forms are required.
Form TC (1 page)	Trial Treatment Completion Form (No. 2000-TC (r2))	Submit when the patient has completed all trial treatment.
Form SAE (2 pages)	Serious Adverse Event Form	See Section 7.3.2
Autopsy Report		Submit whenever available.

8.1.1 Signing and submitting forms

The baseline forms must be signed by the physician responsible for checking eligibility and obtaining informed consent. The Pathology Form (P) must be signed by the pathologist who reviewed the case or the Principal Investigator. All other forms may be completed and signed by the Principal Investigator or by a designee.

Forms should be submitted as outlined in the table above. The Confirmation of Registration (A) Form is a single part form. After completing the form, mail or fax the original and keep a copy on file. All other forms are 2-part forms. The bottom (pink) copy should be removed and kept on file at the participating institution. The two remaining copies (the top white copy and the middle yellow copy) should be sent to:

IBCSG Data Management Center/FSTRF
4033 Maple Road
Amherst, New York 14226 USA
Tel: +1 716 898-7500
Fax: +1 716 836 6097

8.2 Pathology Materials Submission

The following material must be sent to the Coordinating Center within one month of randomization: 1 tumor tissue block, 1 normal tissue block, 1 representative H&E slide from each of the blocks (see Appendix IV).

8.3 Data management

Data collected in this trial will be sent to the Data Management Center of the IBCSG in Amherst, NY, USA. The Data Management Center will process the data and will generate queries and forms requests. The IBCSG Statistical Center in Boston, MA, USA will perform the data analysis.

9 Statistical considerations

9.1 Study design, objectives, and stratification

This protocol is designed to determine if maintenance chemotherapy with low dose cyclophosphamide and methotrexate given for 12 months following 3 to 6 months of induction cytotoxic chemotherapy improves outcome compared with induction chemotherapy alone. The maintenance chemotherapy (CM) regimen has demonstrated activity in metastatic breast cancer and an interference with angiogenic serum factors; it is hypothesized that this regimen will delay tumor recurrence. All patients with hormone receptor-negative tumors (ER-negative and PgR-negative) are eligible. The randomization is stratified according to menopausal status (peri/premenopausal versus postmenopausal), institution, and duration of induction chemotherapy.

9.2 Data analyses

The outcome measures for efficacy comparisons are disease-free survival, systemic disease-free survival, and overall survival as described in Section 6. Kaplan-Meier estimates of the distribution of these measures will be calculated for each of the 2 treatment arms in the study (35).

Other factors will be used to characterize the patients enrolled in the study and to provide descriptive statistics of outcomes according to subgroups of the population. These factors include timing of randomization with respect to start of induction chemotherapy, nodal status, age at randomization, menopausal status, type of induction chemotherapy, timing of start of induction chemotherapy, type of surgery, tumor size, tumor grade, steroid hormone receptor-absent versus steroid hormone receptor-low proportional score, c-erbB-2 (HER2) (see 11.3) score and quality-of-life scores. Cox proportional hazard regression models will be used to investigate whether the primary treatment comparisons are modified by adjustments for various covariates (36). These analyses will be considered secondary and descriptive.

The primary analyses will be intent to treat. Analyses will be conducted on all randomized patients to provide overall estimates of five-year rates for the entire population.

9.3 Sample size considerations

Sample size calculations were carried out using the statistical package EaSt (Cytel Software Corporation, Cambridge, Massachusetts), which provides study designs that incorporate early stopping criteria for interim monitoring. Based on results from ER-negative, node-negative cohorts in Trial V (75% 5-year DFS) and from ER-negative node-positive cohorts in Trial 13-93 (65% 5-year DFS), we assume that the overall five-year disease-free survival for the group receiving induction chemotherapy alone will be 70%. A total of 256 events is required to detect an improvement in five-year disease-free survival to 77.9% (hazards ratio = 0.70) with 80% power using a two-sided 0.05 level logrank test. This accounts for two interim and one final analysis during the conduct of the study. With an accrual rate of 170 evaluable patients per year, 3.5 years of accrual and five years of additional follow-up will be sufficient to obtain

the desired power. To account for non-evaluable cases (5%), the target accrual should be 180 patients per year **(900 total accrual over five years).**

The actual accrual for IBCSG Trial 22-00 has been slower than originally anticipated. As of 31Jul05, 353 patients had been enrolled. With the current accrual rate of 156 patients/year, recruitment will be completed in early 2009. In September 2005, the IBCSG Data and Safety Monitoring Committee recommended that the study continue as planned because the study question remains scientifically important and continuation of recruitment does not pose a risk to patient safety. Although the completion of the study will take longer than planned, the interim and final analyses remain event-driven as described above.

As of June, 2010, 57 (14.3%) of the 398 patients who were randomized to receive CM and had treatment information available did not start CM, most (45) due to patient refusal. This degree of non-adherence reduces the power of the primary intent-to-treat analysis designed to detect a hazard ratio of 0.70 based on 256 events from 80% to 64%. To recover half of the statistical power lost due to non-adherence, amendment 5 increases the target number of events to 307 and increases the target sample size to 1080. This revision increases the power to 72% to detect a true hazard ratio of 0.70 (corresponding to a hazard ratio of 0.745 which is observable with 15% non--adherence). If IBCSG opens a new study enrolling patients with estrogen receptor-negative tumors, we will consider stopping accrual prior to reaching the new accrual goal.

9.4 Accrual estimates, time frame for analyses and interim monitoring

In order to assure the continued safety of the trial, interim analyses will be performed to allow early termination of accrual to the study. Two interim analyses are planned prior to reaching five years of median follow-up. The target number of events for the study is 307, so interim analyses will be planned after 133 (presented to DSMC in April 2009) events and after 215 (70%) events have been observed. Each of the two main analyses will be conducted in coded fashion to determine if sufficient evidence exists to modify the protocol on the basis of observed differences in disease-free survival (DFS). The sequential boundary used is based on an O'Brien-Fleming (37) type use function, as described by Kim and Tsiatis (38). The p-values to be used at the 3 analysis times for the two-sided logrank tests are 0.001, 0.015, and 0.045. No adjustments will be made for multiple comparisons.

During the conduct of this trial, it is possible that more potent and/or less toxic anti-angiogenic regimens than the "low-dose" oral CM regimen may be developed. The IBCSG may consider changing the approach to anti-angiogenic maintenance while the study is ongoing. If this is done, treatment comparisons will be conducted separately within each of the initial and subsequent patient cohorts, and the results will be combined in a stratified analysis.

Additional patient populations may also be identified as eligible for this trial when current IBCSG trials complete accrual. These additional patient cohorts can be added to the randomization as separate strata. This will increase the strength of the treatment comparisons.

Note: This study is designed to achieve 80% power to detect a 30% reduction in the risk of relapse with CM maintenance. Enrollment of 180 patients per year for five years with 3.5 additional years of follow-up will provide 62% power to detect a 25% reduction in risk of relapse (5-year DFS increased from 70% to 76.5%). If the study is designed to achieve 80% power to detect a 25% reduction in risk, then a total of 1,330 patients will be needed (266 per year for each of 5 years plus 3.5 additional years of follow-up).

9.5 Data and Safety Monitoring Committee (DSMC)

The study will be presented for review by the IBCSG Data and Safety Monitoring Committee (DSMC) at each of their semi-annual meetings. Accrual, toxicity, events, and deaths will be monitored. Analyses of

efficacy according to randomization group will be presented only at the timepoints specified for interim analysis to enable early stopping and reporting of the study if large differences in the approaches are observed. The DSMC will also make recommendations concerning potential modifications to the design criteria for this study if the assumptions used in the design are found to be inaccurate.

10 Quality of Life

10.1 Introduction

Patients receiving low dose cytotoxics as anti-angiogenesis treatment following induction chemotherapy are expected to experience additional toxicity and restrictions in their daily life. Quality of life (QL) evaluation is therefore an essential objective in the IBCSG Trial 22-00.

The three objectives of a QL assessment in this protocol are: (i) to compare treatments in regard to QL (ii) to describe QL in regard to disease- and treatment-related burden; (iii) to compare QL in this trial to that in other adjuvant breast cancer trials.

Given that the patients in the experimental arm are to undergo cytotoxic therapy for an additional 12 months, they may possibly adapt to their disease and treatment differently than those in the standard arm. A further objective in this trial is therefore to investigate this process and to clarify whether it is relevant for treatment comparison.

These objectives will be addressed using the same approach to QL assessment that the IBCSG has used since 1986 (39, 40). It has been shown to be feasible for international breast cancer clinical trials (41, 42) and to be responsive to adjuvant chemotherapy (CMF) (39, 41) and to endocrine therapy (40). Using the same approach will allow us to make comparisons across IBCSG trials, using the extensive QL data base that is now available from other trials.

10.2 Hypothesis

QL will be described in regard to intermediate- and long-term sequelae of treatment and disease, and the treatments will be compared in regard to QL. Specifically, the hypothesis to be investigated **is**:

- **Primary hypothesis**

Patients receiving low dose cytotoxics following induction chemotherapy will report QL reflecting greater toxicities and restrictions in daily life than those on the control arm. The following indicators are selected as primary endpoints: physical well-being, coping and thought of having treatment**.**

10.3 Additional analyses

Patients in the experimental arm undergoing cytotoxic therapy for an additional 12 months may possibly adapt to their disease and treatment differently than those in the standard arm. This process may include reframing of internal norms on which patients base their QL estimation, as described in patients receiving adjuvant chemotherapy for colon cancer (43). In the present trial, the significance to patients of changes in QL scores assessed by the LASA indicators will be determined by comparing the LASA change scores with verbal categories of subjective change (i.e., “Tattersall Index”) over different periods over time (see 10.6.2).

10.4 Patient Selection

QL assessments must be obtained from all patients randomized into this trial. The only exceptions are cognitive or physical impairment that interferes with QL assessment or inability to read any of the languages available on IBCSG QL forms.

10.5 Study Design

As in the other IBCSG trials with QL, a longitudinal design is used, including a pre-randomization assessment, assessments during CM maintenance to assess toxicity and assessments following CM maintenance to assess long-term effects, ~~and assessments following treatment failure to assess the impact of relapse.~~ To the extent feasible, the assessment time schedule is compatible with that of the other IBCSG trials to keep the logistics as simple as possible and to allow comparisons across trials. Patients are asked to complete a QL core form plus module before randomization. For those patients randomized before the first day of the last cycle, an additional QL core form plus module are required on the first day of the last cycle of induction chemotherapy. Regardless of randomized treatment assignment or timing of randomization, QL core form plus module are required at months 9, 12, 18 and 24~~; and a QL core form at yearly follow-up visits up to month 72~~ (see Figure 2). All patients, regardless of disease status, are to be assessed on the same schedule.

To eliminate any differential anticipatory effects on pre-randomization scores and to help insure compliance with the protocol requirements, a QL core form plus module must be completed *prior to* randomization.

Figure 2: Quality of Life Assessment Time Points
(revised per Amendment 4)

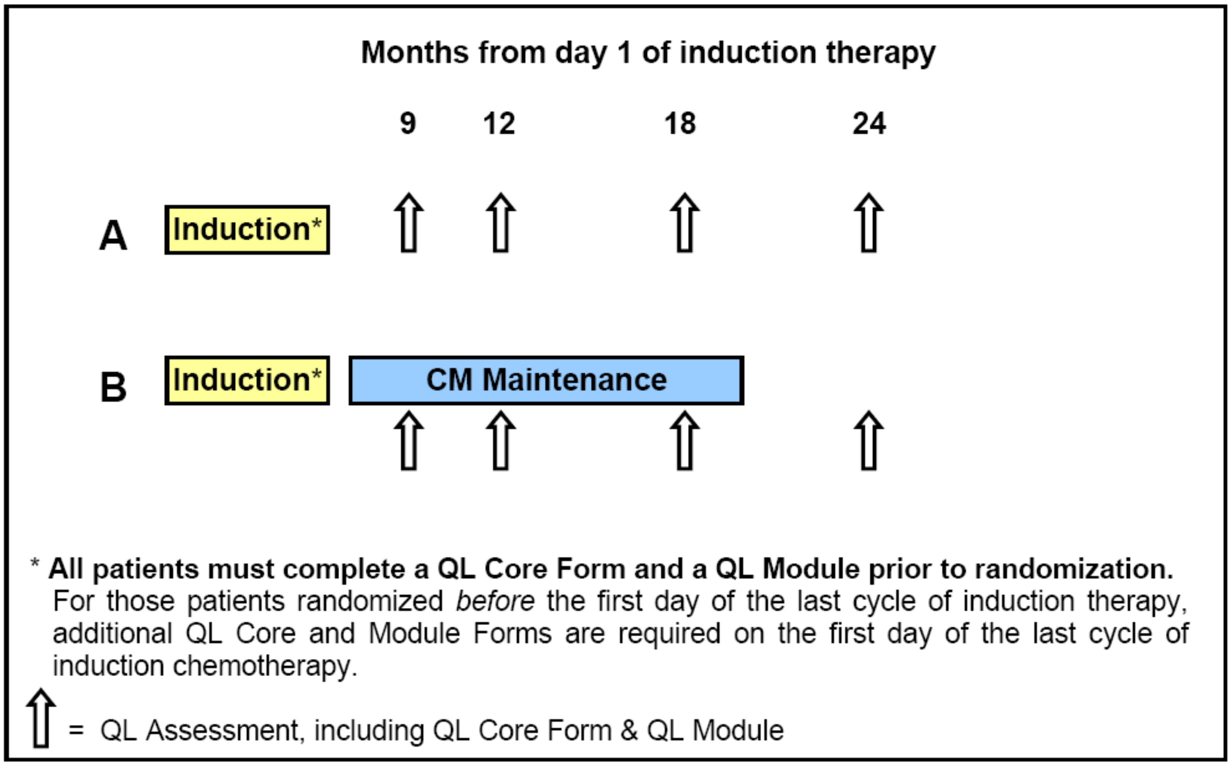

10.6 Quality-of-Life Measures

10.6.1 Patient-rated Quality of Life

The QL assessment consists of the IBCSG *QL core form* and a trial specific *module*.

The QL core form was developed in 1986 (39), and was subsequently revised for IBCSG Trials 10-93 through 14-93 (40), which started on May 1, 1993. The revised form is designed to address the endpoints

in adjuvant trials more specifically while still keeping the questionnaire simple and short. It includes global LASA indicators for physical well-being, mood, coping (PACIS), perceived social support and subjective health estimation (SHE). In addition, LASA indicators specific to symptoms of nausea and vomiting, tiredness, hot flushes, and restrictions in arm movement are included, covering possible QL effects of all of the treatment modalities involved in these trials (surgery, chemo-, endocrine and radiation therapy). Validation studies have been summarized elsewhere (40).

For the IBCSG CM maintenance trial, a specific one-page module has been developed. It includes all the GLQ-8 items (44) not covered by the IBCSG QL core form: hair loss, numbness, thought of actually having treatment, and loss of sexual interest or ability. The GLQ-8 was developed to evaluate QL in patients receiving chemotherapy (44). In addition, to investigate the significance to patients of changes in QL scores assessed by the LASA indicators, verbal categories of subjective change (i.e., “Tattersall Index”) will be included for the three primary endpoints (see 10.6.2).

As was previously done with the core form, the module was translated by an extensive "forward-backward" procedure to obtain not only linguistic but also conceptual equivalence (45).

In clinical trials, the distinction between indicators of specific symptoms and global indicators sensitive to treatment as well as disease-related problems in the broadest sense is very useful. In the global indicators, the score reflects a patient's *subjective*, intuitive choice and weighting of different aspects, summarized in a single response. Specific disease and treatment-related indicators can be used to examine the changing impact of symptoms on overall measures over time and in different situations (40). This can be done within a treatment group at specific points in time (e.g., for patients receiving CM maintenance therapy, how much of the variation in the overall physical well-being measure can be explained by the major side-effects during the period the patient is receiving additional chemotherapy, how much during the period following chemotherapy), within a treatment group across time (e.g., estimating the relative importance of tiredness over the first two years), or to explain differences in the global measures among treatment groups (e.g., does tiredness explain differences in physical well-being among treatment regimens at 12 months). The relationship between the specific and the global measures is also of methodological interest because it is an internal validity check: the percent of the variance explained by specific side-effects is expected to be higher during periods with toxic treatment as compared with periods without toxic treatment.

The conceptual basis of the IBCSG approach to QL assessment, along with a description of methodological issues, clinical findings and planned steps for further development, have been summarized elsewhere (40).

10.6.2 Response-shift and meaningful changes

Given that the patients in the experimental arm are to undergo cytotoxic therapy for additional 12 months, they may possibly adapt to their disease and treatment differently than those in the standard arm. In particular, they may change the internal norms on which they base their QL estimation. Such reframing or response-shift (46) was described in patients receiving adjuvant chemotherapy for colon cancer (43). A further objective in this trial is to investigate this process and to clarify whether it is relevant for treatment comparison regarding the definition of clinically meaningful changes. The methodology to evaluate this phenomenon is still in an experimental phase (47). Little is known about the temporal stability or variation of patients’ views on what constitutes a clinically meaningful change in QL measures. The role of these reframing effects will be investigated for the three primary QL endpoints (i.e., physical well-being, coping and thought of having treatment).

At the end of the QL module, the patient is asked to rate the change since the last time of filling in the QL form on a five-point categorical scale separately for each of the three primary endpoints (i.e., "Tattersall Index"). The interval between the two assessments is about 3 and 6 months during CM maintenance or observation. For the latter interval, it may be difficult for patients to estimate the past experience (48). Reframing may reflect memory effects. However, recall is not only dependent on time but also on the individual significance of disease and treatment. We would expect patients to indicate agreement or disagreement between the direction of change in their LASA scores and their verbal response in roughly equal proportions, if poor memory is the only factor influencing the retrospective estimations of change.

Although these verbal response categories of subjective change may also be affected by the response-shift as is the case for the LASA indicators (43), they are expected to be a reliable *internal* criterion of a change as experienced by the patient. This approach has been used to interpret changes in QL measures in numerous studies in different chronic diseases (49, 50, 51).

These patient-rated verbal categories of change will be associated with the changes in LASA scores assessed at the corresponding time points separately for each endpoint. We expect the association between changes in LASA scores and the verbal change category to be similar among the three endpoints, as it has been found in other studies for different QL domains (49, 50, 51). Given this is the case, these findings may be generalized for all of the LASA indicators. We also expect that the perception of change is different between the randomized treatments. In addition, physician-rated performance status and toxicity assessed at the same time points will be used as *external* criteria. Comparing the magnitude of the associations between changes in LASA scores and internal and external criteria within individual patients over time will give an estimate of the change of internal norms.

10.6.3 Utility considerations

One of the objectives of this trial is to obtain patient self-reported utility values to incorporate into a Q-TWiST analysis (Quality-adjusted Time Without Symptoms of disease and Toxicity of treatment) (52).

A patient rating of *subjective health estimation* (SHE) (53) in the LASA format is included in the QL core form. This utility scale anchored at "perfect health – worst health" was investigated in over 80 Swiss patients with metastatic breast cancer and was confirmed to be an appropriate measure for utility considerations within the setting of breast cancer clinical trials (53).

This approach is based on the hypothesis that patients may change their rating over time, in contrast to giving stable estimates as is assumed by other utility approaches. Furthermore, the relationship between the patient-rated utility and the other QL scales over time and in different treatments and language/cultural groups is of special clinical and methodological interest.

10.6.4 The Q-TWiST analysis

The Q-TWiST method (Quality-adjusted Time Without Symptoms of disease and Toxicity of treatment) compares treatments with respect to the weighted average of time spent in pre-defined clinical health states. The Q-TWiST health states partition the overall survival time into periods that differ with respect to QL. For evaluating adjuvant therapies for breast cancer, TOX (Ind CT) is the time spent with toxicity during induction chemotherapy and TOX (CM) is the time spent with CM treatment toxicity, TWiST is the time without disease recurrence and treatment toxicity, and REL is the time following breast cancer relapse.

$$\text{Q-TWiST (CM)} = u_{\text{TOX(Ind CT)}} \times \text{TOX (Ind CT)} + u_{\text{TOX(CM)}} \times \text{TOX (CM)} + \text{TWiST} + u_{\text{REL}} \times \text{REL}$$

Q-TWiST (OBS)= $u_{TOX(Ind\ CT)}$ x TOX (Ind CT) + TWiST + u_{REL} x REL

where $u_{TOX(Ind\ CT)}$, $u_{TOX(CM)}$, and u_{REL} are utility coefficients taking values between 0 (QL equivalent to death) and 1 (QL equivalent to TWiST).
Threshold utility analysis will provide the primary Q-TWiST treatment comparison (52). In addition, we will use the average utility coefficients obtained from patient questionnaires for each clinical health state. These values will be multiplied by the estimated duration of each clinical health state to obtain point estimates for Q-TWiST to use for treatment comparison.

10.6.5 Sociodemographics and comorbidity

As in the other IBCSG QL trials, sociodemographic data and co-morbidity are part of the standard study documentation.

10.7 Timing requirements, data collection and local data management

10.7.1 Timing requirements

Assessment time points following chemotherapy are determined by interval from day one of induction chemotherapy, and coincide with the required clinical follow-up time points (see Figure 1, Section 10.5).

The schedule of QL assessment must be followed as closely as possible. The QL form always has to be completed *prior to* diagnostic procedures and treatment administration to prevent any confounding effect. Analysis of baseline QL values in Trials VI and VII has shown that assessments done either before or after day 1 of CMF can substantially differ from those done on day 1 (54). The same is expected for the regimens used in this trial. Furthermore, it has to be assumed that for all subsequent assessments done at time points when patients are receiving chemotherapy, timing similarly affects the QL scores.

It is therefore important that every effort be made to follow the protocol exactly. If exact timing is not possible, assessment should be done as close as possible to the required date.

For methodological reasons, the required schedule should be followed precisely, with neither more nor fewer assessments. Shortly after randomization, the IBCSG Coordinating Center will send the local investigator a *schedule of the dates* of required QL assessments. This list should be put into each patient's chart to aid in the correct timing of the QL assessment.

10.7.2 Data collection and local data management

Within the first 24 months, every study patient is to fill in both the QL core form and the Trial 22-00 module at each scheduled assessment time point, as described in Figure 1, Section 5; no form selection is acceptable. ~~Thereafter, only the QL core form is required.~~

The QL forms are to be filled in at the clinic. If the patient is being followed elsewhere, arrangements are to be made with the clinic or physician to have the patient fill in the form(s) as required. If, for administrative reasons, the form has not been presented to the patient, it may be filled in at home and mailed. If a patient does not complete a scheduled QL assessment, a Missing Quality of Life Assessment Form (MQL) **must** be submitted for that assessment time point.

For the first assessment, the QL forms have to be explained to the patient, with particular emphasis on making sure the patient understands the LASA response format. For later assessments, the patient should be instructed to seek help only if she has problems in understanding any of the items in the form.

All questions on the core form and the module must be answered. Exceptions are the verbal categories for change at pre-randomization. The forms are to be checked after completion and, if necessary, *the patient should be asked to fill in missing answers*.
Detailed instructions for the QL assessment are given in the *IBCSG QL Manual*; copies can be downloaded from the IBCSG website (www.ibcsg.org).

10.8 Central data management

Computerized data quality control measures will be used to monitor the submission rates of the QL forms and the timing of assessment as required by the study protocol. Institutions will receive feedback on their performance and specific problems on a regular basis.

10.9 Statistical considerations

This phase III randomized clinical trial is designed to compare differences in QL between patients receiving a standard adjuvant treatment and those receiving low dose cytotoxics as “anti-angiogenesis treatment” following standard adjuvant chemotherapy.

The primary hypothesis will be tested by comparing the treatment groups using serial measurements of QL indicators over time (see 10.6.1).

The QL sample size will be based on the difference between the two treatment groups on the PACIS score evaluated at 6 months post induction therapy (9 months for the AC/EC group). The sample size will be selected to achieve an 80% statistical power to detect a difference in PACIS score of .5 units of the square root scale using a two-sided 0.05 level test. This difference corresponds to that observed in IBCSG Trial VI [41] at month 12 between Arms A (CMFx6) and B (CMFx6, plus reintroduction). This time point was the middle of the reintroduction. The standard deviation for the difference in square root of PACIS scores used in the sample size calculation also was obtained from Trial VI and equals 3.31. A sample size of 344 patients per treatment group with evaluable PACIS scores 6 months after the completion of induction therapy will be sufficient to achieve the stated objective.

11 Additional protocol-specific parameters and substudies

11.1 Hormone receptors

11.1.1 Hormone receptor determination

Immunohistochemical measurements of estrogen receptor (ER) and progesterone receptor (PgR) are required for this protocol. The measurement should be expressed as percent of positive cells. Patients with < 10% of cells positive are considered hormone receptor negative. For this trial, only patients with both ER-negative and PgR-negative tumors are eligible.

Of special interest in this trial is the comparison of treatments within the subgroup of patients with no positive hormone receptor cells (HR absent).

11.1.2 Quality assurance

It is mandatory that all laboratories conducting immunohistochemical measurements participate in a program for quality assurance. One such system is the NEQAS Scheme, which has been validated by the IBCSG pathologists.

More information on immunohistochemical measures and the NEQAS system is available in the Hormone Receptor Guidelines (Appendix III).

11.1.3 Central review

Tissue bank material will be used for central review of hormone receptors. The original histological report must be available.

11.2 Pathology and pathology material banking

11.2.1 Pathology requirements

The work of the pathologist is basic to the success of all studies. Each center should identify a pathologist responsible for study patients. The pathologist determines the diagnosis, classification, and grading of the primary tumor; and evaluates the non-tumor breast tissue and local or regional spread as found in the biopsy and/or mastectomy specimen, including precise documentation of tumor size, margins of the primary, the total number of lymph nodes examined, and the number of nodes involved. All lymph nodes must be examined from each patient. If the patient has received a sentinel node biopsy, each sentinel node must be evaluated. The central review pathologist will review the submitted specimens and complete the Central Pathology Review (CPR) form. See Appendix IV, “Pathology Guidelines” for more information.

The following items are required for all patients:

1. Completed Pathology Form P (signed by pathologist)
2. Pathology Report
3. Tumor block for banking
4. Normal tissue block for banking
5. Representative H & E sections of the above blocks

The tissue blocks, particularly in case of small tumors, may be returned to the participating center upon request.

All reports, slides, and blocks must be marked with the randomization number. If materials are not properly marked, we cannot guarantee that the slides will be forwarded to the Central Pathology Review Office. The Coordinating Center has received broken slides in the past and asks that they be sent in customized boxes especially made for slides. They should be packed with tissue paper to prevent any movement. The slides have not been packed securely enough if they move around when the box is shaken.

11.2.2 Pathology material banking

The IBCSG has established a central repository for tissue blocks and slides from every patient enrolled in IBCSG clinical trials. The required pathological material (described in the previous section) is submitted to, catalogued, and maintained in the Coordinating Center Office in Bern. The Australia-New Zealand Group will maintain a tumor bank within Australia. The H&E section is sent to the central review pathologist for central pathology review, and then returned to Bern for future storage. Central pathology review reports will be available to institution pathologists who wish to see them. The blocks will be available for prospective and retrospective studies approved by the Biological Protocols Working Group and by the IBCSG Ethical Committee.

11.3 c-erbB-2 (HER2)

The c-erbB-2 oncogene (HER2/neu) amplification and overexpression has been linked to higher transforming activity, increased metastatic potential, angiogenesis and drug resistance in breast tumor experimental models. Moreover, its overexpression present in 20-40% of breast cancer, is thought to be associated with a poor prognosis (55).
Immunohistochemical detection of the product of the c-erbB-2 oncogene (HER2/neu) (protein p185) is the standard method to evaluate the amplification and/or overexpression of the gene. The extracellular domain of the c-erbB-2 oncogene product, NEU-related protein (NRP), is detectable in serum by an ELISA method. This protein of 105kDA has been found elevated in patients with breast cancer (56). Many reports have correlated the elevated serum levels of the c-erbB-2 with gene amplification and c-erbB-2 overexpression in tumor (57, 58).

A substudy involving a subgroup of patients enrolled on this trial will evaluate serum levels of c-erbB-2 during maintenance treatment. This substudy will be an ancillary protocol.

11.4 Angiogenic activity

Angiogenesis is a critical element for solid tumor growth and metastasis, and in clinical studies active neovascularization in primary tumors has been associated with poor prognosis. This is particularly true for breast cancer.

Vascular Endothelial Growth Factor (VEGF) is known to play a key role in tumor angiogenesis. In a previous study on a small group of patients affected by metastatic breast carcinoma treated with methotrexate and cyclophosphamide VEGF was a good marker for following the clinical course of the illness.

The Trial 22-00 Serum Substudy will evaluate angiogenic activity for a subset of patients. The Substudy involves collecting serum samples at baseline, 12, 18, and 36 months and at disease progression. The samples will be analyzed for VEGF, VCAM-1 and NRP.

11.5 Family history

Information on patients' family history of breast cancer is being collected on Clinical Form B to evaluate its impact on prognosis. A positive family history of breast cancer has been shown to be associated with an increased risk of contralateral tumors (59) and second primaries (60). In addition, research is ongoing to determine whether genetically-associated breast cancer responds differently to treatment (61).

12 Regulatory approval procedures and patient informed consent

12.1 Ethical Review Board/Ethics Committee

All protocols and the patient informed consent forms must have the approval of a properly constituted committee or committees responsible for approving clinical trials. The ERB/IRB written, signed approval letter/form must contain approval of the designated investigator, the protocol (identifying protocol title and version number), and of the patient informed consent. Documentation of ethical committee approval must be sent to the IBCSG Coordinating Center prior to enrollment of the first patient.

12.2 Regulatory approval procedures

In addition to the approval of the Ethics Committee if applicable according to national legislation the protocol, other protocol related documents including patient information and informed consent and other documents as required locally must be submitted to and be approved by the health authority. Documentation of health authority approval must be sent to the IBCSG Coordinating Center prior to center activation.

12.3 Informed consent

Informed consent for each patient must be obtained prior to initiating any trial procedures (see Appendix I). One copy of the informed consent must be given to each patient, and the original must be retained in the investigator's trial records. The investigator's copy must be available for data audits.

The "Declaration of Helsinki" recommends that consent be obtained from each potential patient in biomedical research trials after the aims, methods, anticipated benefits, and potential hazards of the trial, and discomfort it may entail, are explained to the individual by the physician. The potential patient should also be informed of her right to not participate or to withdraw from the trial at any time. The patient must be informed of transfer and use of data, confidentiality, and alternative treatments. The patient should be told that material from her tumor will be stored and potentially used for additional studies not described in this protocol.

If the patient is in a dependent relationship to the physician or gives consent under duress, the informed consent should be obtained by an independent physician. If the patient is legally incompetent (i.e., mentally incompetent), informed consent must be obtained from the legal guardian, or legal representative in accordance with the law of the country in which the trial is to take place. By signing this protocol, the investigator agrees to conduct the trial in accordance with the "Declaration of Helsinki."

13 Administrative considerations

13.1 Insurance

IBCSG as the Sponsor of the Study contracts adequate Clinical Trial Insurance, in accordance with all relevant legal requirements, mandated by local regulations where the Study takes place. This insurance provides compensation to participants of the study.

Patients who suffer injuries due to the trial should report them immediately to their doctor.

The local group must report all alleged claims immediately to IBCSG.

14 References

1. Early Breast Cancer Trialists' Collaborative Group. Systemic treatment of early breast cancer by hormonal, cytotoxic, or immune therapy. 133 randomised trials involving 31000 recurrences and 24000 deaths among 75000 women. Lancet 339: 1-15 and 71-85, 1992

2. The International Breast Cancer Study Group. Duration and reintroduction of adjuvant chemotherapy for node positive premenopausal breast cancer patients. J Clin Oncol 14: 1885-1894, 1996

3. Fisher B, Brown AM, Dimitrov NV, Poisson R, Redmond C, Margolese RG, Bowman D, Wolmark N, Wickeham DI, Kardinal CG, et al. Two months of doxorubicin-cyclophosphamide with and without interval reinduction therapy compared with 6 months of cyclophosphamide, methotrexate, and fluorouracil in positive-node breast cancer patients with tamoxifen nonresponsive tumours: Results from the National Surgical Adjuvant Breast and Bowel Project B-15. J Clin Oncol 8: 1483-1496, 1990

4. Hutchins L, Green S, Ravdin P. CMF versus CAF with and without tamoxifen in high-risk node negative breast cancer patients: first results of intergroup trial INT 0102. Proc Am Soc Clin Oncol 17: 2, 1998

5. Levine MN, Bramwell VH, Pritchard KI, Norris BD, Shepherd LE, Abu-Zahra H, Findlay B, Warr D, Bowman D, Myles J, Arnold A, Vandenberg T, Mackenzie R, Robert J, Ottaway J, Burnell M, Williams CK, Tu D. Randomized trial of intensive Cyclophosphamide, Epirubicin, and Fluorouracil chemotherapy compared with cyclophosphamide, methotrexate, and fluorouracil in premenopausal women with node-positive breast cancer. J Clin Oncol 16: 2651-2658, 1998

5A. Henderson IC, Berry D, Demetri G, et al. Improved disease-free (DFS) and overall survival (OS) from the addition of sequential paclitaxel (T) but not from the escalation of doxorubicin (A) dose level in the adjuvant chemotherapy of patients (pts) with node-positive primary breast cancer (BC). Proc Am Soc Clin Oncol 1998; 17: 101.

5B. The French Adjuvant Study Group. Benefit of a high-dose epirubicin regimen in adjuvant chemotherapy for node-positive breast cancer patients with poor prognostic factors: 5-year follow-up results of French Adjuvant Study Group 05 randomized trial J Clin Oncol 2001;19(3):602-11.

5C. Goldhirsch A, Glick JH, Gelber RD, Coates AS, Thuerlimann B, Senn HJ. Meeting Highlights: International Expert Consensus on the Primary Therapy of Early Breast Cancer 2005. Ann Oncol 16:1569-1583, 2005.

6. Clahsen PC, van de Velde CJ, Welvaart K, Driel OJ, Sylvester RJ. Ten-year results of a randomized trial evaluating prolonged low-dose adjuvant chemotherapy in node-positive breast cancer: A joint European Organization for Research and Teatment of Cancer-Dutch Breast Cancer Working Party study. J Clin Oncol 13: 33-1341, 1995

7. Rivkin SE, Green S, Metch B, Cruz, AB, Abeloff MD, Jewell WP, Costanzi JJ, Farrar WB, Minton JP, Osborne CK. Adjuvant CMFVP versus tamoxifen versus concurrent CMFVP and tamoxifen for postmenopausal, node-positive, and estrogen receptor-positive breast cancer patients: A Southwest Oncology Group study. J Clin Oncol 12: 2078-2085, 1994

8. Folkman J. The influence of angiogenesis research on management of patients with breast cancer. Breast Cancer Res Treat 36: 109-118, 1995

9. Kern FG, Lippman ME. The role of angiogenic growth factors in breast cancer progression. Cancer Metastasis Rev 15: 213-219, 1996

10. Leek RD, Lewis CE, Whitehouse R, Greenall M, Clarke J, Harris AL. Association of macrophage infiltration with angiogenesis and prognosis in invasive breast carcinoma. Cancer Res 56: 4625-4629, 1996

11. Weidner N. Angiogenesis in breast cancer. Cancer Treat Res 83: 265-301, 1996

12. Sledge GW Jr. Implications of the new biology for therapy in breast cancer. Semin Oncol 23: 76-81, 1996

13. Gasparini G. Biological and clinical role of angiogenesis in breast cancer. Breast Cancer Res Treat 36: 103-107, 1995

14. Harris AL, Zhang H, Moghaddam A, Fox S, Scott P, Pattison A, Gatter K. Breast cancer angiogenesis: new approaches to therapy via antiangiogenesis, hypoxic activated drugs, and vascular targeting. Breast Cancer Res Treat 38: 97-108, 1996

15. Dickson RB, Johnson MD, Maemura M, Low J. Anti-invasion drugs. Breast Cancer Res Treat 38: 121-132, 1996

16. Salven P, Manpaa H, Orpana A, Alitalo K, Joensuu H. Serum vascular endothelial growth factor is often elevated in disseminated cancer. Clin Canc Res 3: 647-651, 1997

17. Toi M, Inada K, Suzuki H, Tominaga T. Tumor angiogenesis in breast cancer: its importance as a prognostic indicator and the association with vascular endothelial growth factor expression. Breast Cancer Res Treat 36: 193-204, 1995

18. Klauber N, Parangi S, Flynn E, Hamel E, D'Amato RJ. Inhibition of angiogenesis and breast cancer in mice by the microtubule inhibitors 2-methoxyestradiol and taxol. Cancer Res 57: 81-6, 1997

19. Billington DC. Angiogenesis and its inhibition: potential new therapies in oncology and non-neoplastic disease. Drug Design Discovery 8: 1-82, 1991

20. Steiner R. Angiostatic activity of anticancer agents in the chick allantoic membrane (CHE-CAM) assay. In: *Angiogenesis: Key Principles - Science - Technology - Medicine*, edited by R. Steiner, P. B. Weisz, and R. Langer, Basel:Birkhaeuser Verlag, 449-454, 1992

21. Hirata S, Matsubara T, Saura R, Tateishi H, Hirohata K. Inhibition of in vitro vascular endothelial cell proliferation and in vivo neovascularisation by low dose methotrexate. Arthritis Rheum 32: 1065-1073, 1989

22. Seitz M. Molecular and cellular effects of methotrexate. Curr Opin Rheumatol 11: 226-232, 1999

23. Shetty AK, Zganjar BE, Ellis GS Jr, Ludwig IH, Gedalia A. Low-dose methotrexate in the treatment of severe juvenile rheumatoid arthritis and sarcoid iritis. J Pediatr Ophthalmol Strabismus 36: 125-128, 1999

24. Dougados M, Combe B, Cantagrel A, Goupille P, Olive P, Schattenkirchner M, Meusser S, Paimela L, Rau R, Zeidler H, Leirisalo-Repo M, Peldan K. Combination therapy in early rheumatoid arthritis: a randomised, controlled, double blind 52 week clinical trial of sulphasalazine and methotrexate compared with the single components. Ann Rheum Dis 58: 220-225, 1999

25. Luis M, Pacheco-Tena C, Cazarin-Barrientos J, Lino-Perez L, Goycochea MV, Vazquez-Mellado J, Burgos-Vargas R. Comparison of two schedules for administering oral low-dose methotrexate (weekly versus every-other-week) in patients with rheumatoid arthritis in remission: a twenty-four week, single blind, randomized study. Arthritis Rheum 42: 2160-2165, 1999

26. Hirata S, Matsubara T, Saura R. Inhibition of in vitro vascular enthotelial cell proliferationand in vivo neovasularization by low-dose methotrexate. Arthritis Rheum 32: 1065-1069, 1989

27. Suzuki Y, Uehara R, Tajima C, Noguchi A, Ide M, Ichikawa Y, Mizushima Y. Elevation of serum hepatic aminotransferases during treatment of rheumatoid arthritis with low-dose methotrexate. Risk factors and response to folic acid. Scand J Rheumatol 28: 273-281, 1999

28. Rocca A, Colleoni M, Nolé F, Masci G, Orlando L, Zorzino L, Sandri M, Viale G, DeBraud F, Goldhirsch A. Low dose oral methotrexate and cyclophosphamide in metastatic breast cancer: antitumor activity and correlation with serum vascular endothelial growth cfactor (VEGF) levels. Proc Am Soc Clin Oncol 19: 460, 1999

29. Valagussa P, Moliterni A, Terenziani M, Zambetti M, Bonadonna G. Second malignancies following cmf-based adjuvant chemotherapy in resectable breast cancer. Ann Oncol 5: 803-808, 1994

30. Curtis RE, Boice JD Jr, Stovall M, Bernstein L, Greenberg RS, Flannery JT, Schwartz AG, Weyer P, Moloney WC, Hoover RN. Risk of leukemia after chemotherapy and radiation treatment for breast cancer. N Engl J Med 326: 1745-1751, 1992

31. Castiglione-Gertsch M, Johnsen C, Goldhirsch A, Gelber RD, Rudenstam CM, Collins J, Lindtner J, Hacking A, Cortes-Funes H, Forbes J, Simpson J, Tattersall MHN, Brunner K, Cavalli F, Senn HJ, for the International Breast Cancer Study Group (IBCSG). The International (Ludwig) Breast Cancer Study Group Trials I-IV: 15 years follow-up. Ann Oncol 5: 717-724, 1994

32. DeCillis A, Anderson S, Wickerham DL, Brown A, Fisher B, and contributing investigators. Acute myeloid leukemia (AML) in NSABP B-25. Proc Am Soc Clin Oncol 14: 92, 1995

33. Levine MN, Bramwell VH, Pritchard KI, Norris BD, Shepherd LE, Abu-Zahra H, Findlay B, Warr D, Bowman D, Myles J, Arnold A, Vandenberg T, MacKenzie R, Robert J, Ottaway J, Burnell M, Williams CK, Tu D. A randomized trial of cyclophosphamide, epirubicin, fluorouracil chemotherapy compared with cyclophosphamide, methotrexate, fluorouracil in premenopausal women with node positive breast cancer. J Clin Oncol 16: 2651-2658, 1998

34. The Scandinavian Breast Cancer Study Group. Results from a randomized adjuvant breast cancer study with high dose chemotherapy with CTCb supported by autologous bone marrow stem cells versus dose escalated and tailored FEC therapy. Proc Am Soc Clin Oncol 18: 3, 1999

34A. Piccart-Gebhart M: First results of the HERA trial; Romond E et al for the joint analysis of NSABP-B-31 and NCCTG-N9831; Perez E et. Al., for the NCCTG-N9831 collaboration; all presented at ASCO Meeting, Orlando, FL, May 16, 2005.

35. Kaplan EL, Meier P. Nonparametric estimation from incomplete observations. J Am Stat Assoc 53: 457-481, 1958

36. Cox DR. Regression models and life-tables. J R Stat Soc (B) 34: 187-220, 1972

37. O'Brien PC, Fleming TR. A multiple testing procedure for clinical trials. Biometrics 35: 549-556, 1979

38. Kim K, Tsiatis A. Study duration for clinical trials with survival response and early stopping rule. Biometrics 46: 81-90, 1990

39. Hürny C, Bernhard J, Gelber RD, Coates A, Castiglione-Gertsch M, Isley M, Dreher D, Peterson H, Goldhirsch A, Senn H-J, for the International Breast Cancer Study Group (IBCSG). Quality of life

measures for patients receiving adjuvant therapy for breast cancer: an international trial. Eur J of Cancer 28: 118-24, 1992

40. Bernhard J, Hürny C, Coates AS, Peterson HF, Castiglione-Gertsch M, Gelber RD, Goldhirsch A, Senn H-J, Rudenstam C-M, for the International Breast Cancer Study Group (IBCSG). Quality of life assessment in patients receiving adjuvant therapy for breast cancer: The IBCSG approach. Ann Oncol 8: 825-835, 1997

41. Hürny C, Bernhard J, Coates AS, Castiglione-Gertsch M, Peterson HF, Gelber RD, Forbes JF, Rudenstam C-M, Simoncini E, Crivellari D, Goldhirsch A, Senn H-J, for the International Breast Cancer Study Group (IBCSG). Impact of treatment on quality of life in patients with node-positive operable breast cancer. Lancet 347: 1279-1284, 1996

42. Bernhard J, Peterson HF, Coates AS, Gusset H, Isley M, Hinkle R, Gelber RD, Castiglione-Gertsch M, Hürny C, for the International Breast Cancer Study Group (IBCSG). Quality of life assessment in International Breast Cancer Study Group (IBCSG) Trials: Practical issues and factors associated with missing data. Stat Med 17: 587-601, 1998

43. Bernhard J, Hürny C, Maibach R, Herrmann R, Laffer U, for the Swiss Group for Clinical Cancer Research. Quality of Life as subjective experience: reframing of perception in patients with colon cancer undergoing radical resection with or without adjuvant chemotherapy. Ann Oncol 10: 775-782, 1999

44. Coates A, Glasziou P, McNeil D. On the receiving end - III. Measurement of quality of life during cancer chemotherapy. Ann Oncol 1: 213-217, 1990

45. Bernhard J, Hürny C, Coates A, Gelber RD. Applying quality of life principles in international cancer clinical trials. In: Spilker B, ed. Quality of life and pharmacoeconomics in clinical trials. Philadelphia, Lippincott-Raven 693-705, 1996

46. Sprangers MAG, Schwartz CE. The challenge of response shift for quality-of-life-based clinical oncology research. Ann Oncol 10: 747-749, 1999

47. Schwartz CE, Sprangers MAG. Methodological approaches for assessing response shift in longitudinal health-related quality-of-life research. Soc Sci Med 48: 1531-1548, 1999

48. Guadagnoli E, Cleary P. How consistent is patient-reported pre-admission health status when collected during and after hospital stay? Med Care 33: 106-112, 1995

49. Jaeschke R, Singer J, Guyatt GH. Measurement of health status. Ascertaining the minimal clinically important difference. Control Clin Trials 10: 407-415, 1989

50. Juniper EF, Guyatt GH, Willan A, Griffith LE. Determining a minimal important change in a disease-specific quality of life questionnaire. J Clin Epidemiol 47: 81-87, 1994

51. Osoba D, Rodrigues G, Myles J, Zee B, Pater J. Interpreting the significance of changes in health-related quality- of-life scores. J Clin Oncol 16: 139-144, 1998

52. Gelber RD, Goldhirsch A, Cavalli F. Quality-of-life-adjusted evaluation of adjuvant therapies for operable breast cancer. Ann Intern Med 114: 621-628, 1991

53. Hürny C, van Wegberg B, Bacchi M, Bernhard J, Thürlimann B, Real O, Perey L, Bonnefoi H, Coates A. Subjective health estimations (SHE) in patients with advanced breast cancer: an adapted utility concept for clinical trials. Br J Cancer 77: 985-991, 1998

54. Hürny C, Bernhard J, Coates A, Castiglione-Gertsch M, Peterson HF, Gelber RD, Rudenstam C-M, Goldhirsch A, Senn H-J, for the International Breast Cancer Study Group (IBCSG). Timing of baseline quality of life assessment in an international adjuvant breast cancer trial: Its effect on patient self-estimation. Ann Oncol 5: 65-74, 1994

55. Gusterson BA, Gelber RD, Goldhirsch A, Price KN, Säve-Söderborgh J, Anbazhagan R, Styles J, Rudenstam C-M, Golouh R, Reed R, Martinez-Tello F, Tiltman A, Torhorst J, Grigolato P, Bettelheim R, Neville AM, Bürki K, Castiglione M, Collins J, Lindtner J, Senn H-J for the International (Ludwig) Breast Cancer Study Group. Prognostic importance of c-*erb*B-2 expression in breast cancer. J Clin Oncol 10: 1049-1056, 1992

56. Streckfus C, Bigler L, Dellinger T, Dai X, Kingman A, Thigpen JT. The presence of soluble c-erbB2 in saliva and serum among women with breast carcinoma: a preliminary study. Clin Cancer Res 6: 2363-2370, 2000

57. Krainer M, Brodowicz T, Zeillinger R, Wiltschke C, Scholten C, Seifert M, Kubista E, Zielinski CC. Tissue expression and serum levels of HER2/neu in patients with breast cancer. Oncology 54: 475-481, 1997

58. Wu JY, Astill ME, Gagon SD, Bryson L. Measurement of c-erbB2 proteins in sera from patients with carcinomas and in breast tumor tissue cytosols: correlation with serum tumor markers and membrane-bound oncoprotein. J Clin Lab Anal 9: 151-165, 1995

59. Chabner E, Nixon A, Gelman R, Hetelekidis S, Recht A, Bornstein B, Connolly J, Schnitt S, Silver B, Manola J, Harris J, Garber J. Family history and treatment outcome in young women after breast-conserving surgery and radiation therapy for early-stage breast cancer. J Clin Oncol 16: 2045-2051, 1998

60. Brekelmans CT, Voogd AC, Botke G, van Geel BN, Rodrigues P, Rutgers EJ, Klijn JG, Coebergh JW. Family history of breast cancer and local recurrence after breast-conserving therapy. The Dutch Study Group on Local Recurrence after Breast Conservation (BORST). Eur J Cancer 35: 620-626, 1999

61. Phillips KA, Andrulis IL, Goodwin PJ. Breast carcinomas arising in carriers of mutations in BRCA1 or BRCA2: are they prognostically different? J Clin Oncol 17: 3653-3663, 1999
