# Supplementary material for: Role of immunosuppressive JNK pathway in the tumor microenvironment among TNBC subtypes in IBCSG trial 22-00
Source: iScience. 2025 Jun 20;28(8):112964. doi: 10.1016/j.isci.2025.112964 (PMC12355117; doi:10.1016/j.isci.2025.112964)
Supplement: Data S2. IBCSG 22-00 clinical trial information [file mmc3.zip › CONSORT_Checklist_IBCSG.doc]

CONSORT 2010 checklist of information to include when reporting a randomised trial*

| Section/Topic | Item No | Checklist item | Reported on page No |
| --- | --- | --- | --- |
| Title and abstract | | | |
|  | 1a | Identification as a randomised trial in the title | n/a, in this retrospective analysis we focused on one of the TNBC cohort from the IBCSG 22-00 randomized clinical trial |
|  | 1b | Structured summary of trial design, methods, results, and conclusions (for specific guidance see CONSORT for abstracts) | 2, retrospective translational research analysis (all the details of the trials have been previously published in the original publications for IBCSG22-00 trial) |
| Introduction | | | |
| Background and objectives | 2a | Scientific background and explanation of rationale | 3 |
|  | 2b | Specific objectives or hypotheses | 3 |
| Methods | | | |
| Trial design | 3a | Description of trial design (such as parallel, factorial) including allocation ratio | 5, Reporting summary, previous publications cited |
|  | 3b | Important changes to methods after trial commencement (such as eligibility criteria), with reasons | 5, previous publications cited |
| Participants | 4a | Eligibility criteria for participants | 5, reporting summary, previous publications cited |
|  | 4b | Settings and locations where the data were collected | 5, reporting summary |
| Interventions | 5 | The interventions for each group with sufficient details to allow replication, including how and when they were actually administered | 5, reporting summary |
| Outcomes | 6a | Completely defined pre-specified primary and secondary outcome measures, including how and when they were assessed | 5, reporting summary |
|  | 6b | Any changes to trial outcomes after the trial commenced, with reasons | 5, reporting summary |
| Sample size | 7a | How sample size was determined | n/a, retrospective translational research correlative analysis on a case-control cohort like sampling (1:3 ratio). |
|  | 7b | When applicable, explanation of any interim analyses and stopping guidelines | n/a, retrospective translational research correlative analysis |
| Randomisation: |  |  |  |
| Sequence generation | 8a | Method used to generate the random allocation sequence | n/a, retrospective translational research correlative analysis, reporting summary |
|  | 8b | Type of randomisation; details of any restriction (such as blocking and block size) | n/a, retrospective translational research correlative analysis, reporting summary |
| Allocation concealment mechanism | 9 | Mechanism used to implement the random allocation sequence (such as sequentially numbered containers), describing any steps taken to conceal the sequence until interventions were assigned | n/a, retrospective translational research correlative analysis |
| Implementation | 10 | Who generated the random allocation sequence, who enrolled participants, and who assigned participants to interventions | n/a, retrospective translational research correlative analysis, reporting summary |
| Blinding | 11a | If done, who was blinded after assignment to interventions (for example, participants, care providers, those assessing outcomes) and how | n/a, retrospective translational research correlative analysis – no blinding for the present analyses |
|  | 11b | If relevant, description of the similarity of interventions | n/a, retrospective translational research correlative analysis |
| Statistical methods | 12a | Statistical methods used to compare groups for primary and secondary outcomes | 8, Supplementary methods 5 |
|  | 12b | Methods for additional analyses, such as subgroup analyses and adjusted analyses | 8, Supplementary methods 5 |
| Results | | | |
| Participant flow (a diagram is strongly recommended) | 13a | For each group, the numbers of participants who were randomly assigned, received intended treatment, and were analysed for the primary outcome | Already published in Joaquin Garcia et al., 2023. |
|  | 13b | For each group, losses and exclusions after randomisation, together with reasons | Supplementary figure 1 |
| Recruitment | 14a | Dates defining the periods of recruitment and follow-up | 4,5 |
|  | 14b | Why the trial ended or was stopped | n/a, retrospective translational research correlative analysis. |
| Baseline data | 15 | A table showing baseline demographic and clinical characteristics for each group | Already published in Joaquin Garcia et al., 2023. |
| Numbers analysed | 16 | For each group, number of participants (denominator) included in each analysis and whether the analysis was by original assigned groups | Already published in Joaquin Garcia et al., 2023. |
| Outcomes and estimation | 17a | For each primary and secondary outcome, results for each group, and the estimated effect size and its precision (such as 95% confidence interval) | Already published in Joaquin Garcia et al., 2023. |
|  | 17b | For binary outcomes, presentation of both absolute and relative effect sizes is recommended | Already published in Joaquin Garcia et al., 2023. |
| Ancillary analyses | 18 | Results of any other analyses performed, including subgroup analyses and adjusted analyses, distinguishing pre-specified from exploratory | 9,10,11,12,13 |
| Harms | 19 | All important harms or unintended effects in each group (for specific guidance see CONSORT for harms) | n/a, retrospective translational research correlative analysis (no analyses on the treatment impact, reported elsewhere in previous publications cited). |
| Discussion | | | |
| Limitations | 20 | Trial limitations, addressing sources of potential bias, imprecision, and, if relevant, multiplicity of analyses | 17 |
| Generalisability | 21 | Generalisability (external validity, applicability) of the trial findings | 16,17 |
| Interpretation | 22 | Interpretation consistent with results, balancing benefits and harms, and considering other relevant evidence | 14,15,16 |
| Other information | | |  |
| Registration | 23 | Registration number and name of trial registry | 5 |
| Protocol | 24 | Where the full trial protocol can be accessed, if available | n/a, retrospective |
| Funding | 25 | Sources of funding and other support (such as supply of drugs), role of funders | 18 |

Citation: Schulz KF, Altman DG, Moher D, for the CONSORT Group. CONSORT 2010 Statement: updated guidelines for reporting parallel group randomised trials. BMC Medicine. 2010;8:18.
© 2010 Schulz et al. This is an Open Access article distributed under the terms of the Creative Commons Attribution License (<http://creativecommons.org/licenses/by/2.0>), which permits unrestricted use, distribution, and reproduction in any medium, provided the original work is properly cited.

*We strongly recommend reading this statement in conjunction with the CONSORT 2010 Explanation and Elaboration for important clarifications on all the items. If relevant, we also recommend reading CONSORT extensions for cluster randomised trials, non-inferiority and equivalence trials, non-pharmacological treatments, herbal interventions, and pragmatic trials. Additional extensions are forthcoming: for those and for up-to-date references relevant to this checklist, see [www.consort-statement.org](http://www.consort-statement.org/).
